# Genetic basis of job attainment characteristics and the genetic sharing with other SES indices and well-being

**DOI:** 10.1038/s41598-022-12905-y

**Published:** 2022-05-26

**Authors:** Zhaoli Song, Wen-Dong Li, Hengtong Li, Xin Zhang, Nan Wang, Qiao Fan

**Affiliations:** 1 Department of Management and Organization, National University of Singapore, Singapore, Singapore; 2 Department of Management, The Chinese University of Hong Kong, Hong Kong, China; 3 Department of Statistics and Data Science, National University of Singapore, Singapore, Singapore; 4 Department of Management, Lingnan University, Hong Kong, China; 5 Centre for Quantitative Medicine, Duke-NUS Medical School, Singapore, Singapore

**Keywords:** Behavioural genetics, Genetic association study, Psychology, Diseases

## Abstract

Job attainment is an important component of socioeconomic status (SES). There is currently a paucity of genomic research on an individual’s job attainment, as well as how it is related to other SES variables and overall well-being at the whole genome level. By incorporating O*NET occupational information into the UK Biobank database, we performed GWAS analyses of six major job attainment characteristics—job complexity, autonomy, innovation, information demands, emotional demands, and physical demands—on 219,483 individuals of European ancestry. The job attainment characteristics had moderate to high pairwise genetic correlations, manifested by three latent factors: cognitive, emotional, and physical requirements. The latent factor of overall job requirement underlying the job attainment traits represented a critical genetic path from educational attainment to income (*P* < 0.001). Job attainment characteristics were genetically positively correlated with positive health and well-being outcomes (i.e., subject well-being, overall health rating, number of non-cancer illnesses etc. (|*r*_*g*_|: 0.14–0.51), similar to other SES indices; however, the genetic correlations exhibited opposite directions for physical demands (|*r*_*g*_|: 0.14–0.51) and were largely negligible for emotional demands. By adopting a finer-grained approach to capture specific job attainment phenotypes, our study represents an important step forward in understanding the shared genetic architecture among job attainment characteristics, other SES indices, and potential role in health and well-being outcomes.

## Introduction

Work represents an indispensable area of life for most individuals in modern societies. Job attainment, therefore, is regarded as a crucial component of socioeconomic status (SES) that has been widely investigated as an important predictor of health and well-being^1,2^. SES has been conceptualized and examined as a composite of educational attainment, occupation or job attainment, and income to reflect one’s social status^3–5^. Job attainment has been suggested as a critical structural link between educational attainment and income^6^. Current genetic research, however, has predominantly focused on educational attainment and income^7,8^, resulting in the limited understanding of the whole spectrum of SES and their relations with well-being. The current study aims to offer insight into the genetic architecture of specific job attainment characteristics, as well as the shared genetic basis between job attainment with other SES indices, and well-being, and thus may contribute to the literature on SES and occupational health research.

Job attainment characteristics have been mainly investigated in the literature as work contextual factors that influence individuals’ psychological and physical well-being^9,10^. The dominant perspective assumes that the characteristics of the job that one holds are mostly determined by environmental factors, such as occupational and organizational contexts, and thus has overlooked the possible role of genetic factors through processes of self selection and occupational selection^10,11^. Recently, twin studies have demonstrated that genetic factors explained a substantial portion of the variance in one’s job attainment characteristics such as job demand, autonomy, and complexity, with heritability estimates at approximately 30%^12^. Furthermore, phenotypical correlations between job characteristics and well-being were partially explained by their shared genetic influences^12^.

In this study, we first conducted GWAS analyses on six commonly examined job attainment characteristics: job complexity, autonomy, innovation, and three types of job demands (information demands, emotional demands, and physical demands), which represent various aspects of job attainment^13–16^. We constructed the phenotypes using information from the U.S. Occupational Information Network (O*NET), a comprehensive occupational dataset that has been used across countries^17,18^, by matching the Standard Occupational Codes in O*NET to the occupational codes adopted in the UK Biobank. We performed genetic exploratory factor analysis among the job attainment characteristics, and genetic sharing among the three SES variables: education attainment, job attainment, and income. We conducted genomic structure equation modeling and path analyses to examine relationships among these SES variables at the whole genome scale. Furthermore, we explored genetic correlations between job attainment characteristics and major health and well-being outcomes, accounting for intelligence—a common factor shared among SES variables.

## Results

### Phenotypical measures of job characteristics

The job demand-control model^19,20^, a dominant model proposed for occupational health and job design, posits that job demands and job autonomy/control are crucial job characteristics that influence workers’ health and well-being. Meanwhile, omnibus characteristics (e.g., difficulty levels indicated by job complexity) also constitute an important aspect^21^. However, such information was not directly recorded in the UK Biobank data. We thus linked the four-digit Standard Occupational Codes in the UK Biobank data to occupation codes from O*NET to extract useful information on both omnibus and concrete job attainment characteristics. Six commonly used job attainment characteristics from the O*NET database were assessed: job complexity, autonomy, innovation, and three types of job demands (information demands, emotional demands, and physical demands) (Box 1)^22^. Complexity, highly correlated to occupational prestige, is an measure to assess how difficult and challenging to perform a job^23,24^. The other five assess different specific aspects of the job characteristics.

Except for the innovation, which was measured with one item, the internal consistency was high for each phenotype (Cronbach’s α Coefficients ranging from 0.80 to 0.98; Table 1). The detailed information of items for job attainment phenotype was listed in Supplementary Table S1. The phenotype measures were presented as the average score across all relevant items, with a higher score implying a higher level of requirement or attainment for the corresponding job characteristics. In Box 1, we listed the top occupations with the highest score for each job attainment characteristic.

**Box 1:** Definitions of six job attainment characteristics and sample job titles.

Phenotype	Definition	Sample job titles
Complexity	Job complexity refers to the degree to which a job is difficult and mentally challenging to perform. It has been widely examined as an omnibus work characteristic that influences not only one’s well-being^92^, but also been utilized as an indicator of occupational status^53^	Aircraft pilots and flight engineers Chemical engineers Mechanical engineers
Autonomy	Job autonomy is defined as the amount of autonomy that one has at work to decide what to do, when to do and how to do his or her work^93^	Directors and chief executives of major organizations Production, works and maintenance managers, Higher education teaching professionals
Innovation	Innovation encompasses to what extent one’s job requires him or her to generate novel and useful ideas^75^	Architects Product, clothing, and related designers Graphic designers Artists
Information demands	Requirements to work with data and information, such as processing information, analyzing data, reasoning, interacting with computers^14^	IT strategy and planning professionals Information and communication technology managers Physicists, geologists, and meteorologists
Emotional demands	Demands for emotional labor tasks such as assisting and helping others and dealing with unpleasant people^14^	Paramedics Air travel assistants Nurses
Physical demands	Demands for manual labor as well as risks and unpleasant job conditions such as dangerous working conditions and loud noises^14^	Metal plate workers Shipwrights, riveters Coal mine operatives

**Table 1:** Summary of O*NET linked phenotype scores of six job attainment characteristics in the UK Biobank data (N = 219,483).

Phenotype	Mean (s.d)	Range	Items	Cronbach’s α
Complexity	2.13 (0.34)	[1.13, 3.15]	120	0.98
Autonomy	4.08 (0.36)	[2.69, 4.95]	2	0.90
Innovation	3.54 (0.42)	[2.11, 4.62]	1	–
Information demands	3.55 (0.79)	[1.53, 5.25]	17	0.90
Emotional demands	3.22 (0.61)	[1.54, 4.76]	5	0.80
Physical demands	1.82 (0.70)	[0.94, 4.31]	12	0.96
Age	54.35 (7.67)	[39, 71]		
Male, %	50.50%			

No Cronbach’s α was calculated for innovation as only one item is included.

### GWAS for each phenotype

**Figure 1:**
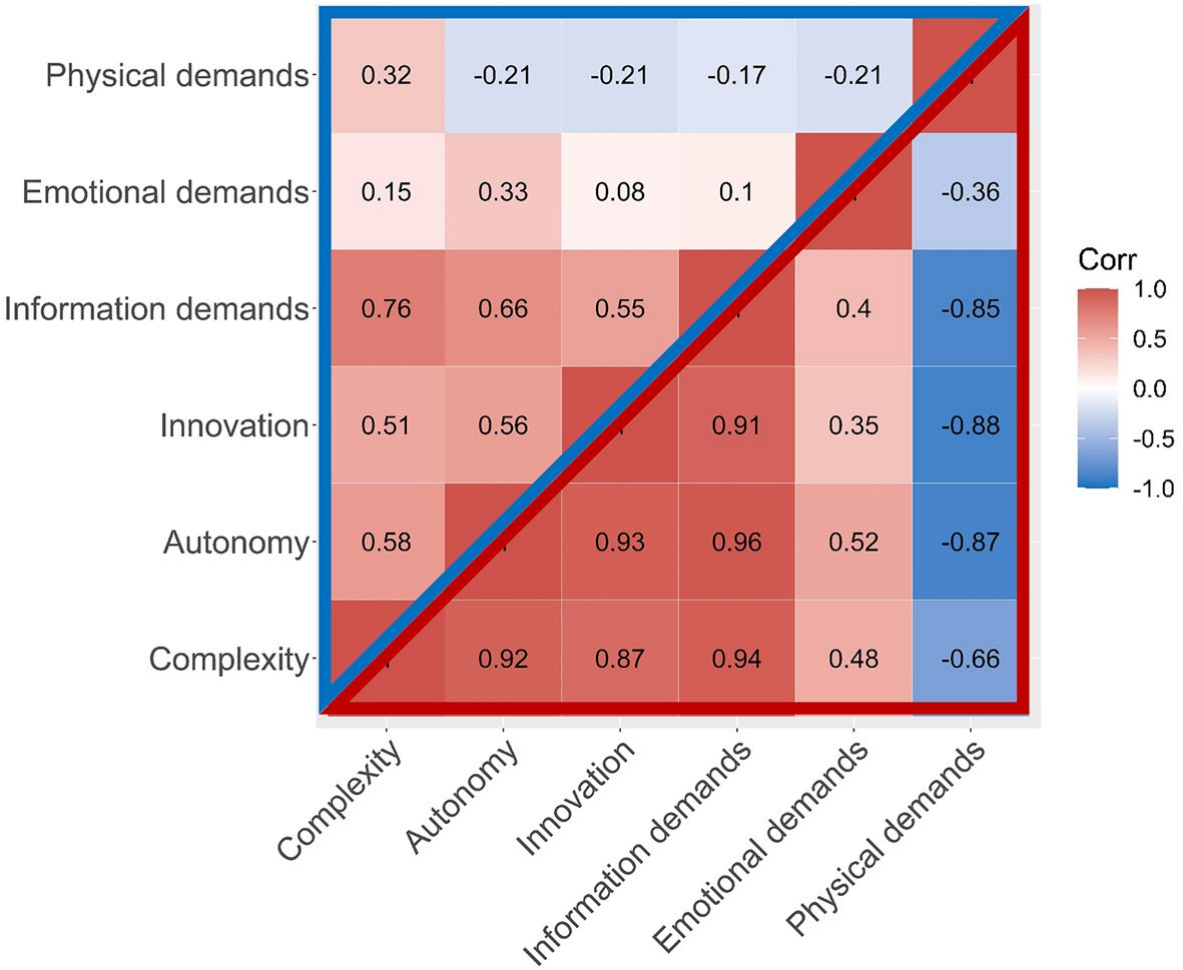
Pairwise phenotypic and genetic correlations among six job attainment characteristics in UK Biobank data. The upper-left triangular correlation matrix is the pairwise phenotypic correlation and the lower-right is the genetic correlation matrix. Phenotypic correlation, Pearson correlation coefficient, was calculated based on the UK biobank participants included in this study (n = 219,483). Genetic correlation was calculated based on the GWAS summary statistics using LDSC method from the same set of participants. The correlation coefficient is shown in each sub-box. Positive correlation is displayed in red and negative correlation in blue.

In our analysis, we included 219,483 European ancestry participants in the UK Biobank with matched codes from the O*NET database (Supplementary Fig. S1). Among all participants, the average age was 54.4, and 50.5% were male. Phenotypically, complexity, autonomy, innovation, and information demands were substantially positively correlated with each other (Pearson correlation *r* from 0.51 to 0.76; Fig. 1: upper-left). Emotional demands had small to moderate positive correlations with other job characteristics (*r* 0.08–0.33). Physical demands were negatively correlated with other job characteristics (*r* − 0.21 to − 0.33), except for job complexity (*r* 0.32).

We performed GWAS for each job attainment trait on 19,400,443 genetic variants passed quality control (QC). The genomic control inflation factor λ_GC_ ranged from 1.047 to 1.147 (Supplementary Table S2; Supplementary Fig. S2). The intercepts estimated from the linkage disequilibrium score (LDSC) regression^25^ were close to 1 (LDSC intercept 1.009–1.043), indicating that the slight inflation of λ_GC_ was due to the presence of polygenic signals, rather than population stratification or cryptic relatedness^26^. Thus, no further adjustment for λ_GC_ was performed in our analyses.

A total of 16 loci associated with the job attainment characteristics were identified at genome-wide significance, with four loci shared by at least two traits (*P* < 8.33 × 10^−9^ after multiple testing correction; Supplementary Table S3 and Supplementary Figs. S3, S4). Among these, a total of 3 loci showed genome-wide significance with complexity, 3 with autonomy, 3 with innovation, 6 with information demands, and 8 with physical demands. The strongest signal was a pleiotropic locus at rs6446187 (*P* = 1.90 × 10^–16^) on chromosome 3 for information demands, autonomy, innovation, and complexity. Genetic effects of the top variants in the age subgroup are presented in Supplementary Table S4 (age ≤ 55, N = 118,024; age > 55, N = 111,059). Among the 23 variants at 16 loci, 3 showed marginally different genetic effects between age subgroups (*P_dif* < 0.05). After accounting for multiple testing, none of these SNPs remained significant at *P_dif* < 3.13 × 10^–3^. We evaluated associations of these identified variants for job attainment in the UK Biobank follow-up data (N = 30,837) and the Add Health Wave V data (N = 3817). Lead SNPs at the four loci showed nominal significance at *P* value less than 0.05 (Supplementary Table S5). The direction of effects for the lead SNPs was concordant in the UK Biobank follow-up sample (81.8%). The moderate concordance of effects’ direction in the Add Health study (63.6%) may be due to the small sample size and the heterogeneity of participants, such as the younger population, in contrast to the UK Biobank data.

### Heritability and genetic correlations among job attainment phenotypes

Common variant heritability *h*^2^, defined as the amount of phenotypic variance that could be explained by the additive genetic effects of all common SNPs, was estimated from 1.6 to 7.7% using the LDSC regression, lowest for emotional demands while highest for information demands (Supplementary Table S2). All these *h*^2^ estimates were significantly different from zero (*P* < 0.001). These estimates were in line with the reported estimates for social and behavioral phenotypes such as risk tolerance at 4.6%^27^, household income at 7.4%^7^, neuroticism at 10.8%^28^, but substantially lower than that for intelligence at 19%^29^.

We calculated genetic correlations among these six phenotypes^30^. We found that pairwise genetic correlations (*r*_*g*_) were strong and positive from 0.87 to 0.96 for complexity, autonomy, innovation, and information demands (Fig. 1: lower-right). Emotional demands had smaller positive correlations with these four traits from 0.35 to 0.52. Physical demands had negative genetic correlations with these four traits ranging from − 0.66 to − 0.88, and a weaker negative correlation of − 0.36 with emotional demands. The genetic correlations were larger than the corresponding phenotypical correlations; attenuation in phenotypic correlations suggests the major influences on the job attainment phenotypes could result from the heterogeneity of environmental factors^27^.

### Between-sex genetic correlation and sex-specific genetic effect sizes

We first performed GWAS for each job attainment phenotype in males and females separately. Based on sex-specific GWAS summary statistics, we estimated between-sex genetic correlations (*r*_*g*_) within each job attainment trait using LDSC regression. Between-sex genetic correlations were moderate to high for autonomy (*r*_*g*_ = 0.90), innovation (*r*_*g*_ = 0.92), information demands (*r*_*g*_ = 0.90), emotional demands (*r*_*g*_ = 0.76) and physical demands (*r*_*g*_ = 0.88), with the lowest for complexity (*r*_*g*_ = 0.52; see Supplementary Table S6). All these genetic correlations were significantly lower than 1, except for complexity (*P* = 1.40 × 10^–7^), indicating a common genetic variant largely shared between males and females for these five phenotypes.

To detect SNPs with the different effect sizes across sexes from sex-stratified regression coefficients, we further computed per-SNP z scores of those top index SNPs showing genome-wide significance at least in one sex for each phenotype. After accounting for multiple testing (*P*_dif < 1.32 × 10^–3^), the associations were present in females, but not significant in males, for complexity, autonomy, and innovation (one locus respectively), and information demands (three loci; Supplementary Table S7). Conversely, 14 loci were identified in males only, with 12 loci for physical demands and two for information demands. We also computed z scores for all SNPs across whole genome (Supplementary Fig. S5 QQ-plots). At the genome-wide scale, two loci exhibited opposite genetic effects in males and females (*P*_dif < 8.30 × 10^–9^), but none showed genome-wide significance at least in one sex. The underlying mechanisms for these loci and these sex differences remain to be explored. It is warranted to futher validate these top loci showing different gender effects in future research.

### Latent factors for the six job attainment phenotypes

Emerging evidence suggests that major job attainment characteristics can be manifested by different latent factors^14,31,32^. We performed both phenotypical and genetic exploratory factor analyses (EFA)^33^ to investigate the possible latent factor structure of these six job attainment characteristics. Both phenotypic and genetic EFAs generated three latent factors (Table 2), which are related to cognitive, emotional (or named social), and physical requirements to perform a job^14,31,32^. The first top factor represented cognitive job requirements; the explained variance was slightly higher in genetic EFA than phenotypical EFA (79% vs. 72%). Meanwhile, in genetic EFA, the cognitive job requirement factor was related to all the six job attainments. The physical requirement factor was a stand-alone factor in the phenotypic EFA, and it contributed substantially negatively to the cognitive job requirement factor in genetic EFA (factor loading in genetic vs. phenotypic EFA − 0.89 vs. − 0.12). Altogether, three latent factors explained 98% of the genetic variation across the six traits.

**Table 2:** Phenotypic and genetic EFAs of six job attainment characteristics in the UK Biobank discovery data.

Variables	Phenotypical EFA			Genetic EFA		
	Cognitive job requirement	Physical job requirement	Emotional job requirement	Cognitive job requirement	Physical job requirement	Emotional job requirement
Complexity	**0.84**	**0.45**	0.07	**0.93**	0.37	0.02
Autonomy	**0.77**	− 0.17	0.15	**0.99**	0.01	0
Innovation	**0.66**	− 0.12	− 0.14	**0.95**	− 0.04	− 0.19
Information demands	**0.88**	− 0.01	− 0.20	**0.97**	0.11	− 0.13
Emotion demands	0.24	− 0.24	**0.43**	**0.53**	− 0.09	**0.84**
Physical demands	− 0.12	**0.82**	0.09	− **0.89**	**0.42**	0.18
Proportion	0.72	0.27	0.08	0.79	0.06	0.13

Factor loadings are standardized; factor loading higher than 0.40 are highlighted in bold.

*EFA* Exploratory factor analysis.

### The predictive power of Polygenic Scores (PGSs)

To test whether the aggregate estimates of genetic effects correlated with job attainment, we constructed PGS for each job attainment trait in the UK Biobank follow-up and Add Health data. A series of cut-off *P* value thresholds for variants were examined and the optimal model for each job attainment trait was selected based on the *R*^2^. PGSs were significantly associated with job attainment levels for all the six phenotypes in the UK Biobank follow-up sample (*P* ranged from 2.56 × 10^–62^ to 3.05 × 10^–13^; Supplementary Table S8), and in the Add Health sample (*P* ranged from 1.45 × 10^–10^ to 9.79 × 10^–4^), suggesting the robustness of the GWAS findings. PGS accounted for phenotypic variance in a range of incremental R^2^ from 0.15 to 0.91%, adjusting for age, sex, and top PCs, indicating that PGS alone had significant but small predictive value for the job attainment phenotypes in the two replicaion samples. Yet, this does not necessarily mean that PGSs were not useful, because the findings reflected only main effects of the genetic variants. Future research may examine how PGSs interact with environmental factors in shaping job characteristics and the nomological networks for them beyond job characteristics.

### Pathway and expression patterns of the implicated loci

We performed pathway analyses for the implicated genes and identified 19 significant pathways at multiple testing corrected *P* value of 0.05 (Supplementary Table S9). Among these, neuron related pathways were largest enriched (9 of 19 pathways), consisting of the most significant pathway regulation of synapse organization (GO: 0050808; *P* = 1.76 × 10^–4^), followed by cell morphogenesis involved in neuron differentiation (GO: 0048667; *P* = 1.75 × 10^–3^) and regulation of synapse organization (GO: 0050807; *P* = 3.38 × 10^–3^).

**Figure 2:**
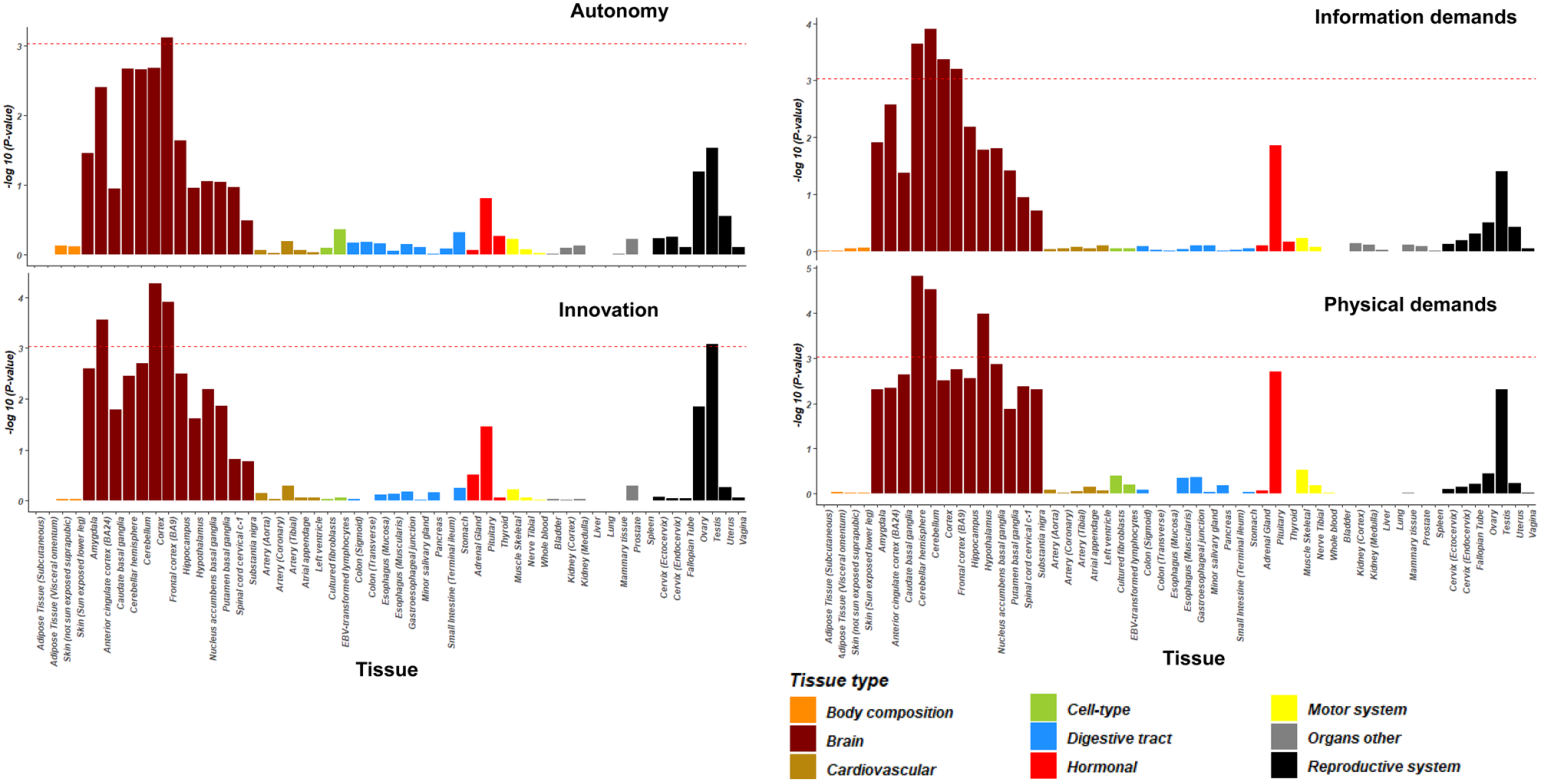
Enrichment of gene expression in tissues for autonomy, innovation, information demands, and physical demands. The x axis presents 54 specific tissues (the tissue order is the same for each sub-plot), and the y axis presents − log_10_ *P* values for enrichment of implicated genes in specific tissues for job attainment characteristics. Colors represent a broad category of different tissue types. The horizontal red dashed line represents a Bonferroni significance level of *P* < 9.26 × 10^–4^ ; the detailed information on beta effects in Supplementary Table S7).

We also assessed gene expression in different human tissues based on the expression of the quantitative trait loci (e-QTL) database^34^. Except for complexity and emotional demands, the gene expression was significantly enriched in the brain for job autonomy, innovation, information demands, and physical demands (Fig. 2; Supplementary Table S10). The specific brain regions for the gene expression enrichment were similar but with subtle differences for different traits. For example, for innovation, the expression enrichment was in brain cortex regions (Frontal and anterior; *P* ≤ 2.75 × 10^–4^) and testis (*P* = 8.38 × 10^–4^). It has been documented in the literature that the testosterone hormone secreted from testes is related to creativity, entrepreneurial intention, and behavior^35,36^. For physical demands, the expression enrichment was in the cerebellum/cerebellum hemisphere (*P* ≤ 3.03 × 10^–5^) and hypothalamus (*P* = 1.05 × 10^–4^), which are related to physical and motor regulations. The expression enrichment patterns of implicated genes may suggest the underlying biological mechanisms linking to specific brain regions.

### Genomic path analyses for SES indices

To dissect the genetic basis underlying the interplay among job attainment and other SES indices, we first examined the correlations between job attainment and educational attainment or income. The phenotypic correlations observed in the UK Biobank data were small or moderate (|*r*| ranged from 0.04 to 0.39; Supplementary Table S11). In contrast, the genetic correlations were moderate to large (|*r*_*g*_| ranged from 0.37 to 0.92). Among them, physical demands exhibited correlations in the opposite direction with educational attainment (*r*_*g*_ = − 0.91) and income (*r*_*g*_ = − 0.79). Emotional demands displayed positive moderate correlations with education (*r*_*g*_ = 0.37) and income (*r*_*g*_ = 0.44).

**Figure 3:**
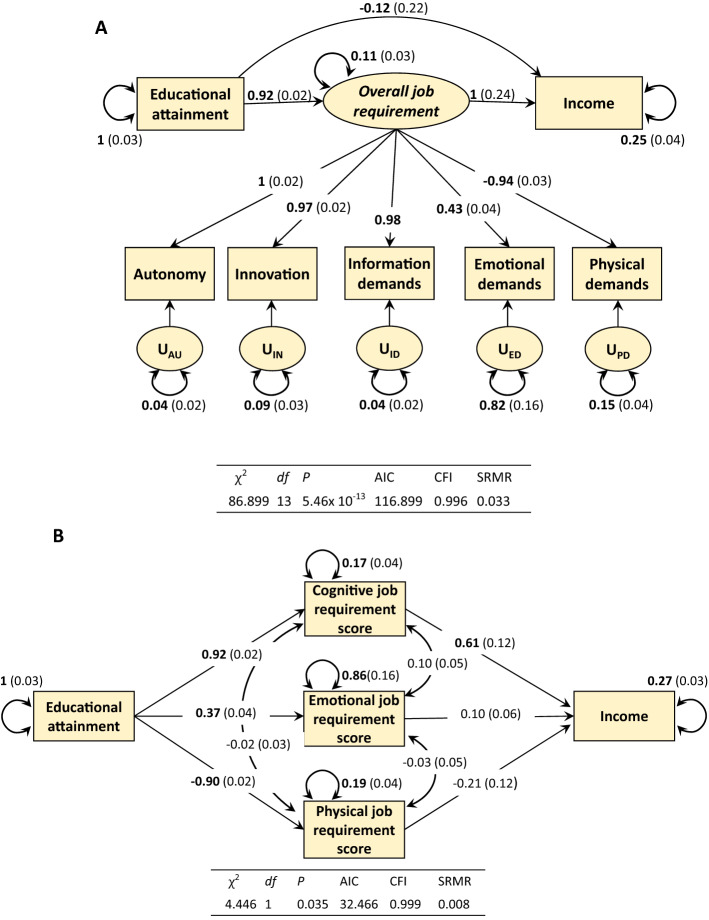
Analyses on the role of latent factors of job attainment genetically mediating educational attainment and income. (**A**) Fitting Genomic Structure Equation Modelling (Genomic SEM). Educational attainment, job attainment, and income are observed variables based on GWAS summary statistics. Overall job requirement is a latent (unobserved) variable. Beyond the path on overall job requirement , a direct path from educational attainment and income was not significant. As the model did not converge by including all six job characteristics simultaneously, we excluded complexity since its composite nature and high correlations with other traits. (**B**) A two-step factor score path analysis. The scores of three factors to present cognitive, emotional, and physical job requirement from exploratory factor analysis for job attainment, were calculated. The income was regressed on educational attainment and factors scores. Standardized beta coefficients are presented, as well as the test statistics for the modelling.

We performed the Genomic Structure Equation Modelling (Genomic SEM)^37^, including a latent factor of overall job requirement, the shared genetic variation across all job attainment traits, as illustrated from the previous EFA analysis. The model fit the data well (${\chi }^{2}$ = 86.899, *P* = 5.46 × 10^–13^, AIC = 116.899, CFI = 0.996, SRMR = 0.033). Figure 3a displays significant positive relationships from educational attainment to the latent overall job requirement factor, and from the latent overall job requirement factor to income. The mediated genetic effect from educational attainment to income through the latent overall job requirement factor represented by the multiplication of the above two-path coefficients was estimated at 0.92 (Sobel test statistic = 4.15, *P* < 0.001). The direct effect was not significant after accounting for the meditating effects. Removing a direct effect from education to income did not change conclusions (Supplementary Fig. S6a). To evaluate the content of this overall job requirement, we performed two-factor genomic SEM models by adding the second factor of emotional job requirement or physical job requirement separately (Supplementary Fig. S6). No additional mediating effects were identified, and the mediating effects of the latent overall job requirement factor remained significant. Thus, we conclude that the cognitive component of the overall job requirement factor played a major role in mediating genetic effects related to education on income.

As a robustness check, we also conducted a two-step factor score path analysis in Fig. 3b ^38^. In the first step, we calculated factor scores for cognitive, emotional, and physical requirements separately, based on the regression predictor results from the genomic EFA for job attainment. In the second step, we conducted a path analysis for these factor scores, mediating the genetic relationship between educational attainment and income. The path model fit the data well (${\chi }^{2}$ = 4.47, df = 1, *P* = 0.035, AIC = 32.47; CFI = 0.999, SRMR = 0.008). The mediated genetic effect through cognitive job requirement score variable was estimated at 0.56 (Sobel test statistic = 5.05, *P* < 0.001). Although there was a significant path between educational attainment and emotional or physical job requirement scores (β = 0.37, SE = 0.04; β = − 0.90, SE = 0.02, respectively), no significant effect on income was observed. Thus, neither of these two latent requirements exhibited significant mediating effect (Sobel test statistic = 1.64, *P* = 0.10; Sobel test statistic = 1.75, *P* = 0.08 respectively).

### Genetic correlations with well-being outcomes

**Figure 4:**
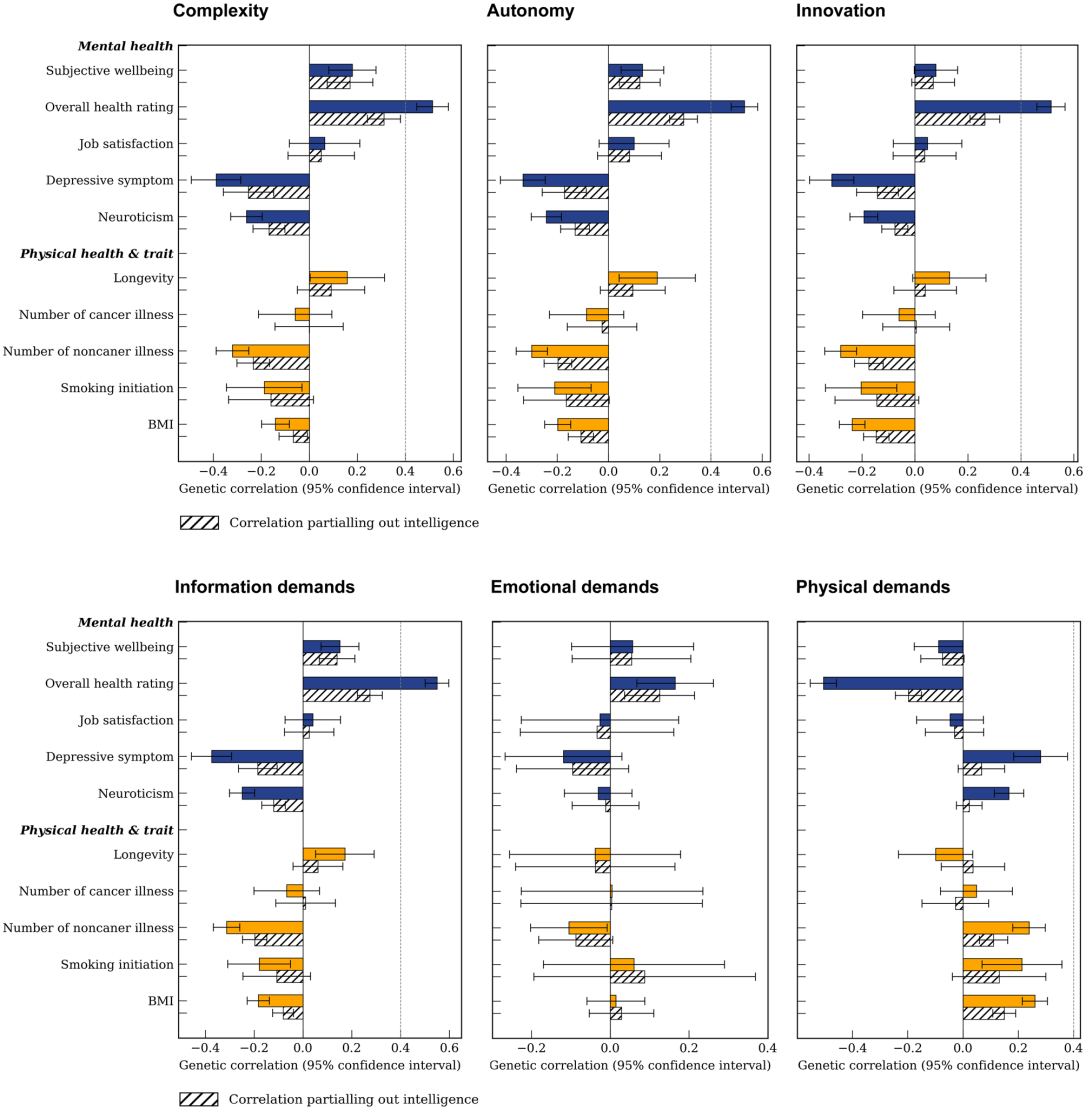
Genetic correlations of six job attainment characteristics with well-being. The x axis represents genetic correlation coefficients, and y axis labels each well-being trait. Coloured bars stand for genetic correlations before partialling out the genetic variance of intelligence, and grey shaded bars after partialling out the intelligence. Horizontal bars represent 95% CIs.

Next, we assessed genetic correlations between job attainment and well-being variables using summary statistics from either the large-scale GWAS or UK Biobank GWAS in European populations (see “Methods”). We found significant genetic correlations (false discovery rate FDR < 0.05) between complexity, autonomy, innovation, and information demands with positive well-being variables such as subjective well-being, overall health rating, depression symptom, neuroticism, longevity, number of non-cancer illnesses, BMI and smoking initiation (|*r*_*g*_| 0.13–0.55; Fig. 4 and Supplementary Table S12). The patterns were similar for educational attainments or income (Supplementary Table S13).

Resutls for physical and emotional demands, however, showed different patterns. Physical demands exhibited significant genetic correlations with the above health outcomes (except for subjective well-being and longevity) but in the opposite direction, i.e., lower health status ((|*r*_*g*_| 0.14 to 0.51). In contrast, emotional demands exhibited a significant genetic correlation with only the overall health rating variable (*r*_*g*_ = 0.17). For all the six job attainment characteristics, no significant correlation was observed between job satisfaction and the number of cancer illnesses.

Because intelligence was reported to be associated with various well-being variables^39–41^, and it was also observed to be genetically correlated with job attainment traits in our study (*r*_*g*_ ranged from 0.52 to 0.78). We partialled out the genetic variance of intelligence, in evaluating the genetic correlations between job attainment characteristics and well-being. After partialling out intelligence, the correlations for complexity, autonomy, innovation, and information demands with a majority of well-being outcomes remained significant, but with attenuated magnitudes, such as overall health rating (*r*_*g*_ 0.26–0.31), number of noncancer illnesses (*r*_*g*_ − 0.23 to − 0.17), and BMI (*r*_*g*_ − 0.15 to − 0.07). For physical demands, genetic correlations with three health outcomes remained significant: overall health rating (*r*_*g*_ = − 0.20), number of noncancer illness (*r*_*g*_ = 0.11), and BMI (*r*_*g*_ = 0.15). For emotional demands, the genetic correlation with overall health rating remained significant (*r*_*g*_ = 0.13).

## Discussion

Bridging the O*NET job characteristics information with various genomic datasets, we conducted whole-genome analyses of six job attainment characteristics and post-GWAS analyses. The genetic link between educational attainment and income was explained to a large extent by the latent cognitive requirement of job attainment characteristics, rather than the latent emotional and physical job requirements. The job attainment characteristics were genetically associated with positive well-being variables examined in this study, which is consistent with findings in previous research on educational achievementand income. Nevertheless, findings for physical demands showed a significant opposite direction and those for emotional demands exhibited only a correlation with the overall well-being variable. After partialling out intelligence-associated genetic variance, the magnitude of genetic correlations attenuated but most remained significant. Integrating job attainment phenotypes into the genomic SES and occupation health studies enables us to expand the research beyond the crude measures of socioeconomic attainment and better understand the interplay between genetics, different SES aspects, and well-being.

The current study contributed to the emerging field of social and organizational genomics. Our study demonstrated that O*NET is useful to extract objective and numerical occupational information for genomic study; the SNP-heritability estimates of job attainment characteristics were significant and similar in size to those of other traits such as risk tolerance, education, and household income^7,8,27,42,43^. These findings corroborate with a previous study based on a national U.S. twin sample which showed sizable genetic influences on job demands, autonomy, and job complexity^12^. There have been GWAS studies on occupation-related phenotypes, such as entrepreneurship^44^ and self-employment^45^, but unsuccessful in discovering any novel genome-wide loci partially due to the small sample sizes of these studies. With a much larger sample size employed in our study, we were able to identify novel genetic markers associated with job attainment. Furthermore, the multidimensional measurements of job attainment adopted in our study went beyond the often used occupation categories or ranking^4,6^ to allow for more nuanced examination of occupational traits. Intriguingly, these six job attainment characteristics were genetically manifested by three latent factors of cognitive, emotional, and physical job requirements.

Our results on pleiotropic relationships among SES variables indicate that the latent cognitive job requiement of job characteristics was the major underlying genetic basis linking education attainment and income. It has been well documented in the literature that, phenotypically, intelligence plays an important role in an individual’s educational attainment, occupational status, and income^46–51^. However, the stronger genetic correlations with other SES variables than those with intelligence suggest that additional mechanisms, such as personality and motivation^52,53^, may also be involved. Integrating job attainment characteristics into the genomic SES studies can help better understand the interplay between genetics and different social status aspects.

Emotional demands displayed distinctive patterns of findings compared to other job attainment characteristics. Among the six job characteristics, emotional demands seem to be much less related to cognitive ability, which is consistent with previous research^55,56^. Its relationships with education and income were significant but much weaker compared to other job characteristics. Jobs that require high emotional demands typically do not always require high education and are not necessarily always compensated for in the labor market^14,32,54^. The finding that emotional demands had the lowest heritability estimation among the six job characteristics is in line with the relatively smaller heritability of emotional intelligence estimated to be 0.35–0.45^55,56^, in comparison to the estimate of heritability for intelligence at around 0.80 in later adulthood^57^. In the current study, we also found little overlapping genome-wide significant SNP between emotional demands and other job characteristics. The findings suggest possible unique biological pathways influencing emotional job requirements and their relationships with educational attainment and income.

On the other hand, physical demands held negative genetic correlations with other job characteristics. It also had strong negative genetic correlations with intelligence, education attainment, and income. Besides shared cognitive job requirement, there was no independent path mediated by physical demands from educational attainment and income. The physical demands measure includes items covering both manual work activities and risk or unpleasant work conditions. Our results suggest that the often-reported negative relationship between health hazards in the workplace and the well-being of workers might be more complex to explain than just treating hazards as extrinsic factors. Occupation selection may play a role in explaining genetic iinfluences on occupational hazards. Future studies may explore possible person-environmental interactions concerining occupational hazards and well-being.

Genetic correlations between job characteristics and well-being revealed a consistent pattern: job complexity, control, information processing and low physical demands weregenetically associated with positive health outcomes; emotional demands had genetically less salient relationships with health indices compared to other five job attainment characteristics. Such findings, consistent with the previous twins’ study^12^, offer evidence that the relationships between job attainment characteristics and health variables are partially genetic. Such findings challenge the dominant assumption in research on job design and occupational health that the relationships between job/occupational status and well-being are primarily environmental in nature. They suggest that selection (e.g., self-selection and occupational selection) may also play a role. A possible practical implication pertains to occupational selection. When people select their occupations based on their interests and capabilities (which are partially shaped by the genetic lottery), they should be also aware of the potential health and well-being implications related to such choices made by themselves. From a public health perspective, when the society sorts its workforce into different occupations, it should also provide sufficient protective resources and related policies to prevent undesirable health and well-being consequences brought about by job attainment characteristics.

Within each job attainment characteristic, the genetic correlations between sex were moderate to high (mostly r_g_ > 0.76). From phenotypic analysis, although it is documented that males and females are different in their occupational preferences^58^ and their occupational differences partially explain their income gaps^59^. Our genetic correlation analyses provide limited evidence for substantial sex-differentiated genetic architecture within traits. However, at the SNP level, the heterogeneity of genetic effects emerged at several loci showing sex-specific effects. In particular, more than half of the index SNPs centered on physical demands, exhibiting stronger effects in men than in women. Interestingly, one of the strongest signals for men is at locus harbouring genes *UBE2K* and *SMIM14*, which were reported to be associated with sex hormone-binding globulin levels^60^. Sex-varying effects for physical demands could also be related to sex-specific body composition or metabolic levels^61^. The underlying mechanisms for these loci and these sex differences remain to be explored.

The career literature in organization research suggests that an individual’s career can be divided into different stages, such as the early career stage (focusing on trial and exploration), mid-career stage (focusing on establishment and stabilization), and late career stage (focusing on maintenance)^62,63^. Expectations, requirements, and achievements can vary in these different stages. However, we found no significant genetic difference between who were less than 55 and those who were more than 55 in the UKB data in each of those six job traits. One limitation of the UKB sample is that participants were senior citizens in their mid to late career stages. The restriction of age range and the retrospective design may have limited the ability to detect age effects in the UKB data. Future studies should use panel datasets with prospective designs to examine possible varying genetic effects across early, mid, and late career stages of individuals.

Genetic associations between job attainment and well-being were attenuated when partialling out intelligence-associated genetic variance, which provided additional evidence to support the relationship between intelligence and well-being at the genomic level^29,39,64,65^. Nevertheless, a substantial number of pairwise genetic correlations remained significant after controlling the genetic effects of intelligence, which indicates that job attainment features are genetically associated with health outcomes through mechanisms other than intelligence. It is theorized and empirically examined that demand and control, effort-reward imbalance, and organizational justice that are embedded in the work contexts can explain the relationship between work stress and health outcomes^10,66^. Future genomic studies can examine, after partialling out the effect of intelligence, whether any unique biological mechanisms are underneath the above mechanisms.

Future research should also examine genetic influences and correlations of job attainment from a dynamic perspective. Throughout the life course, many people will experience occupational transitions, suggesting dynamic career mobility patterns^24,49,67^. Information on occupational transitions and career history can be incorporated into the genomic investigation to bring deeper insights on dynamics of work and occupation.

Our study demonstrates that the UK Biobank dataset is a valuable source for occupation-related explorations. However, Participants of the UK Biobank volunteered to join in the study and the sample was not a national representative one. By design, only senior citizens were included. UK Biobank participants tended to be more affluent, better educated, and have better health conditions than the general population^68^. Furthermore, our analyses only included those with European ancestry. The above restrictions suggest it should be cautious to generate findings to the general population of different age groups or with different ethnical backgrounds.

Our findings should not be interpreted to advocate genetic determinism. Genetic variants cannot magically affect job attainments, income, or well-being. Influences of the genetic lottery are often carried out through various processes of selection (e.g., self selection or occupational selection). Further more, genetic influenes are mediated and are also often moderated by environmental factors. We encourage future research to explore such more nuanced processes and pathways during which environmental factors may moderate or mediate genetic influences on job attainment characteristics.

In conclusion, through whole-genome analyses, the current study adds to emerging evidence of the underlying shared and distinctive genetic architecture for the six major job attainment characteristics, significant genetic correlations with well-being, as well as underlying genetic links with other SES variables. Integrating well-defined job attainment characteristics into SES and occupational genomic studies shed light on a more complete understanding of the genetic basis for various SES variables, and their interplay with well-being.

## Materials and methods

### Phenotype measurement and definitions

UK Biobank cohort is a population-based cohort study in the United Kingdom, which involves above 500,000 participants aged 40 years or older during their recruitment between 2006 to 2010^69,70^. In the UK Biobank data, participants’ jobs were coded using the four-digit UK Standard Occupational Code (SOC) 2000 (Field ID 132) based on detailed job information (e.g., job tasks and activities) collected during participants’ baseline visits to assessment centers for the discovery sample. We built a procedure to link the UK SOC 2000 to the U.S. SOC 2000, which allowed us to derive occupational characteristic information from the U.S. Department of Labor’s Occupational Information Network (O*NET). O*NET is based on the U.S. SOC system and includes hundreds of job characteristic measures for over 1100 occupations, sufficiently representing the national labor force in the United States, and has been used in many other countries via similar occupational classification systems^71,72^. First, we converted UK SOC 2000 codes into U.S. SOC/O*NET job titles using Computer Assisted Structured Coding Tool (CASCOT)^73^. Second, we manually checked and matched occupations in these two systems based on detailed job descriptions (e.g., job tasks and activities), based on the International Labor Organization’s International Standard Classification of Occupations (ISCO) to ensure accuracy and reliability of the crosswalk in the first step. For the 502,538 participants, we were able to match O*NET job titles for 274,223 individuals. The remaining 192,010 individuals who did not provide SOC codes and 36,305 who had SOC 2000 codes but no sufficient information to match the O*NET job titles were excluded.

The study involved six job attainment phenotypes, including complexity, autonomy, innovation, and three types of job demands (information demands, emotional demands, and physical demands; Box 1), with a detailed description of items for each phenotype in Table S1. Complexity was evaluated with an overall composite index of levels of abilities (52 items of specific abilities required in a job), skills (35 items), and knowledge (33 items)^16^. Sample items are: “What level of oral expression is needed to perform your current job?”, “What level of complex problem solving is needed to perform your current job?” and “What level of economics and accounting knowledge is needed to perform your current job?”. All the items used a 7-point scale (1 = lowest level, 7 = highest level). The Cronbach’s α was 0.98.

Autonomy was assessed with the same approach used previously^13,74^, using 2 items: “How much freedom do you have to determine the tasks, priorities, or goals of your current job” and “How much freedom do you have to make decisions without supervision?”. Both items used a 5-point scale (1 = no freedom, 5 = a lot of freedom). The Cronbach’s α was 0.90. Based on the prior study on O*NET creativity requirements^75^, we assessed innovation with 1 item: “How important is innovation to the performance of your current job?” (1 = not important, 5 = extremely important). This item captures the definitions of creativity: the production of novel and useful ideas^76^.

Job demands were measured by adapted scales^14^. We assessed information demands with 17 items. A sample item is “What level of processing information is needed to perform your current job?”. Five items were used to measure emotional demands, with a sample item as “How often is dealing with unpleasant, angry, or discourteous people a part of your current job?”. Physical demands were assessed with 12 items. A sample item is “In your current job, how often are you exposed to sounds and noise levels that are distracting and uncomfortable?”. All items used a seven-point scale indicating the levels of the demands. The Cronbach’s α were 0.96, 0.80, and 0.96 for the 3 sub-scales respectively.

### GWAS data analyses for the UK Biobank study

We used imputation genotypes released by UK Biobank in March 2018, which includes the full set of genotypes imputed on the Haplotype Reference Consortium (HRC) and UK 10 K haplotype resource. The quality control and imputation were done by UK Biobank and have been described elsewhere^69^ (Supplementary Note). Our analysis included participants of European ancestry. Variants with MAF < 0.1% and with IMPUTE info < 0.3 were removed. A total of 19,400,443 imputed or genotyped variants in 408,344 individuals passed the QC procedure. We then matched job characteristic phenotypes with genotype data, yielding 219,483 individuals in the GWAS analyses.

We assumed an additive genetic model where the dosage of each variant was a continuous variable ranging from 0 to 2 for the effect allele. For each study, a mixed linear model accounting for the family structure was conducted to determine the association between variants and job attainment phenotypes represented as a quantitative trait. The analysis was conducted with the software BOLT-LMM v2.3.2 (see URLs)^77^, a program specifically developed to analyze large-scale UK Biobank in a fast and efficient manner. The association analysis for each phenotype was adjusted for age, sex, genotyping array, and top 20 principal components.

Independent significant variants and their surrounding genomic loci were identified using LD-clumping in FUMA (see URLs). For single-trait GWAS, the lead variant is the one with the smallest association at the threshold of *P* < 5 × 10^–8^, independent from other variants at each locus (*r*^*2*^ < 0.01). A locus was defined by an index SNP with the region flanking 500 kb on both sides. The LD information was obtained from 10,000 unrelated individuals of European ancestry (UK Biobank project ID 16406). We further performed a conditional analysis to identify additional independent variants at each locus by adjusting for the lead variant using GCTA 1.92.4beta (see URLs)^78^. There were no additional loci further identified. For genome-wide significance for 6 phenotypes in the study, we applied the Bonferroni correction to control the family-wise error rate, i.e., a cutoff of *P* value of 5 × 10^–8^/6 = 8.33 × 10^–9^.

The coordinates and variant identifiers were reported on the NCBI B37 (hg19) genome build. The functional annotation and gene mapping were performed using ANNOVAR (v.2018Apr16), including types of intronic, exonic, intergenic, 5′-UTR, and 3′-UTR, etc.^79^. The regional plot was drawn for each identified locus using LocusZoom (see URLs) and used the LD estimates from the 1000 Genomes European samples.

In addition, we also conducted GWAS analyses by gender for the six job characteristics with 110,794 female and 108,689 male participants who are Caucasians (Field ID 22006). We used the linear mixed model to account for the genetic relatedness between individuals as implemented by BOLT-LMM and adjusted on up to 20 principal components for population stratification in the Caucasian population. Age and genotype measurement batch were also included as covariates.

For those top variants identified, we assessed genetic effects across age subgroups. We performed association analyses for individuals at 55 years old or below (Age ≤ 55, N = 118,024), and those over 55 (Age > 55; N = 111,059), separately. For subgroup analysis, we applied the linear mixed model adjusted for age (to account for the residual effects), sex, genotype measurement batches, and 20 principal components using BOLT-LMM. Two-sided *P* values on Z-score were reported to compare genetic effects between the two age groups.

### Independent samples for replication and validation

We evaluated the implicated lead variants and Polygenic scores (PGSs) in a UK Biobank follow-up cohort of European participants (N = 30,837) and the U.S. Add Health Wave V study of European individuals^80^ (N = 3,817; Supplementary Figs. S7, S8 and Supplementary Table S14). The UK Biobank follow-up sample had valid job information in the follow-up survey, but not in the discovery phase because baseline job information was not available for them. The detailed information for genetic association analysis in the replication datasets was presented in the Supplementary Note.

### Polygenic Score (PGS) analysis

PGS evaluates the cumulative effects of thousands of genetic variants identified from GWAS, including many with small effects. We generated PGSs for the 6 job attainment characteristics in both UK Biobank follow-up cohorts and Add Health using PRsice 2 (see URLs). PGS was a summation of the number of risk alleles weighted by their effect size estimated from the discovery of UK Biobank GWAS data. Genotype data of the same testing samples were used as a reference panel for LD calculation. PRSice 2 tested each PGS calculated from the independent variants (r^2^ ≥ 0.01) passing a series of *P* value thresholds from 1 × 10^–5^ to 1. The optimal model fitting with the most predictive empirical *P* value was evaluated using the R^2^ coefficients^81^. For each *P* value threshold, two multivariate linear regression models were constructed. One included PGS and adjusted for age, gender, genotyping array, and top principal components as covariates; another only included covariates. The difference between the R^2^ coefficients from the two regression models represents the proportion of phenotypic variation explained by the PGS.

### Pathway analyses of implicated genes

For the implicated genes identified, we investigated functional annotation using g:Profiler (see URLs). We used “g:GOST” function to perform pathway analysis on identified associated genes. Pre-specified pathways include Gene Ontology, pathways from KEGG, Reactome, WikiPathways, and protein complexes from CORUM^82^. The significant pathway was claimed at the adjusted *P* value < 0.05 after correction for multiple testing in the analyses.

### Gene enrichment analyses in GTEx tissues

We tested for expression enrichment from the gene-set analysis in 54 tissue types based on GTEx RNA-seq data (V7; see URLs) using FUMA^34^ (see URLs). Gene expression values (reads per kilobase per million, RPKM) were normalized by log2(RPKM + 1) transformation per tissue type. Differently expressed genes were pre-calculated for the average expression of genes compared to others in each tissue type using a two-sided t-test on GTEx RNA-seq data. Input genes from the gene-set analysis were tested against each of the differently expressed gene sets using the hypergeometric test. Gene enrichment analysis was performed in each tissue type separately. Significant enrichment was claimed at *P* value < 0.05/54 = 9.26 × 10^–4^.

### SNP-heritability and genetic correlation analysis

We applied the univariate LDSC method using the software LDSC v1.0.1 (see URLs) with GWAS summary statistics to estimate SNP-*h*^2^^25^. Of 19,400,443 SNVs from GWAS summary-level data for job attainment traits, we included SNPs presented in the HapMap3 European panel, with the exclusion of the major histocompatibility complex (MHC) region on chromosome 6. SNPs with INFO ≤ 0.9 and MAF ≤ 0.01 were further removed, resulting in 1,174,163 SNPs for the LDSC regression analyses. We computed genetic correlation coefficients between each pair of job attainment traits based on GWAS summary statistics, using the bivariate LDSC method, by regressing the product of testing statistics (z statistics) from each phenotype against the LD scores^30^.

### Genetic correlation between-sex and sex-differentiated genetic effects

Using summary statistics of GWAS conducted for each phenotype stratified by sex, we tested whether the genetic correlations were significantly different than 1 between sex. Within each trait, we used LDSC bivariate regression to estimate genetic correlations (*r*_*g*_) between sex, and tested sex differences in genetic correlations. To identify the genetic variants showing the differences in genetic effects by sex for each trait, we computed z-scores from sex-stratified GWAS regression coefficients and identified genetic variants with sex-differentiated effects. Z-score statistics= ${(Beta}_{female}-{Beta}_{male})/\sqrt{{SE}_{female}^{2}+{SE}_{male}^{2}}$ were calculated to test the differences of association effect between genders for a specific genetic variant. We used Bonferroni corrected threshold to select top loci showing between-sex genetic effect differences.

### Exploratory factor analysis (EFA)

We performed genetic exploratory factor analyses (EFA)^33^ to uncover the underlying genetic structure of a set of variables and clusters correlated traits using the Genomic Genomic Structural Equation Model (Genomic SEM)^37^. The genetic covariance structure was generated using LDSC extended method. Cumulative genetic variance explained by factors was used to determine the number of clusters and standardized factor loading of each trait for a factor. The R package “GenomicSEM” was used in the analysis (see URLs).

### Genetic correlations with well-being

We assessed genetic correlations between the job attainment characteristics and 11 well-being variables and 2 SES indices (i.e., educational attainment and income) using summary statistics either from UK Biobank data or other GWAS of European ancestry. Briefly, we included subjective well-being^83^, overall health rating^84^, job satisfaction (UK Biobank data field 4537), depressive symptom^83^, neuroticism^85^, longevity^86^, number of cancer illnesses (UK Biobank data field 134), number of noncancer illness (UK Biobank data field 135), smoking initiation^87^, BMI^88^, and intelligence^29^. The bivariate LDscore regression was applied for genetic correlation calculation^30^. Note that for the illustration purpose, in our results, the opposite scores of the originally subjective well-being and overall health rating were displayed so the higher score indicates a person has a high status of subjective well-being and overall health status. We included the SNPs present in the HapMap3 European panel, with the exclusion of the major histocompatibility complex (MHC) region on chromosome 6. SNPs with INFO ≤ 0.9 and MAF ≤ 0.01 were further removed. The bivariate LDSC regression was applied for genetic correlation between each pair of job attainment and other traits^30^. For each job attainment trait, Benjamin–Hochberg FDR^89^ was used to correct for multiple testing for the genetic correlations analysis.

To estimate the genetic correlations after partialling out the genetic variance of intelligence, we used the Genomic SEM^37^. For each pair of job attainment and another trait, we fit the Genomic SEM model including three traits: job attainment trait (X), well-being or SES-related phenotype (Y), and intelligence (Z). In the path diagram, there was a bidirectional arrow between two traits of X and Y, and directional arrows from Z to X and Z to Y. The genetic effect of Z was regressed out from the variance of X and Y affecting the heritability and genetic correlation. The genetic covariance matrix of X, Y, and Z was produced by LDSC method implemented in Genomic SEM. The same variant filtering procedure for calculation correlation before partialling out the genetic variance of intelligence was applied in the Genomic SEM analysis. The process was repeated for each well-being outcome.

### Genomic SEM and path analysis

We conducted Genomic SEM analyses examining the joint genetic paths among educational attainment, job attainment, and income^37^. GWAS summary statistics were used for education attainment^8^ and household income^90^. We explored several SEM models by including (1) one latent factor underlying job attainment, representing overall job requirement, (2) adding a direct effect from the education to income, (3) two-factor models by adding the second factor of emotional job requirement or physical job requirement separately. As the model did not converge by including all six job characteristics simultaneously, we excluded complexity since its composite nature and high correlations with other traits. Model fit was evaluated using Akaike Information Criteria (AIC), Comparative Fit Index (CFI) and the standardized root mean square residual (SRMR).

We used the Sobel test^91^ for the indirect effects from educational attainment to income through job attainment (see URLs).

To confirm the robustness of the results from genomic SEM, we also performed a two-step path analysis to compute composite scores for three latent factor scores based on the regression predictors estimated from the genomic EFA modeling for 6 job attainment characteristics (Table 2). The job requirement scores were calculated as follows:

$$Cognitive \; job \; requirment \; score=0.928\times Complexity+0.989\times Autonomy+0.953\times Innovation+0.971\times Information \; demand+0.534\times Emotional \; demand+0.885\times Physical \; demand$$

$$Emotional \; job \; requiremnt \; score=0.021\times Complexity +0.005\times Autonomy -0.187\times Innovation-0.129\times Information \; deamnd+0.840\times Emotional \; demand +0.181\times Physical \; demand$$

$$Physical \; job \; requiremnt \; score=0.371\times Complexity +0.013\times Autonomy -0.037\times Innovation+0.106\times Information \; deamnd-0.092\times Emotional \; demand +0.424\times Physical \; demand$$

We ran multivariable LD-Score regression to obtain the genetic covariance matrix (S) and corresponding sampling covariance matrix (V_S_). We estimated the regression coefficients for the paths between educational attainment, latent factor scores, and income. Model parameters were estimated using the Diagonally Weighted Least Square (DWLS) method implemented in Genomic SEM.

## Supplementary Information

Supplementary Information.

## Data Availability

The individual data that support the findings of this study are available from UK Biobank and Add Health, with the restrictions applied for public use.

## Code availability

Sources (references and URLs) of all algorithms and software used in the paper are provided in the paper.

O*NET: https://www.onetonline.org/, BOLT-LMM v2.3.2: https://data.broadinstitute.org/alkesgroup/BOLT-LMM/downloads/old/BOLT-LMM_v2.3.2.tar.gz, Plink v1.07: http://zzz.bwh.harvard.edu/plink/download.shtml#download, GCTA version 1.92.4beta: https://cnsgenomics.com/software/gcta/#Download, ANNOVAR(2018Apr16): https://doc-openbio.readthedocs.io/projects/annovar/en/latest/user-guide/download/, LocusZoom: http://locuszoom.org/, PLINK V2: https://www.cog-genomics.org/plink/2.0/, GCTA-fastBAT: https://cnsgenomics.com/software/gcta/bin/gcta_1.93.0beta.zip, LDSC: https://github.com/bulik/ldsc, PRSice 2: https://choishingwan.github.io/PRS-Tutorial/prsice/, GenomicSEM: https://github.com/MichelNivard/GenomicSEM, FUMA: https://fuma.ctglab.nl, g:profiler: https://biit.cs.ut.ee/gprofiler/gost, GTExPortal: https://gtexportal.org/home/, HaploReg: https://pubs.broadinstitute.org/mammals/haploreg/haploreg_v3.php, METAL: https://genome.sph.umich.edu/wiki/METAL, CASCOT: https://warwick.ac.uk/fac/soc/ier/software/cascot/, GitHub: https://github.com/MichelNivard/GenomicSEM, Sobel test: http://quantpsy.org/sobel/sobel.htm.
