# Supplementary material for: Genetic basis of job attainment characteristics and the genetic sharing with other SES indices and well-being
Source: Sci Rep. 2022 May 26;12:8902. doi: 10.1038/s41598-022-12905-y (PMC9135765; doi:10.1038/s41598-022-12905-y)
Supplement: Supplementary file 1 — Supplementary Information. [file 41598_2022_12905_MOESM1_ESM.docx]

**Genetic Basis of Job Attainment Characteristics and the Genetic Sharing with Other SES Indices and Well-Being**

Zhaoli Song, Wen-Dong Li, Hengtong Li, Xin Zhang, Nan Wang, Qiao Fan

**Supplementary Materials**

**Supplementary Notes**

1. Genotyping and imputation
2. Phenotyping in replication samples
3. Genetic association analysis in replication samples

**Supplementary Figures**

Figure S1. Flowchart of data processing and samples included in the study.

Figure S2. Quantile-quantile plots with genomic control inflation factor lambda λ_G_

Figure S3. Manhattan plots for six job attainment characteristics

Figure S4. Regional association plots for the 16 identified loci, LD (r^2^) shown in the plots based on European populations.

Figure S5. Quantile-quantile plots of Z-scores for sex-differentiated genetic effects.

Figure S6. Alternative Genomic SEM models.

Figure S7. Data preparation for the UK Biobank follow-up cohort.

Figure S8. Data preparation for the Add Health Wave V cohort

**Supplementary Tables**

Table S1. Job attainment characteristics from the O*NET database occupation code

Table S2. λ_GC_, LDSC-intercept and SNP heritability estimations for job attainment traits in the UK Biobank data

Table S3. Top loci identified for six job attainment characteristics from the UK Biobank GWAS

Table S4. Genetic effects of top variants in subgroups by age

Table S5. Replication and meta-analysis results in UK Biobank and Add Health datasets

Table S6. Between-sex genetic correlation for each job attainment phenotype

Table S7. Sex-specific SNPs with significant genetic differences for job attainment phenotypes

Table S8. Polygenic risk score associated with job attainment traits in testing samples

Table S9. Pathway anlaysis for top implicated genes using g: Profiler

Table S10. GTEX gene enrichment analysis using FUMA

Table S11. Phenotypic and genetic correlation between job attainment and SES indices

Table S12. Genetic correlation between job attainment and well-being, and correlation partialling out intelligence

Table S13. Genetic correlation between educational attainment, income and well-being.

Table S14. Summary of O*NET linked phenotype scores in the UK Biobank follow-up dataset and Add Health dataset

**Supplementary Notes**

**1. Genotyping and imputation**

UK Biobank

We used genotype data from the March 2018 release of imputed genetic data from the UK Biobank. Samples were genotyped on the two customized SNP arrays (the UK BiLEVE Axion array and the UK Biobank Axiom array. The quality control and imputation were done by UK Biobank (*1*). Briefly, genotyped variants were filtered based on batch effects, plate effects, departures from HWE, genotype platform, and discordance across control replicate. Participant samples were excluded based on missing rates larger than 5%, inconsistencies in reported versus genetic sex, and excessive heterozygosity based on a set of 605,876 high-quality autosomal markers. Genotypes were phased and the imputation was performed using IMPUTE4 with the Haplotype Reference Consortium (HRC) and UK10K dataset used as the reference set. We excluded genetic variants with MAF < 0.1%, and poorly imputed markers (IMPUTE info < 0.3), resulting in 19,400,443 autosomal variants imputed or genotyped on 408,344 individuals of European ancestry. Among them, 219,483 individuals were included for GWAS on job characteristics traits.

Between June to September 2015, more than 120,000 UK Biobank participants completed an online follow-up assessment on their employment history. Among them, the UK follow-up samples comprised of 30,387 individuals with genome data and valid job information of the last job, but not included in the discovery phase because of lacking baseline job information. For the UKB follow-up dataset, the genotyping, imputation, and filtering procedure similar to the one described above for the UKB discovery, resulting in 30,837 individuals of European ancestry.

Add Health cohort

The Add Health is a U.S. multi-wave longitudinal study of adolescents (*2*). We used the Add Health Wave V data that was conducted between 2016 to 2018. The imputed genotype data includes 9,947 individuals of multiple ancestries. The samples of multiple ancestries were genotyped using Illumina’s Human Omni1-Quad-BeadChip (N=9,947). In this study, analyses were limited to individuals of European-ancestry and cryptically related individuals, and ancestry outliers were dropped from analyses. Add Health study applied a stringent QC on genotyped data, variants with a per-variant missing call rate > 2%, MAF < 1%, and Hardy-Weinberg Equilibrium *P* < 1 × 10^-4^ were excluded (*3*). In the individual-level filters, only samples of genetically ascertained European ethnicity were ascertained through the protocol developed for the association analysis of the GWAS & Sequencing Consortium of Alcohol and Nicotine use (GSCAN). Individuals with per-sample missing call rate > 5% and excessively high or low heterozygosity were removed (F-statistics lower than -0.3 or higher than 0.3). Finally, an Identity-By-State (IBS) binomial test was conducted to filter out ancestral outliers using a threshold of 0.05 ([https://addhealth.cpc.unc.edu/wp-content/uploads/docs/ user_guides/](https://addhealth.cpc.unc.edu/wp-content/uploads/docs/%20user_guides/)). After imputing the genetic data to the Haplotype Reference Consortium panel (HRCr1.1) 2016 using the Michigan Imputation Server, only HapMap3 variants were included, which are well imputed and provide good coverage of common variation across the genome. PCA analyses were conducted in GCTA v.1.92.4beta (*4*). To remove poor quality variants and likely false-significant associations, we excluded variants at imputed quality R^2^ < 0.3 and MAF < 1%, which resulted in 6,049,177 autosomal genetic variants.

**2. Phenotypes in replication samples**

We linked the Add Health Wave V occupational codes to the O*NET database. All the phenotype measurements were the same as those used in the UK Biobank study. Among them, 3,817 individuals with six phenotypes information were available. The participants were on average 59.8 years old and 43.4% males in the UK Biobank follow-up dataset, and 37.8 years old and 43.9% males in the Add Health dataset. Distributions of job attainment scores were similar to those from the discovery UK Biobank data (**Table S10**).

**3. Genetic association analysis in replication samples**

UKB follow-up data

The association analysis was conducted using the linear mixed model to account for genetic relatedness, for six job characteristics respectively, in samples with BOLT-LMM, adjusting for age, sex, genotyping array, and the top 20 principal components of the genetic data. Variants with IMPUTE info <0.3 were removed from the analysis.

Add Health cohort

Linear regression analyses were conducted in unrelated samples for each phenotype respectively at a per-variant basis using Plink2, adjusting for age, gender, and the top 10 principal components Variants with IMPUTE info <0.3 were removed from the analysis.

**Supplementary Figures**


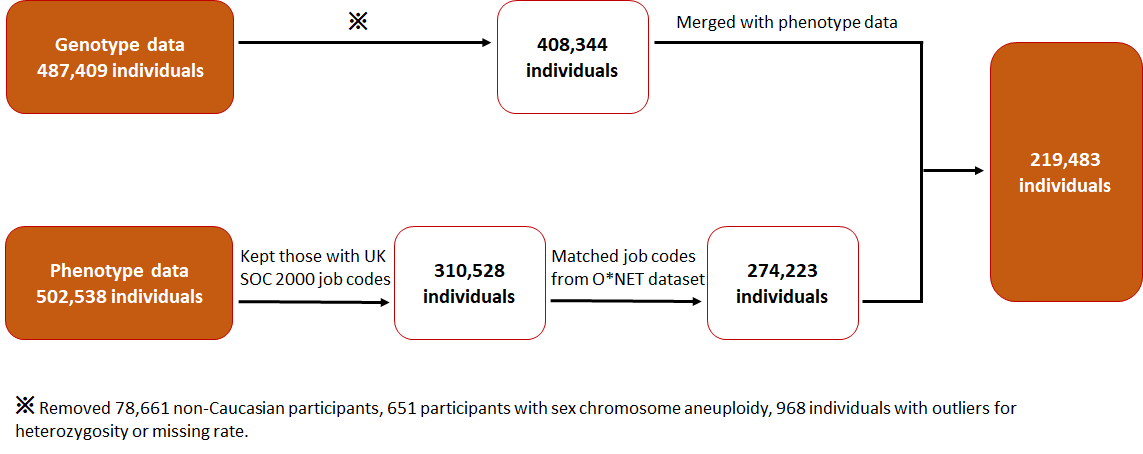


**Figure S1. Flowchart of data processing and samples included in the study.**

Included were 219,483 participants of European ancestry from the UK with both genotypic data and phenotypical measurements of job characteristics. For phenotype data, we linked the four-digit UK Standard Occupational Code (Field ID 132) in the UK Biobank data to the U.S. O*NET database occupation code and then acquired the quantified measurement of each job characteristic.


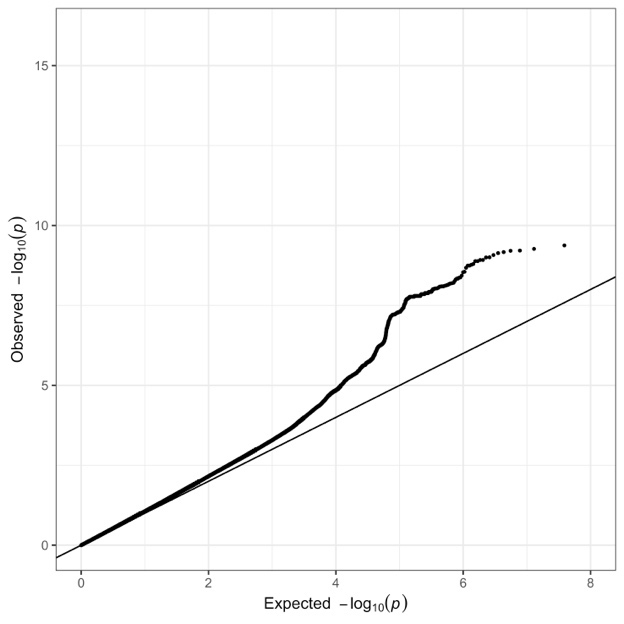

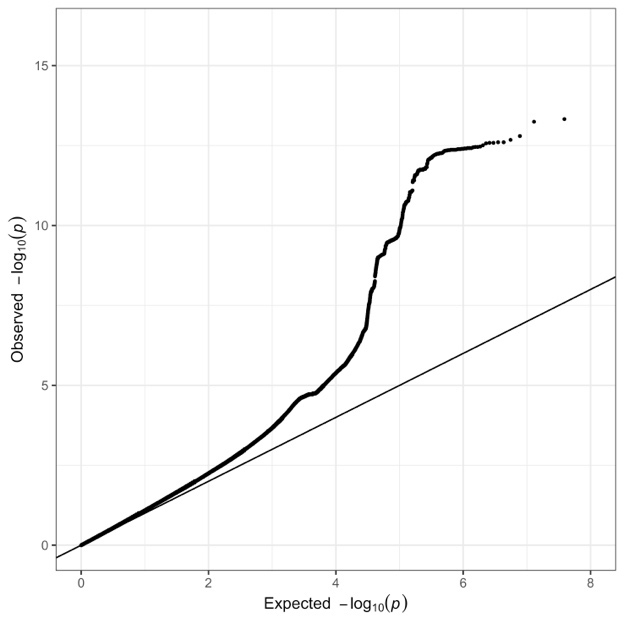


λ_G_=1.096

λ_G_=1.096

Complexity Autonomy


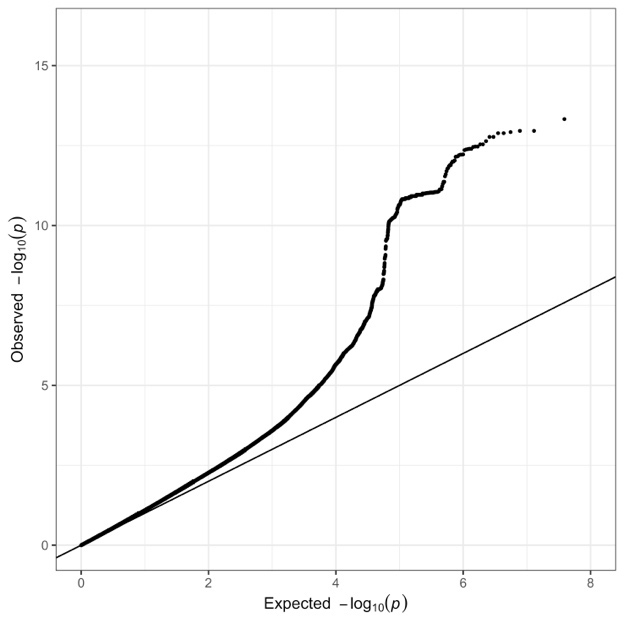

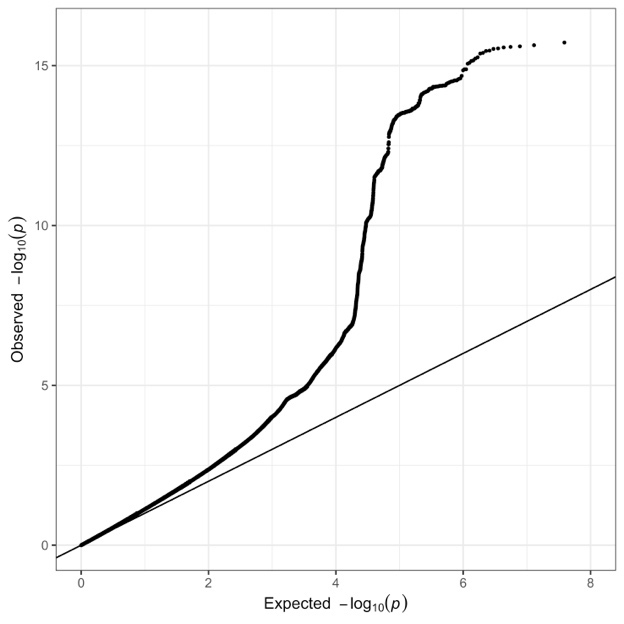


λ_G_=1.147

λ_G_=1.096

Innovation Information demands


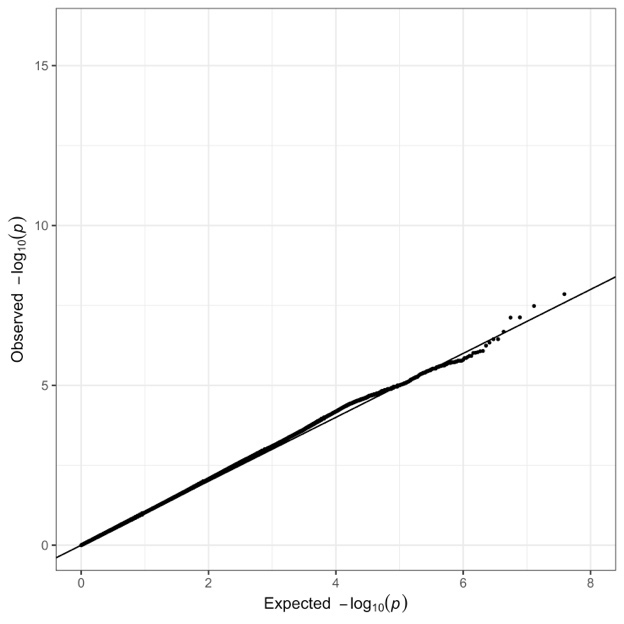

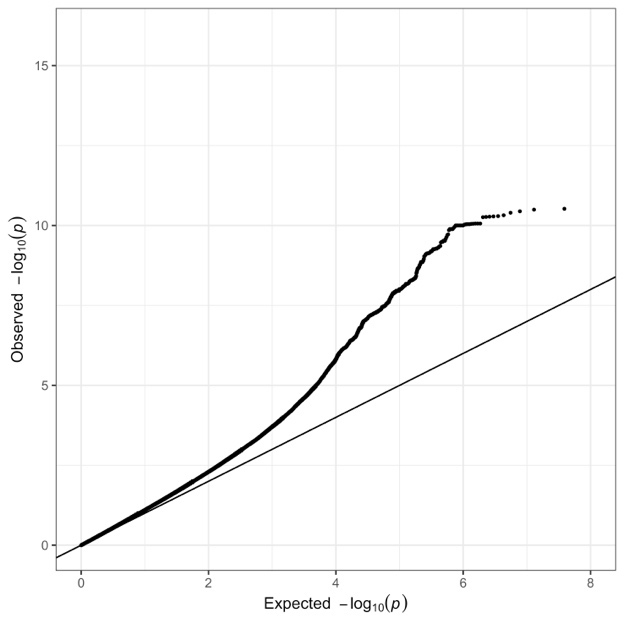


λ_G_=1.147

λ_G_=1.047

Emotional demands Physical demands

**Figure S2. Quantile-quantile plots with genomic control inflation factor lambda λ_G_**

**
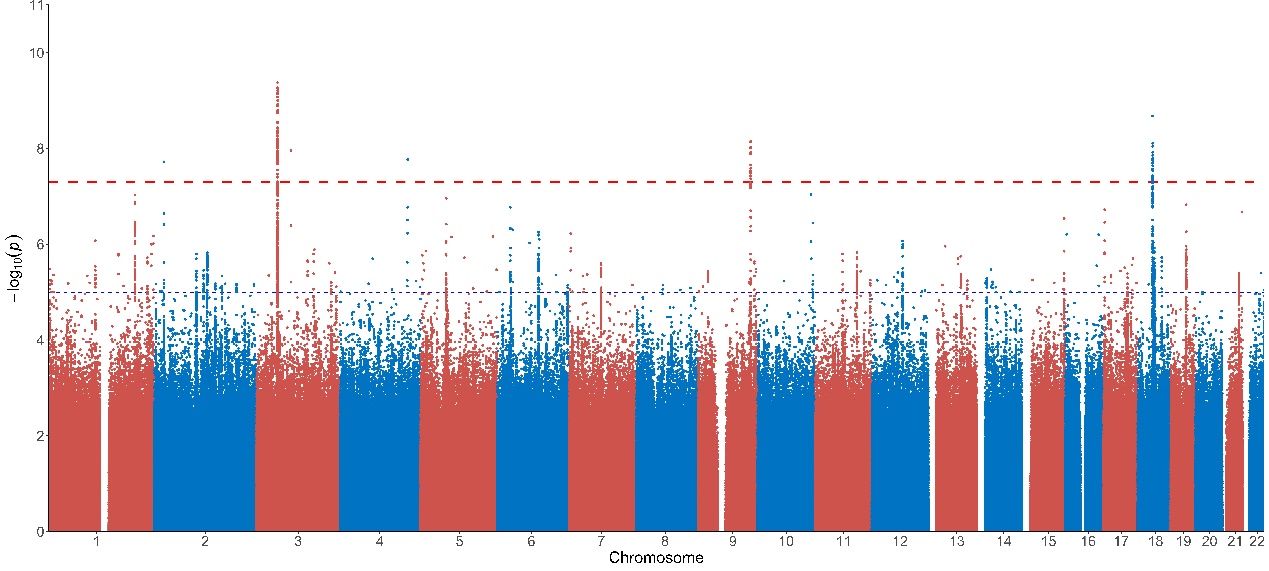
**

**Complexity**

**
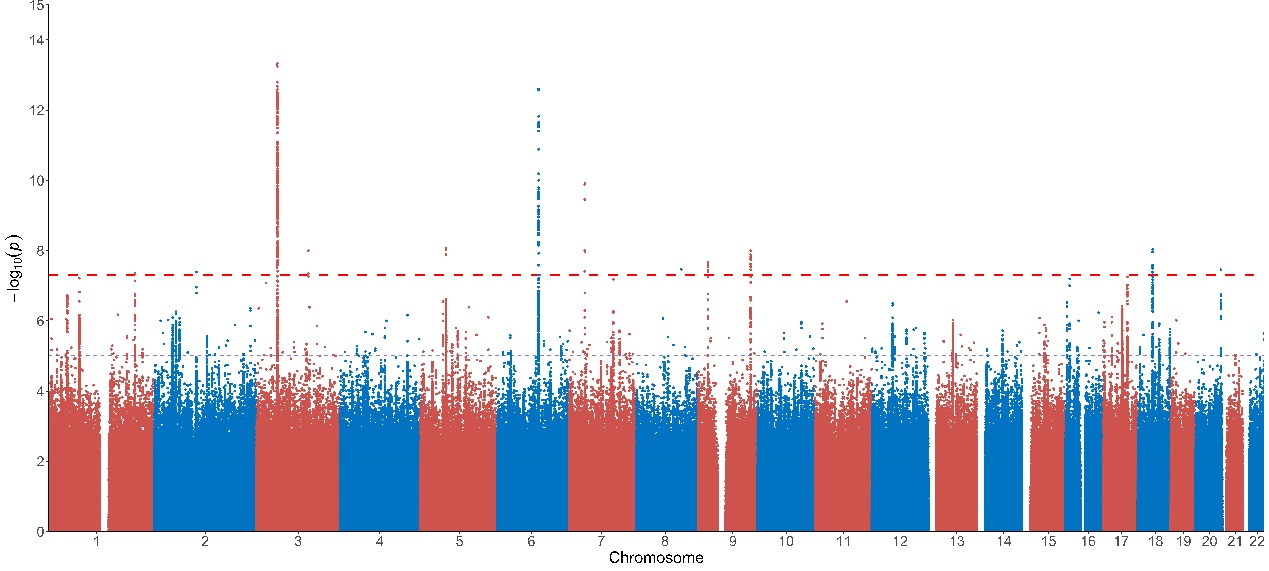
**

**Autonomy**

**
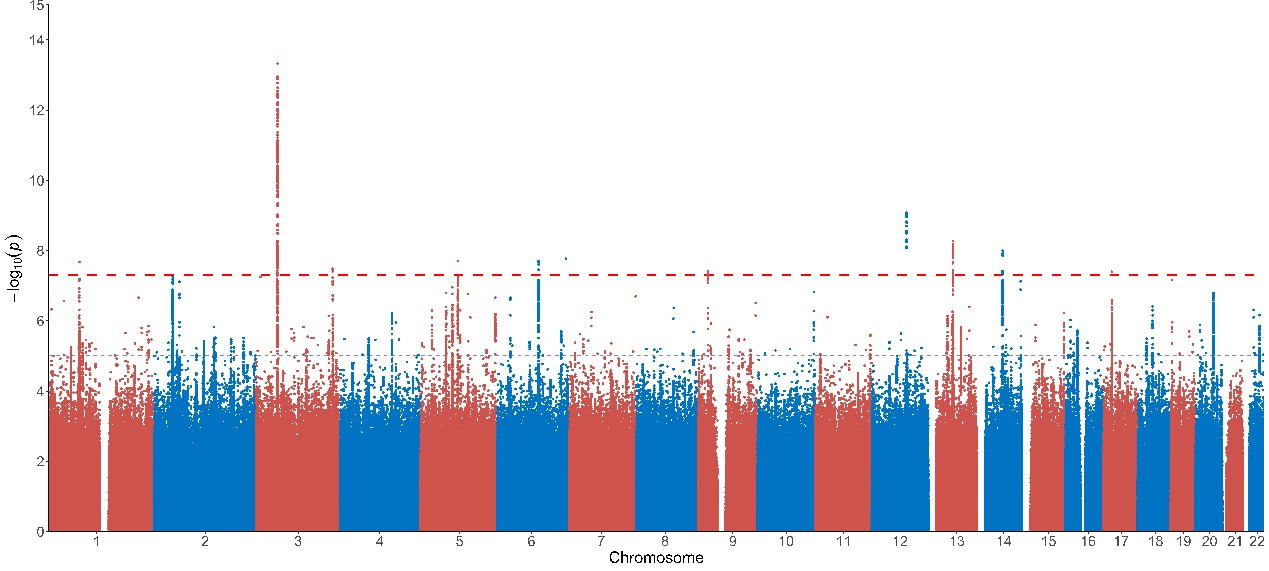
**

**Innovation**

**
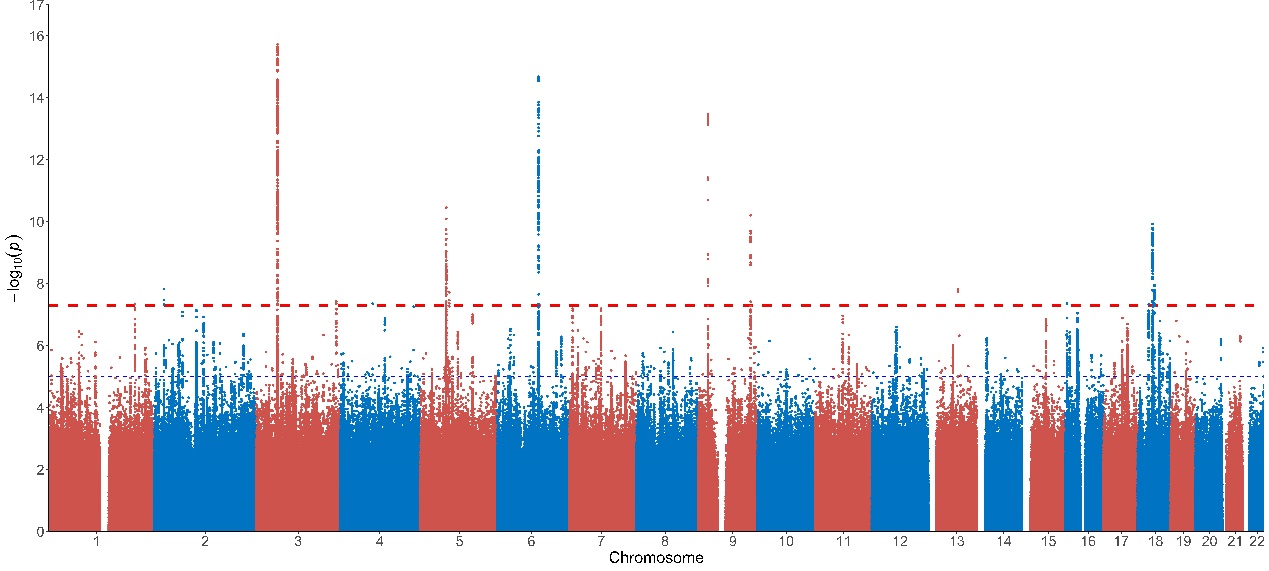
**

**Information demands**

**
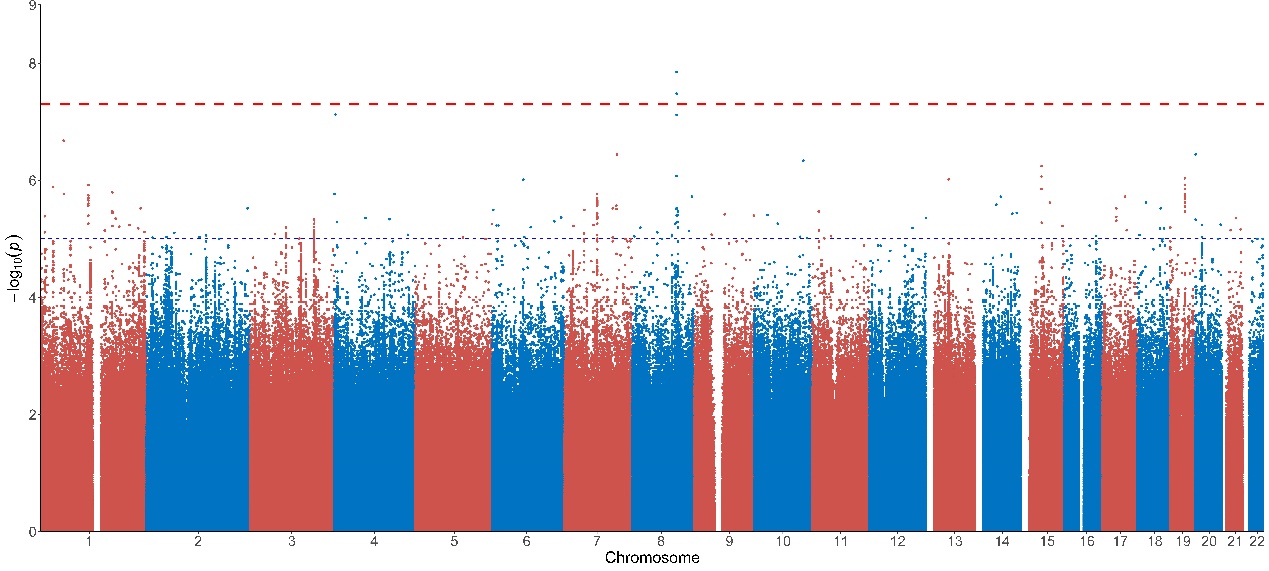
**

**Emotional demands**

**
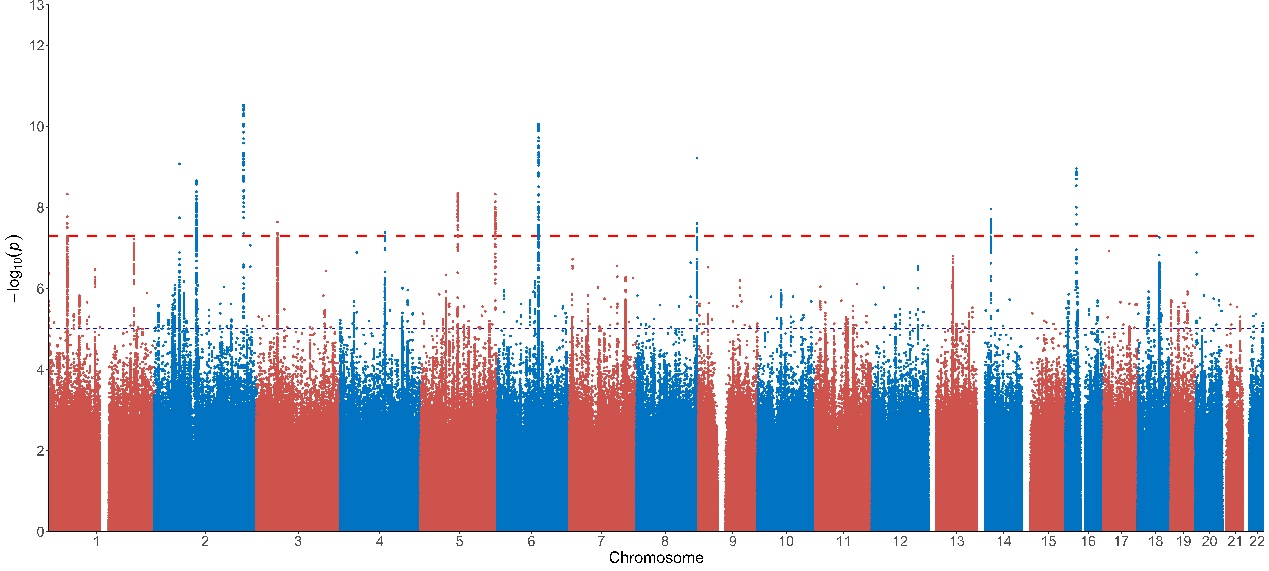
**

**Physical demands**

**Figure S3. Manhattan plots for six job attainment characteristics**


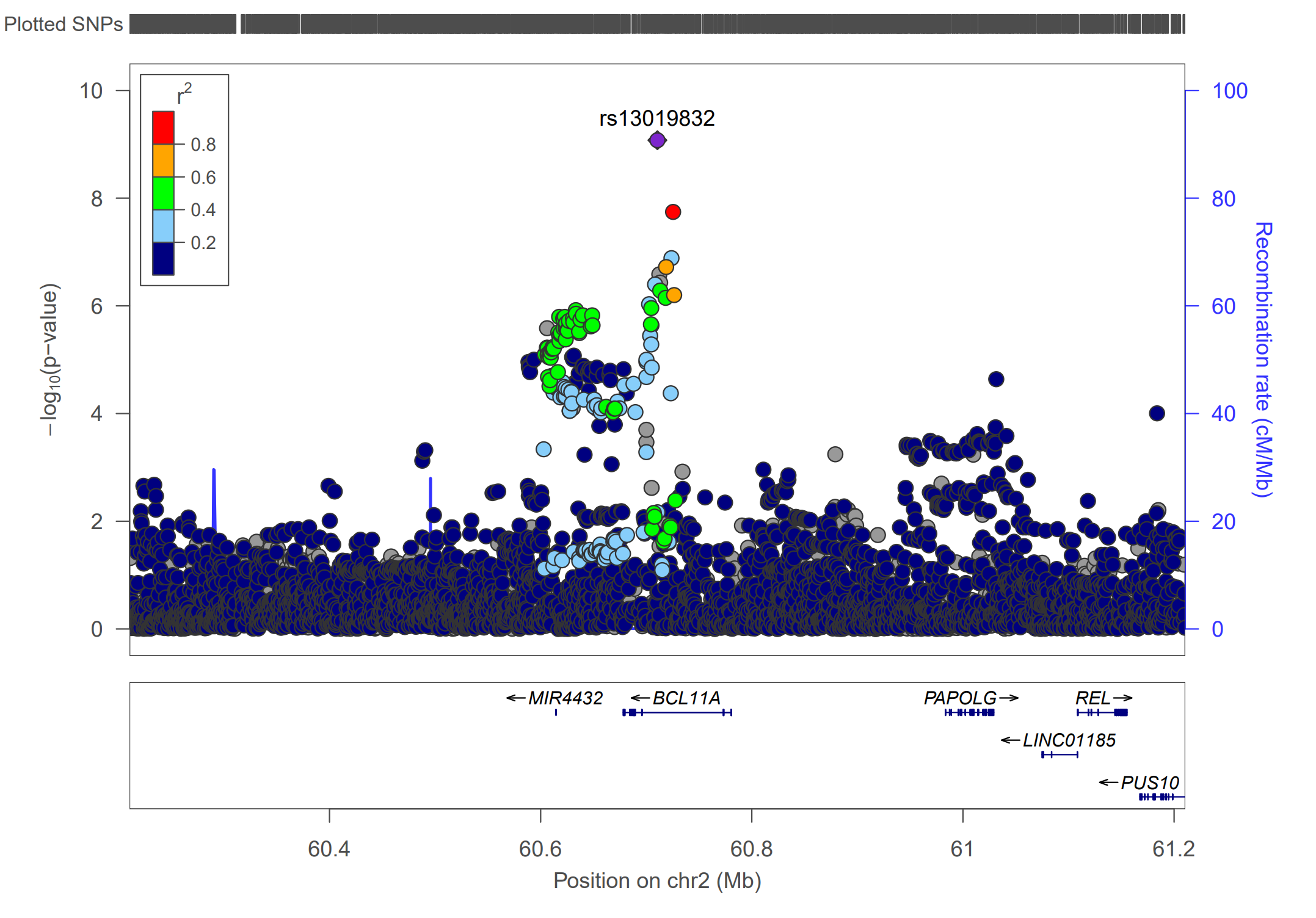

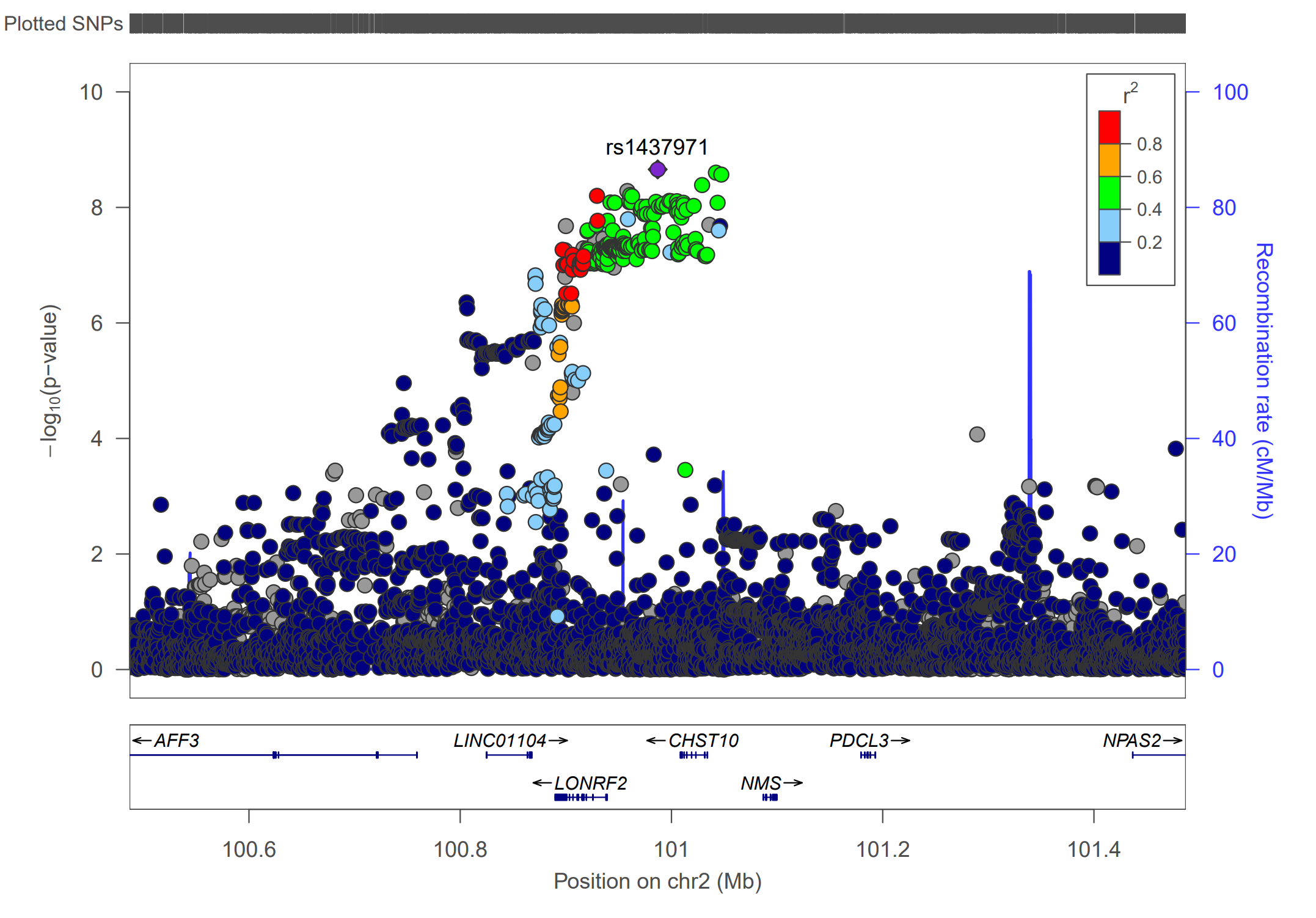


Locus 1, rs13019832; Physical demands Locus 2, rs1437971; Physical demands


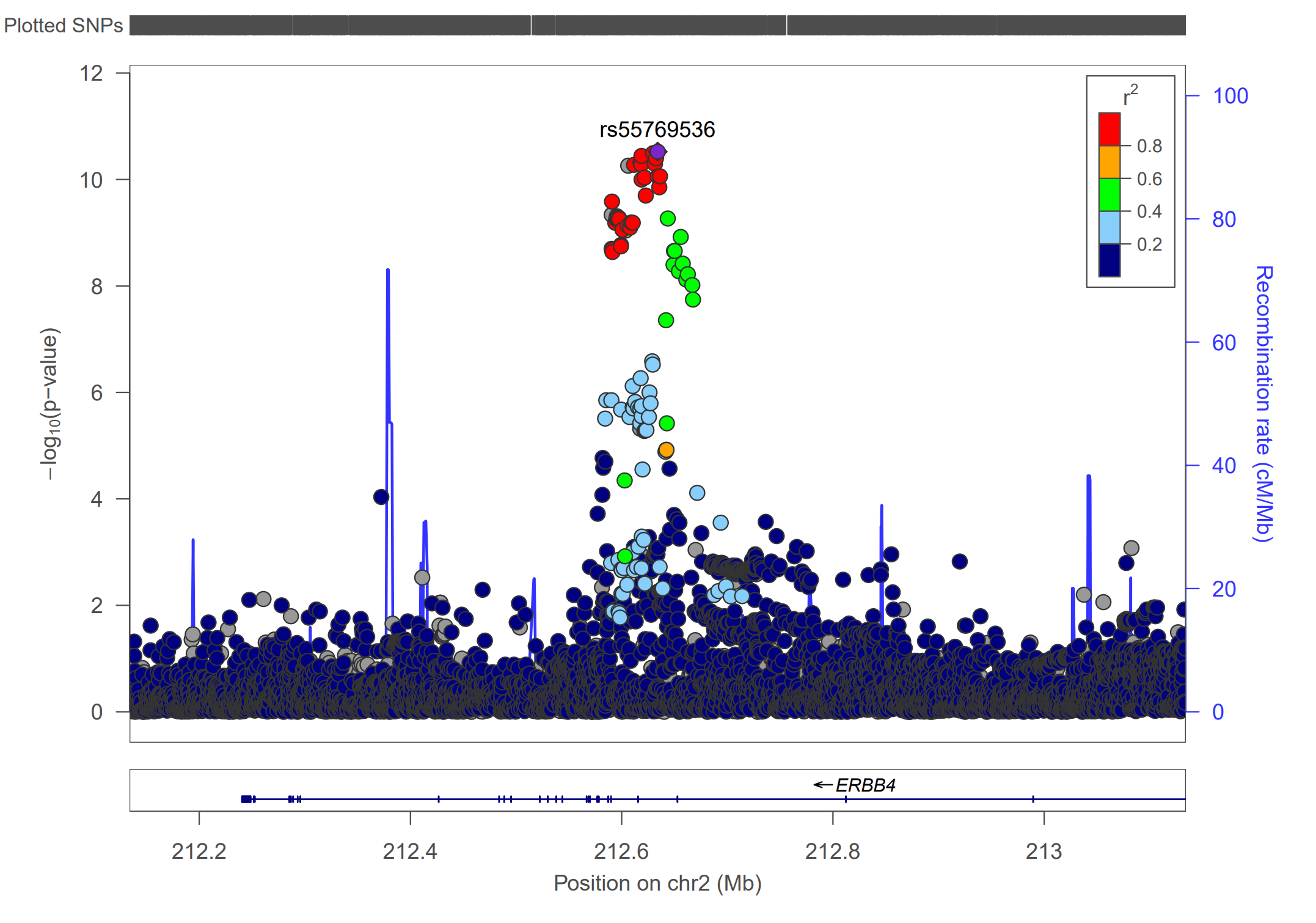


Locus 3, rs55769536; Physical demands


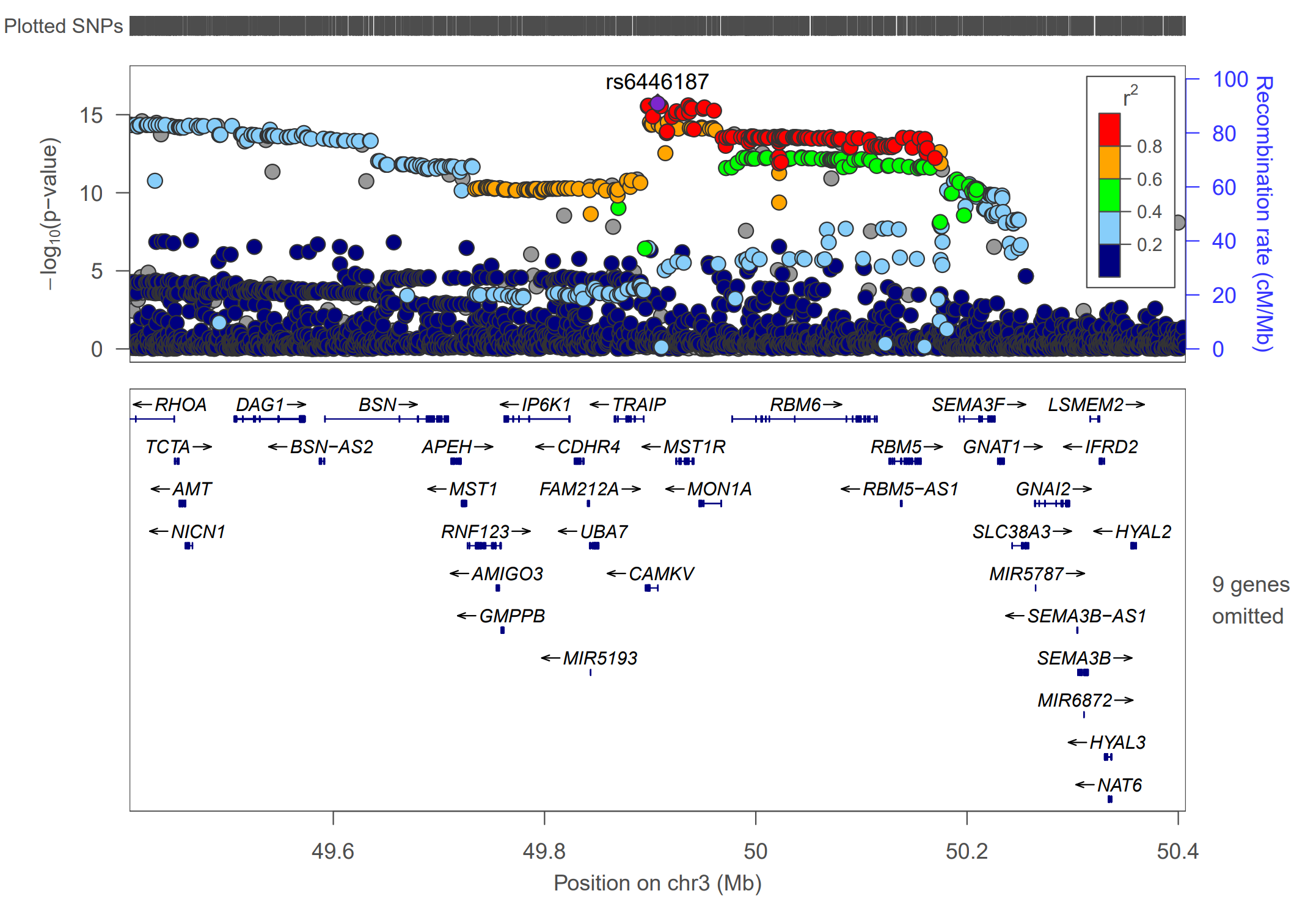

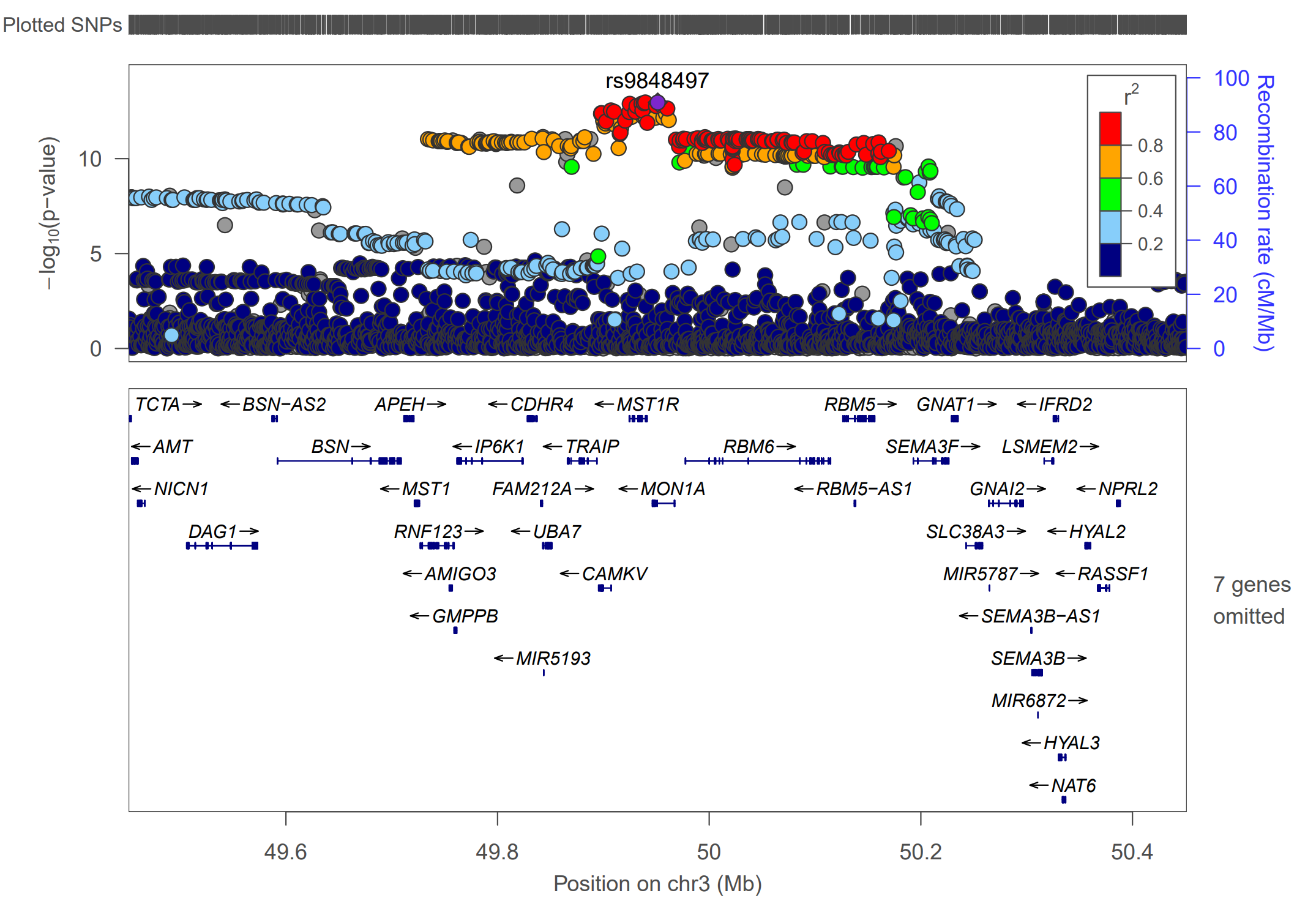


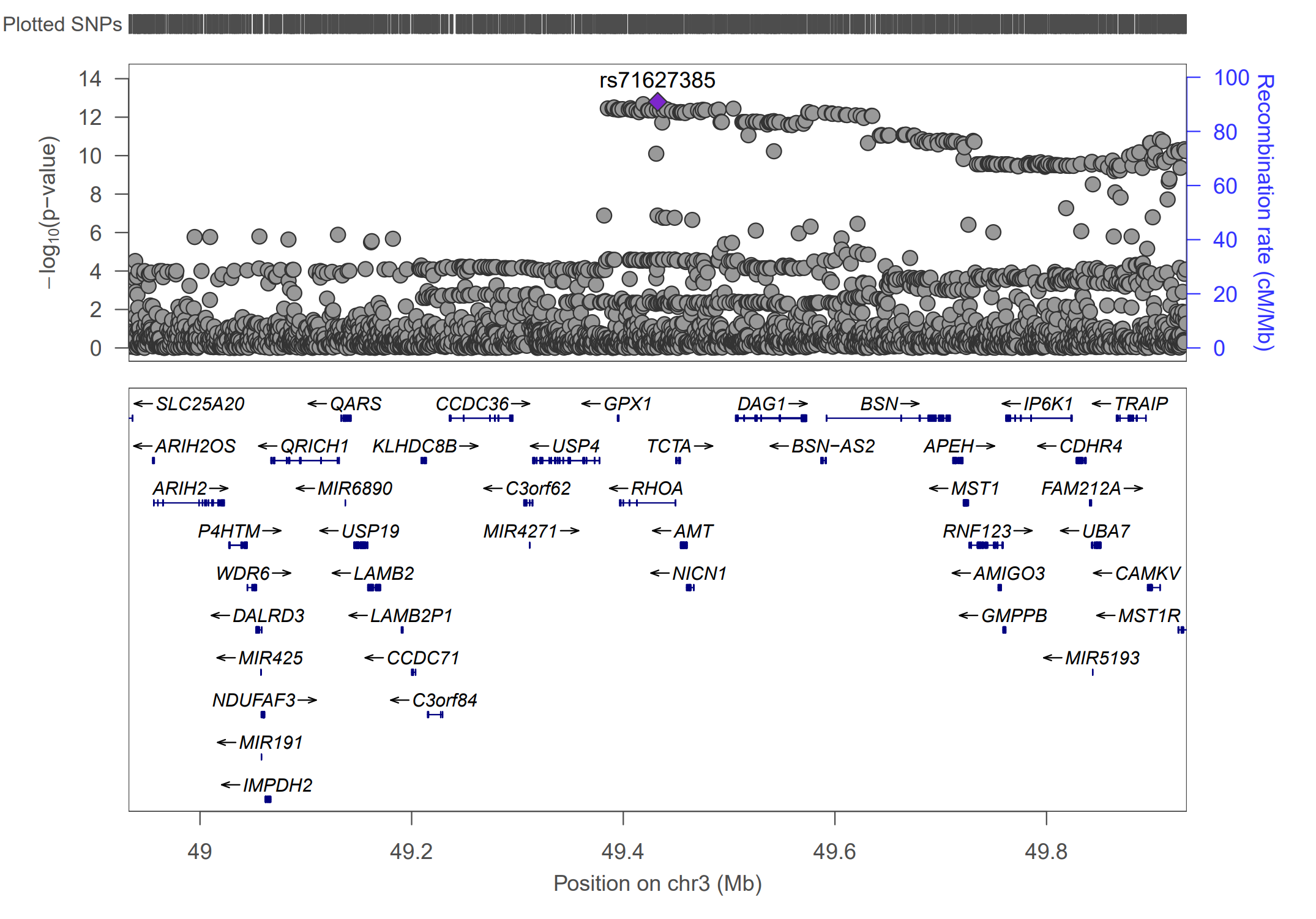

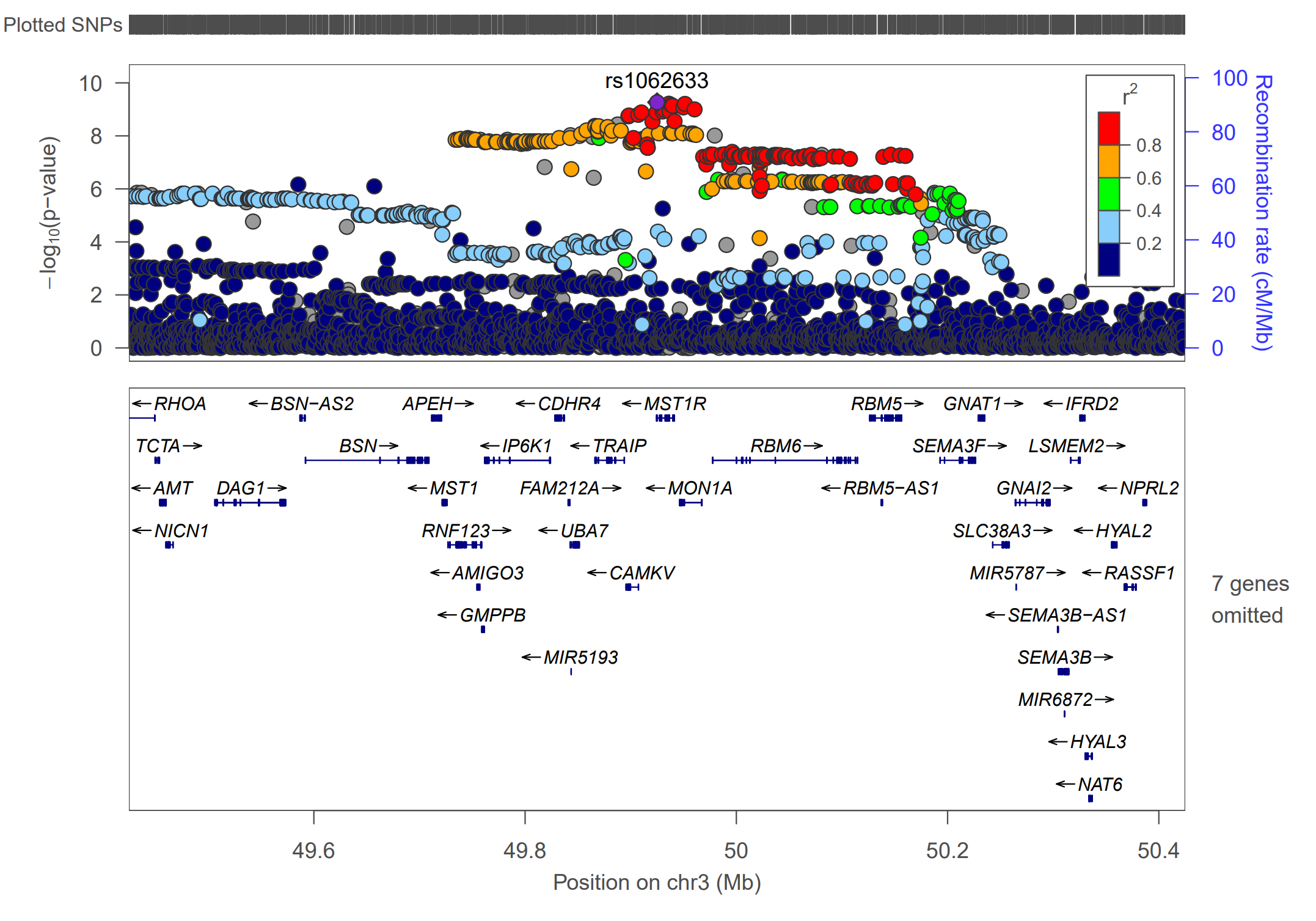


Locus 4, rs6446187; Information demands (top left), Innovation (rs9848497; top right), Autonomy (rs71627385; bottom left), Complexity (rs1062633; bottom right).


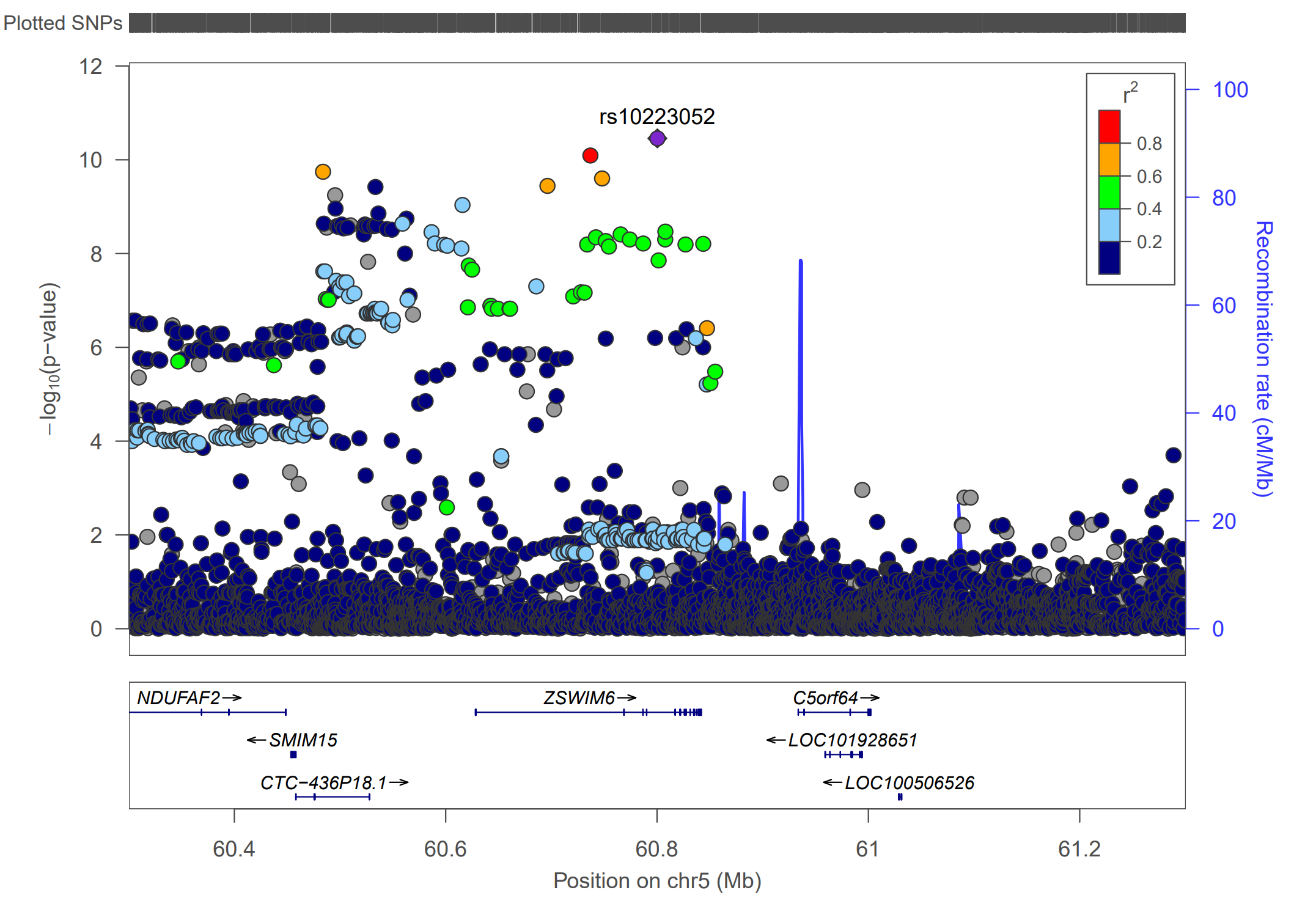

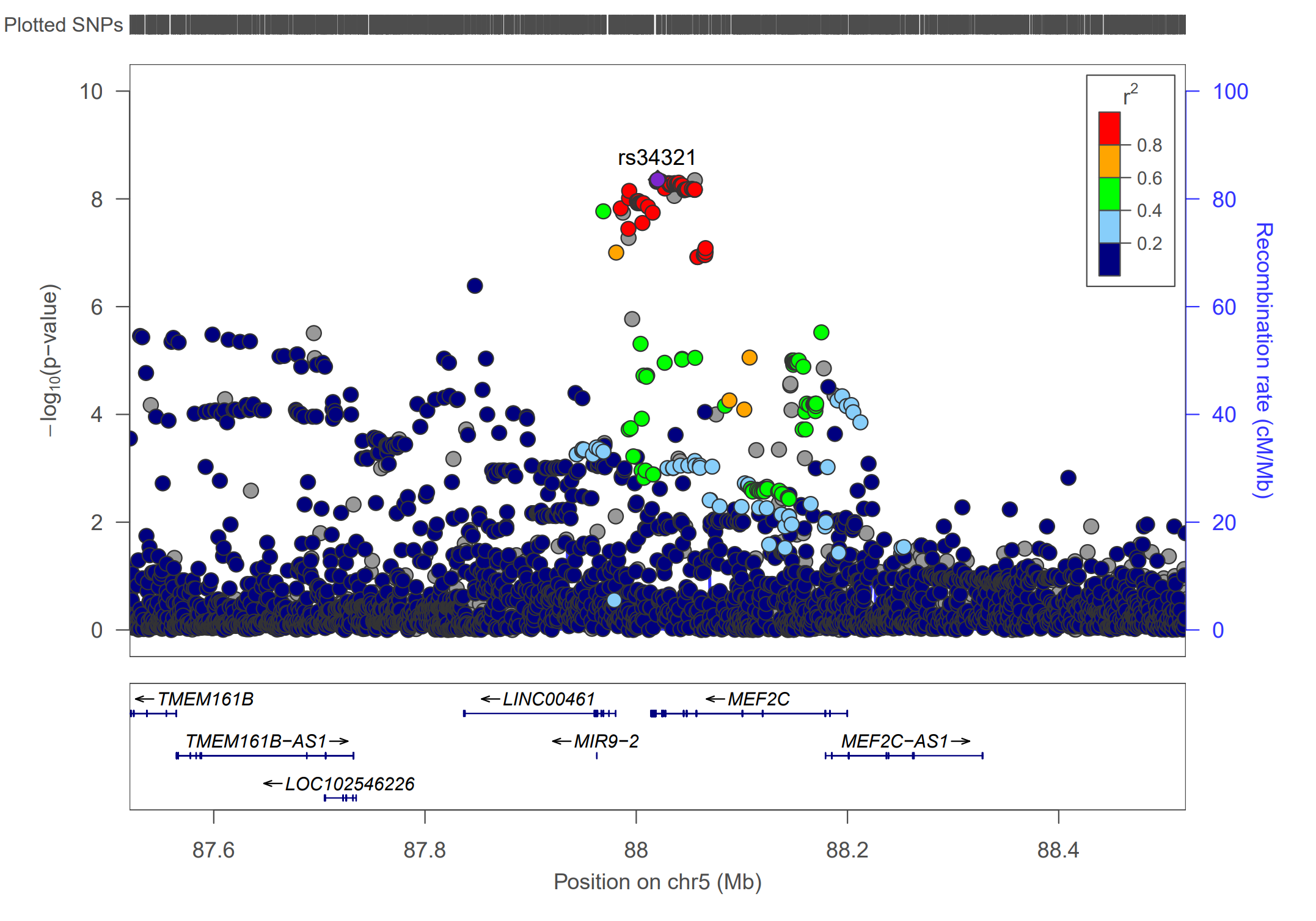


Locus 5, rs10223052; Information demands Locus 6, rs34321; Physical demands


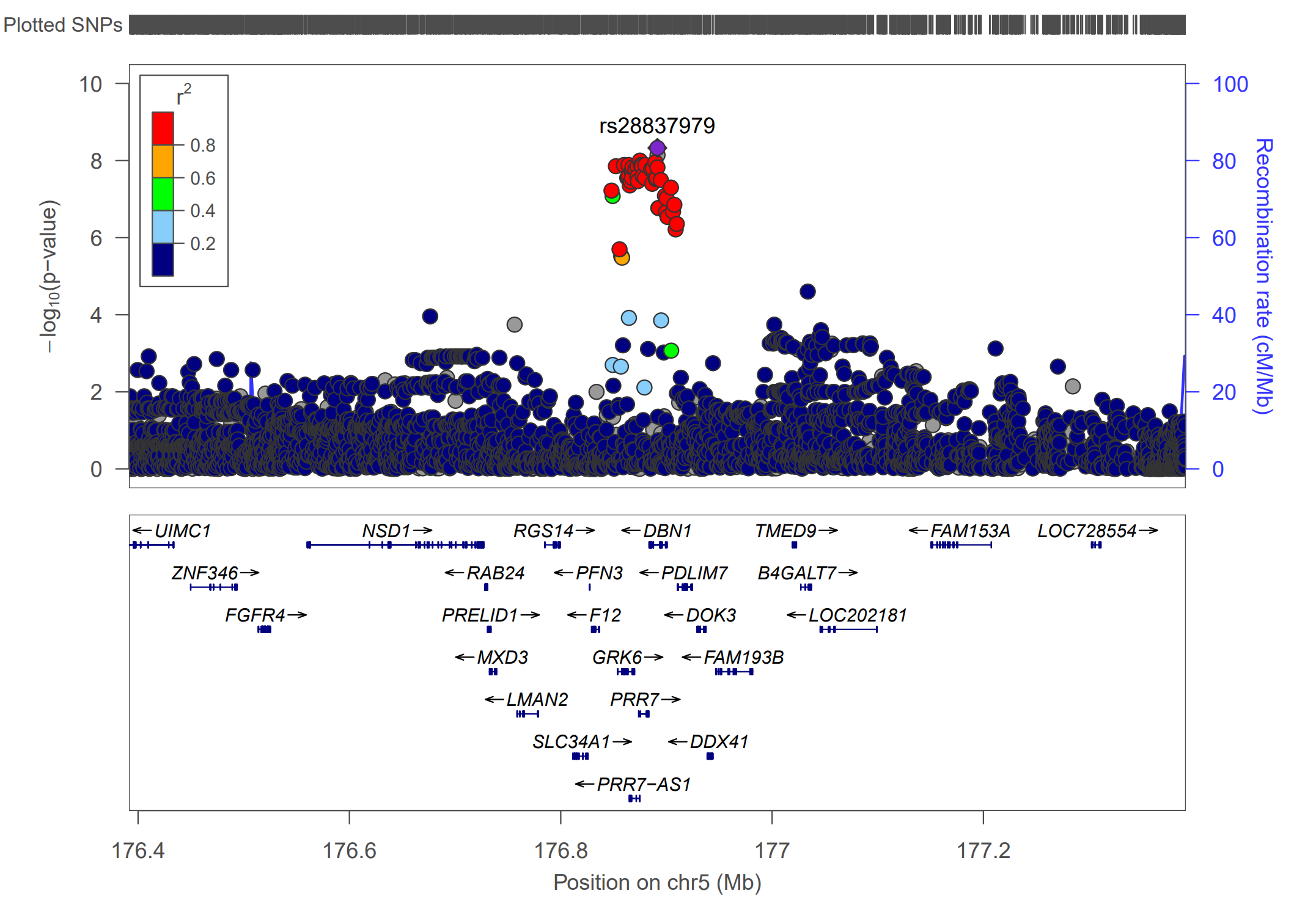


Locus 7, rs28837979; Physical demands


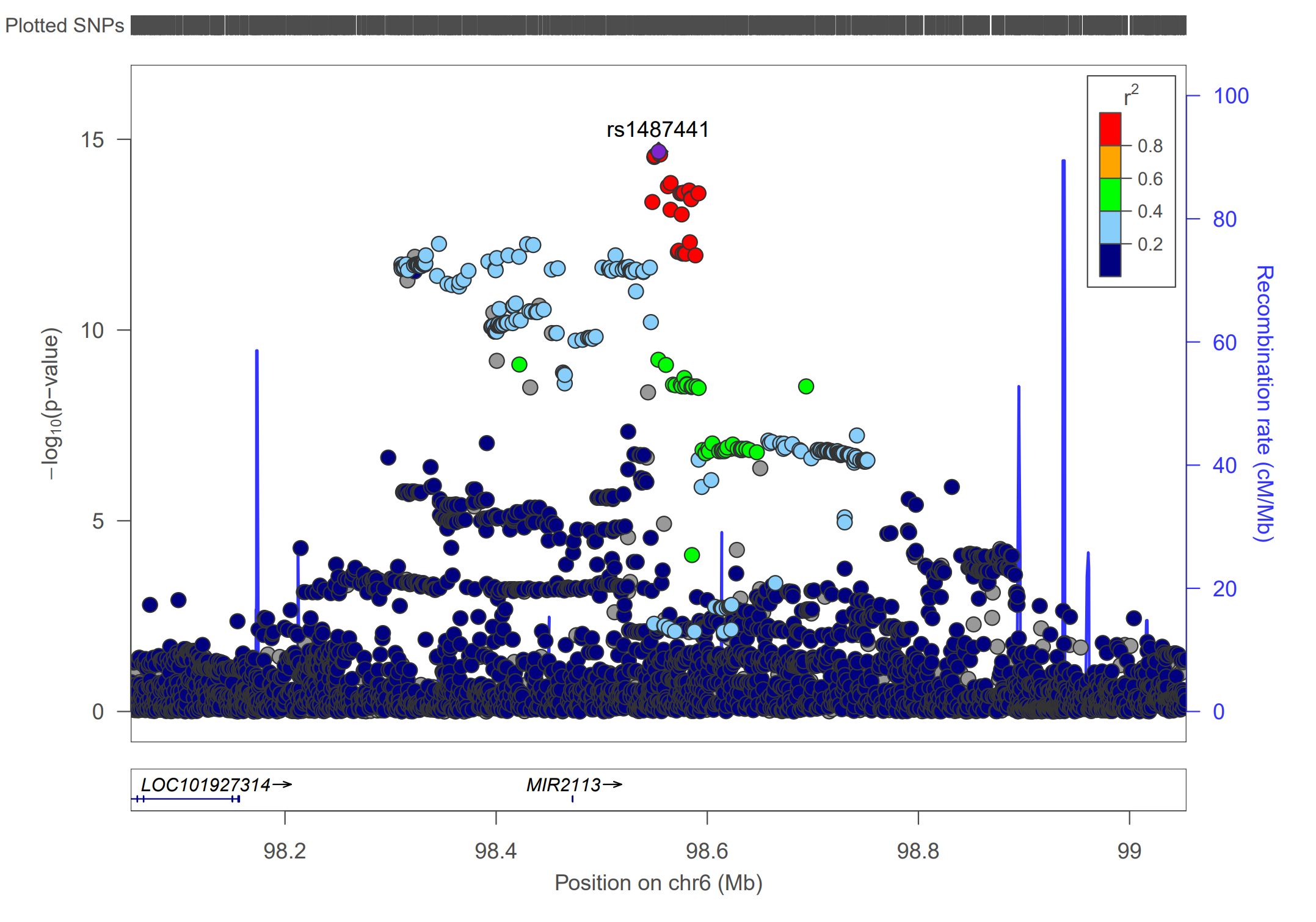

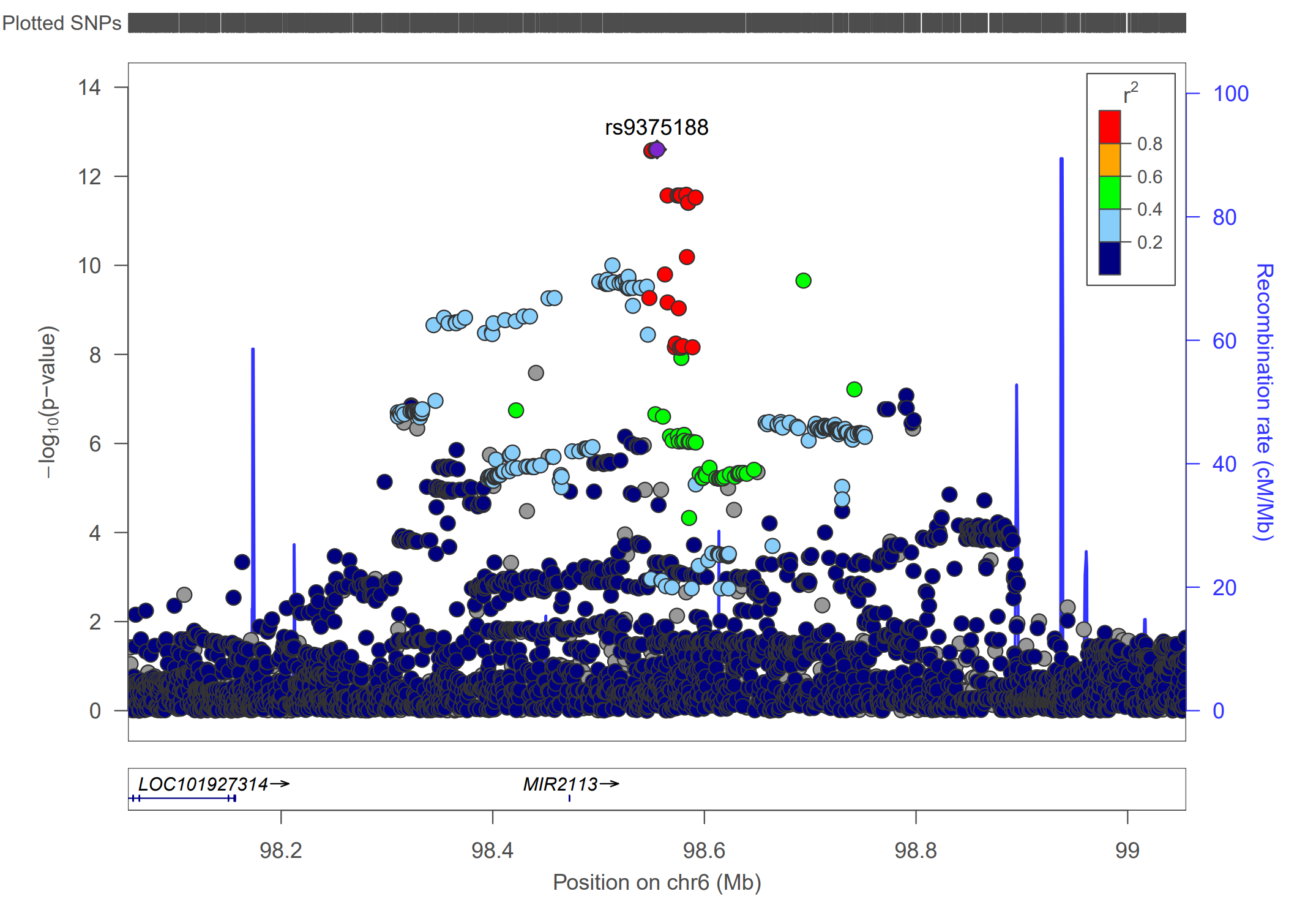


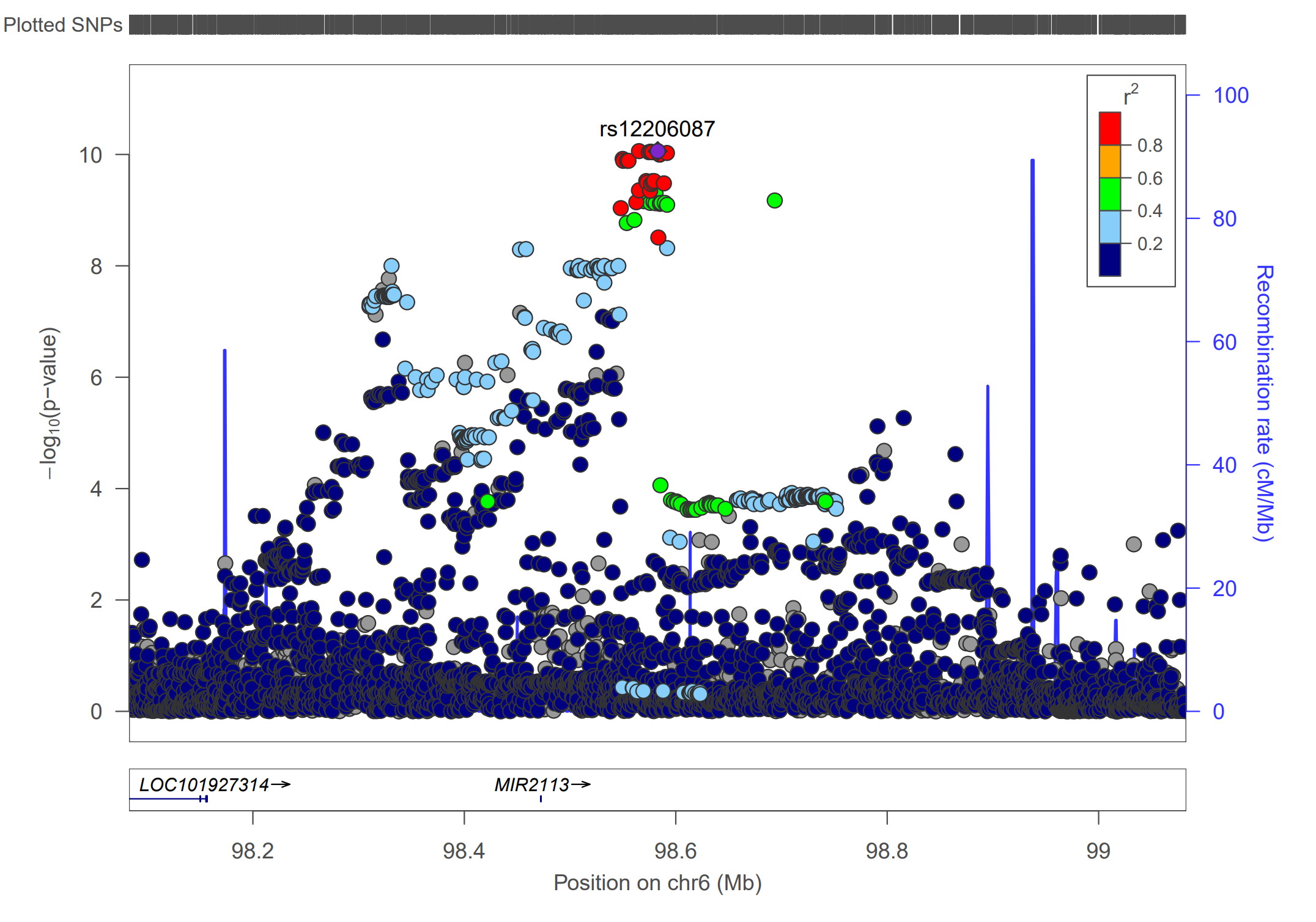


Locus 8, rs1487441; Information demands (top left), Autonomy (rs9375188; top right), Physical demands (rs12206087; bottom)


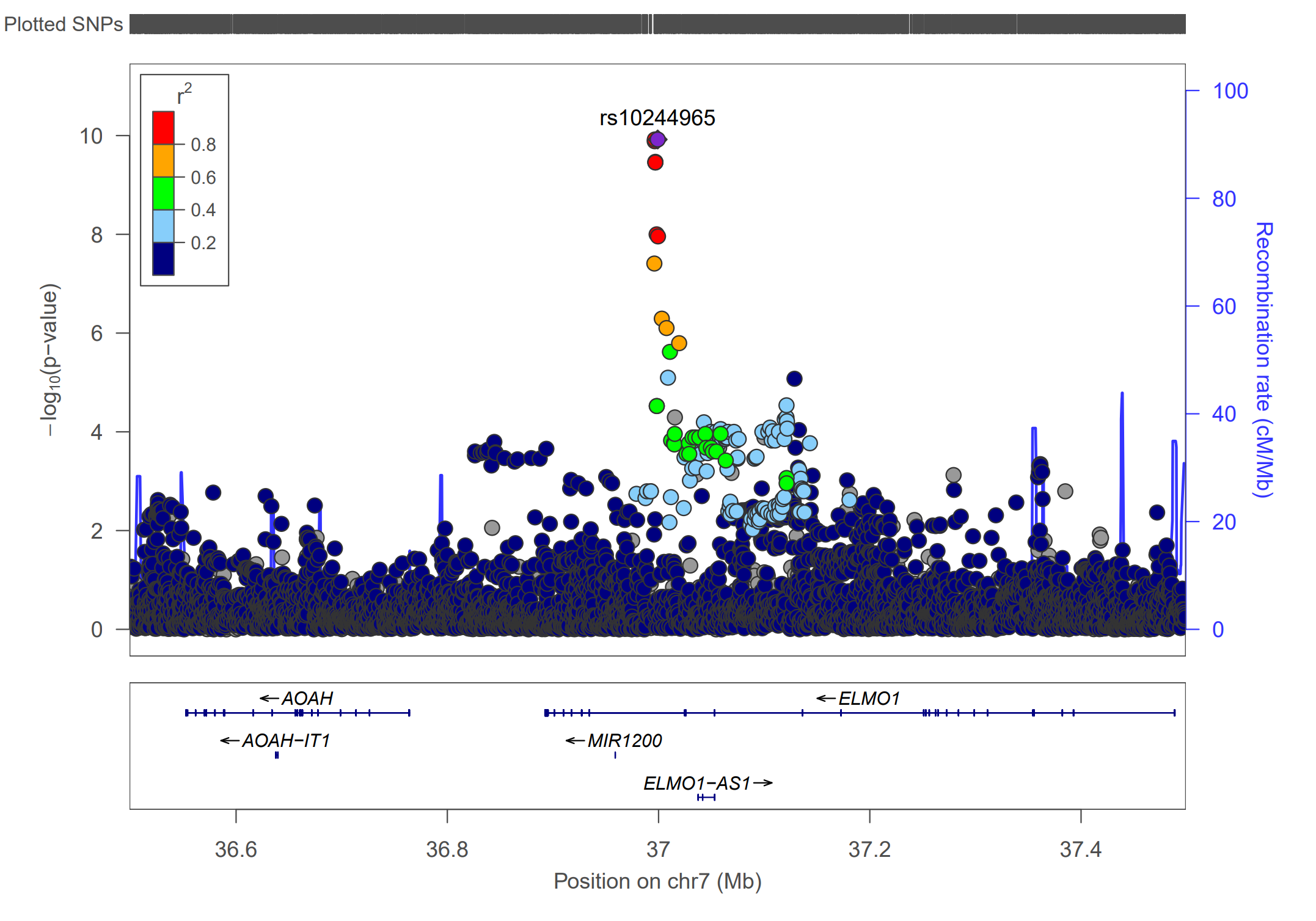

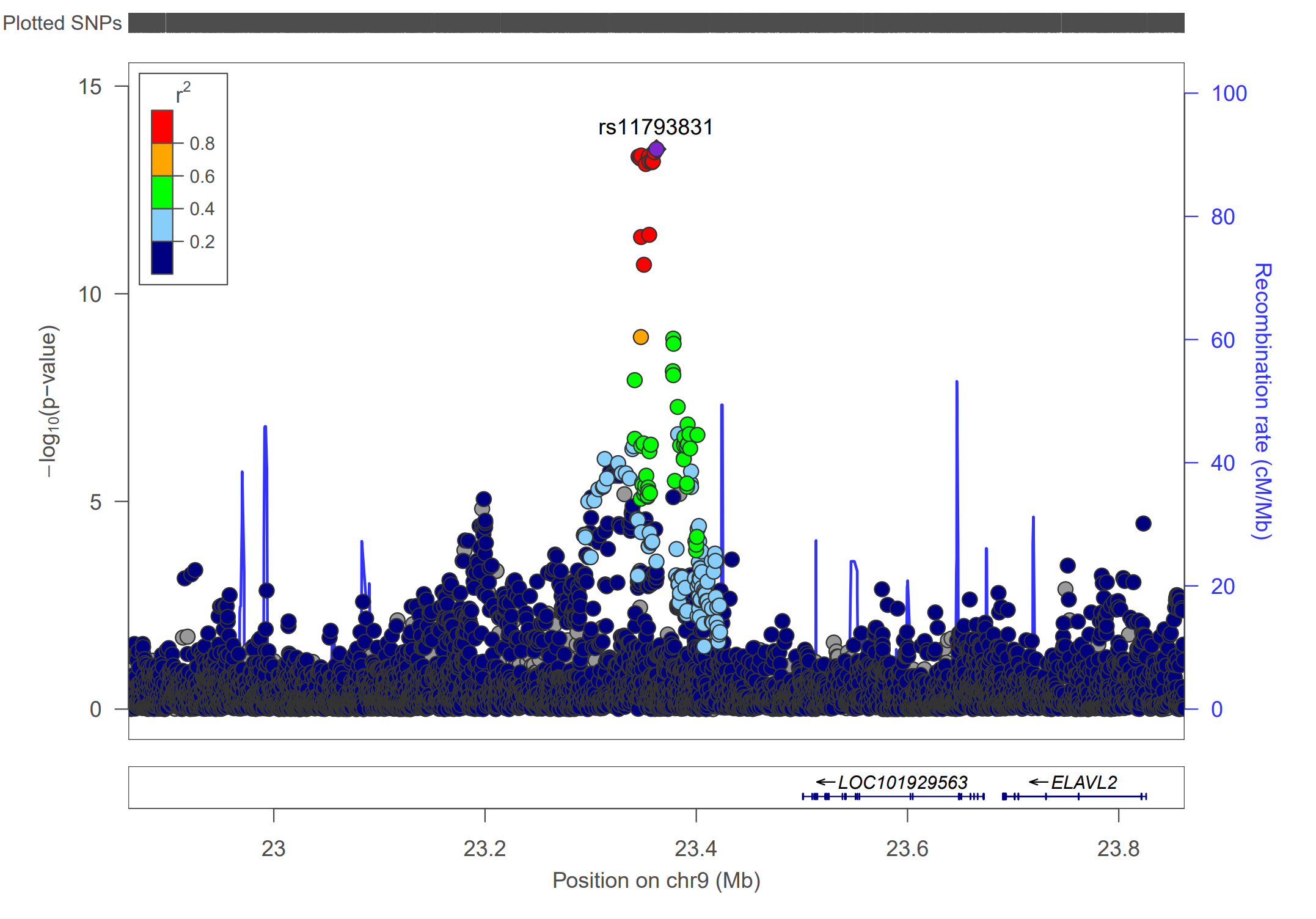


Locus 9, rs10244965; Autonomy Locus 11, rs11793831; Information demands


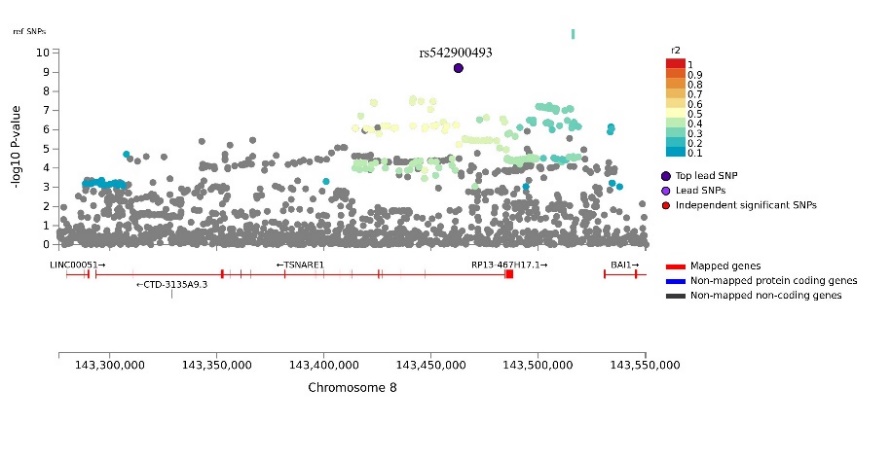
 Locus 10, rs542900493; Physical demands


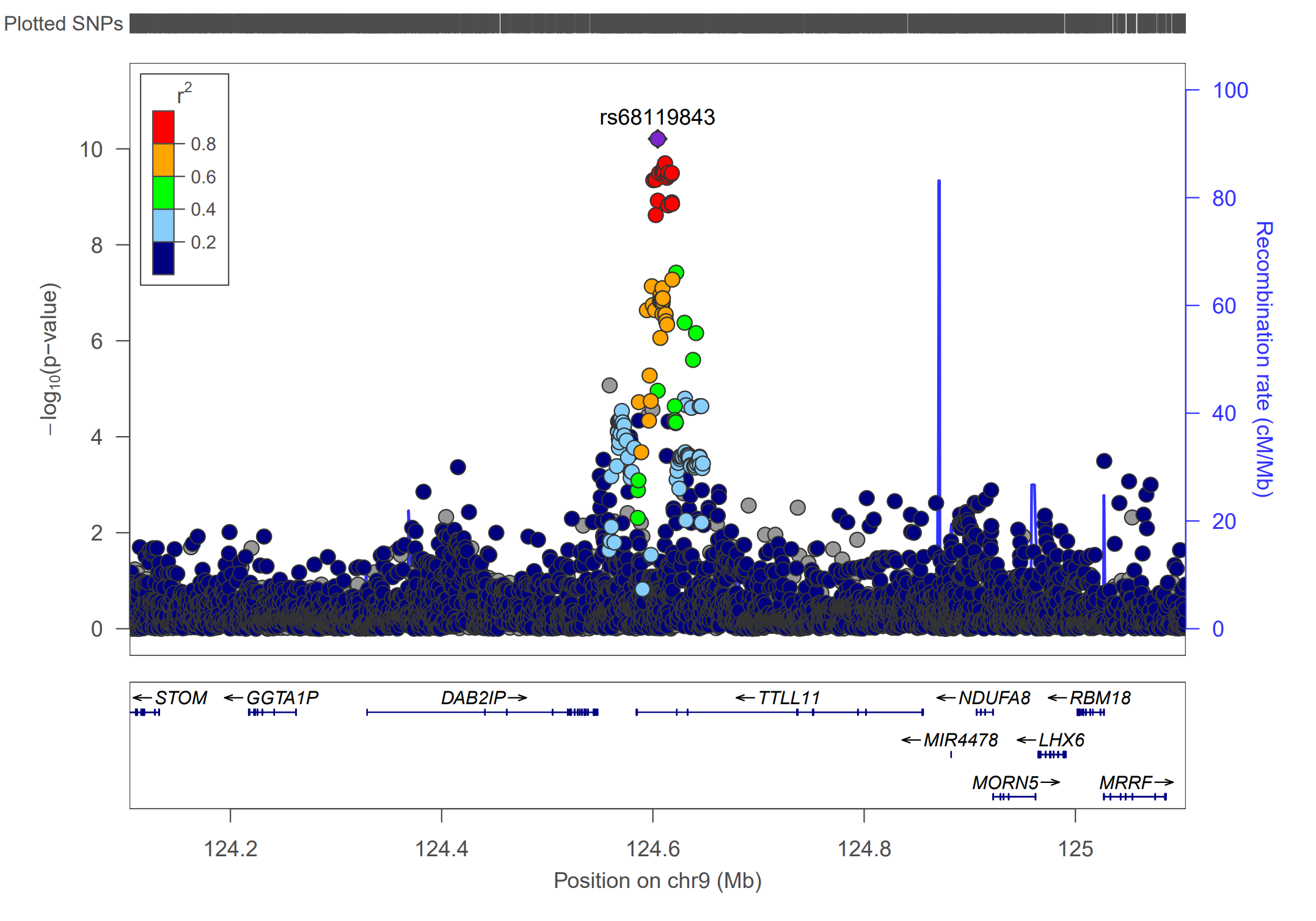

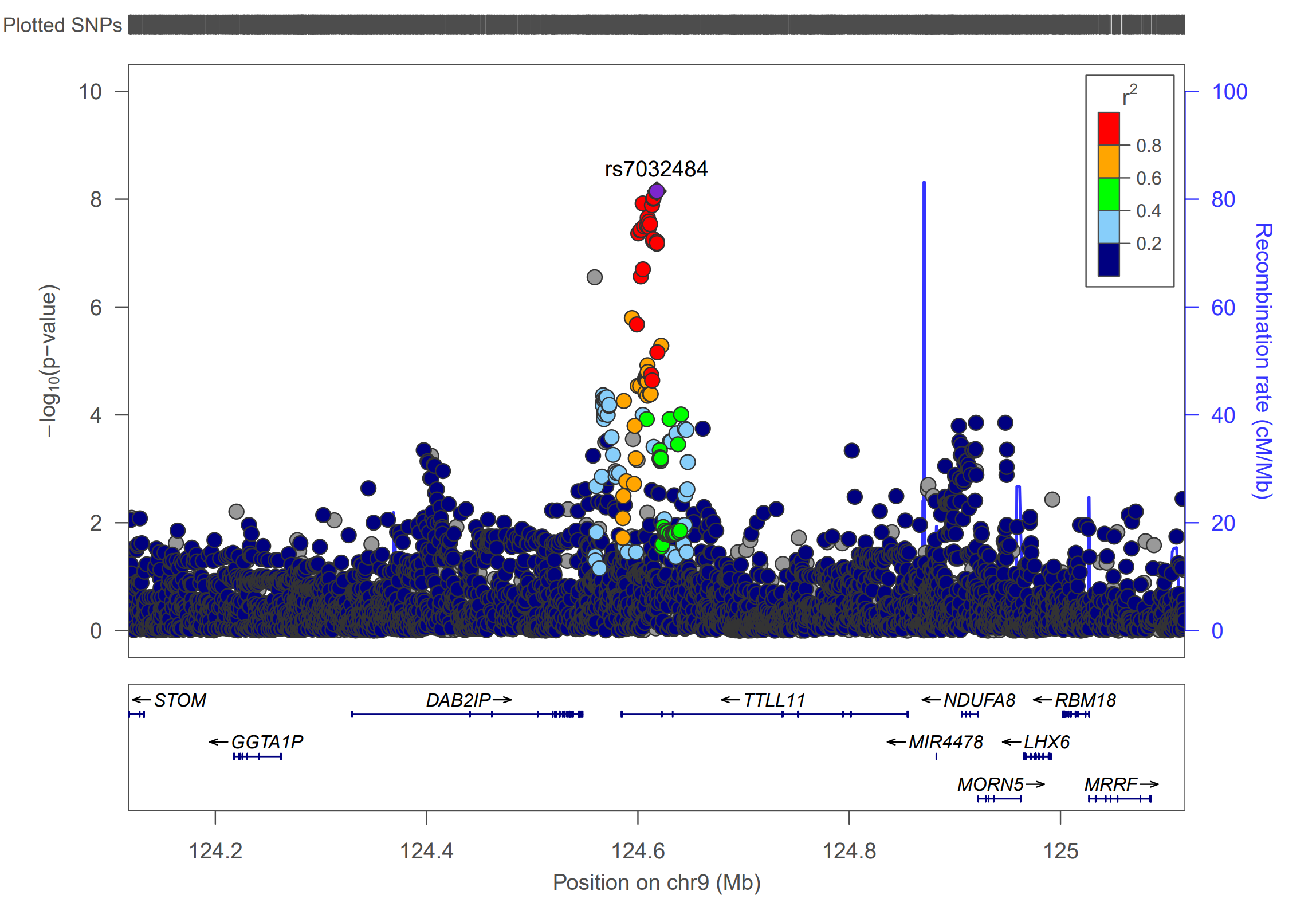
Locus 12, rs68119843; Information demands (left), Complexity (rs7032484;right)


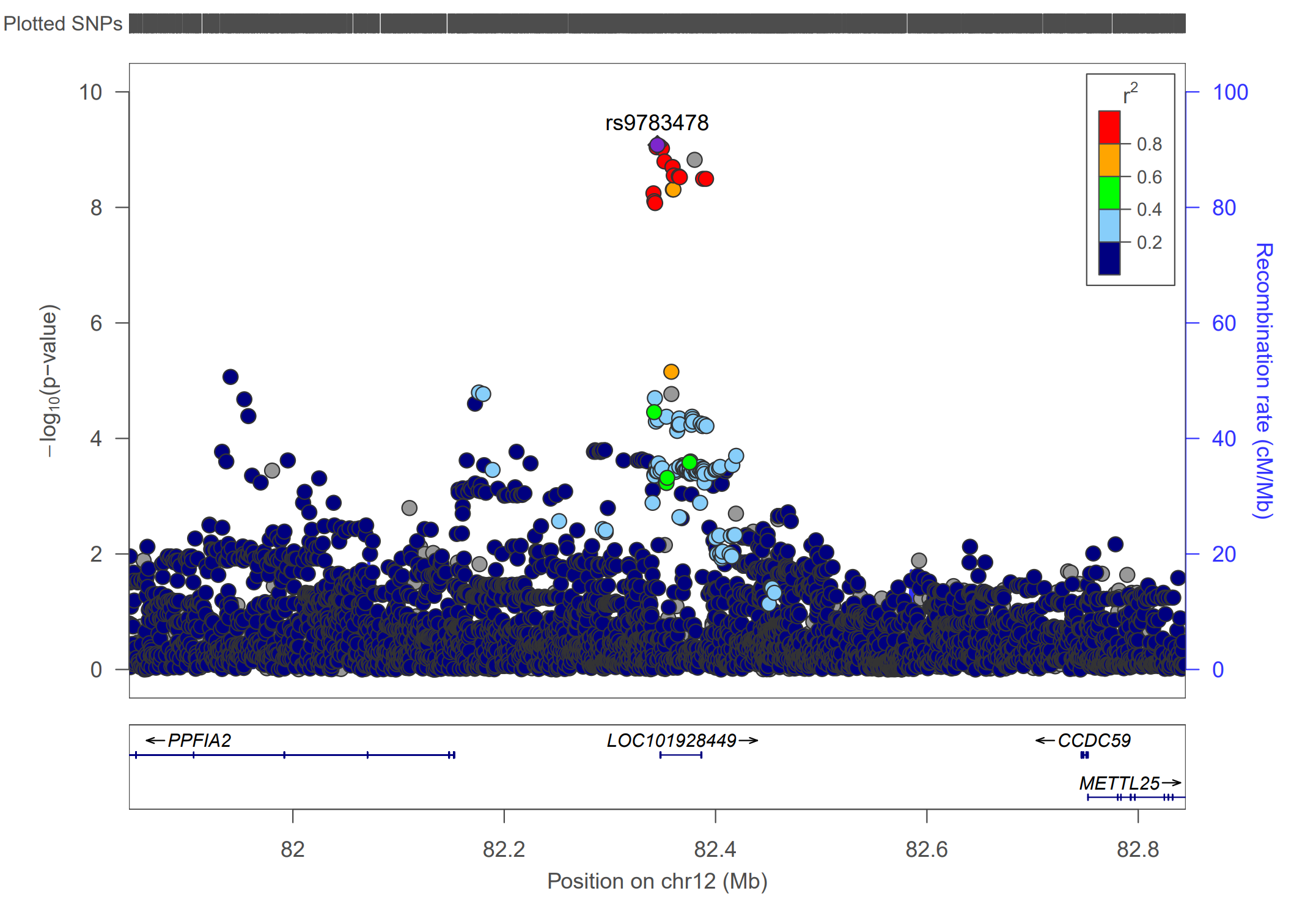

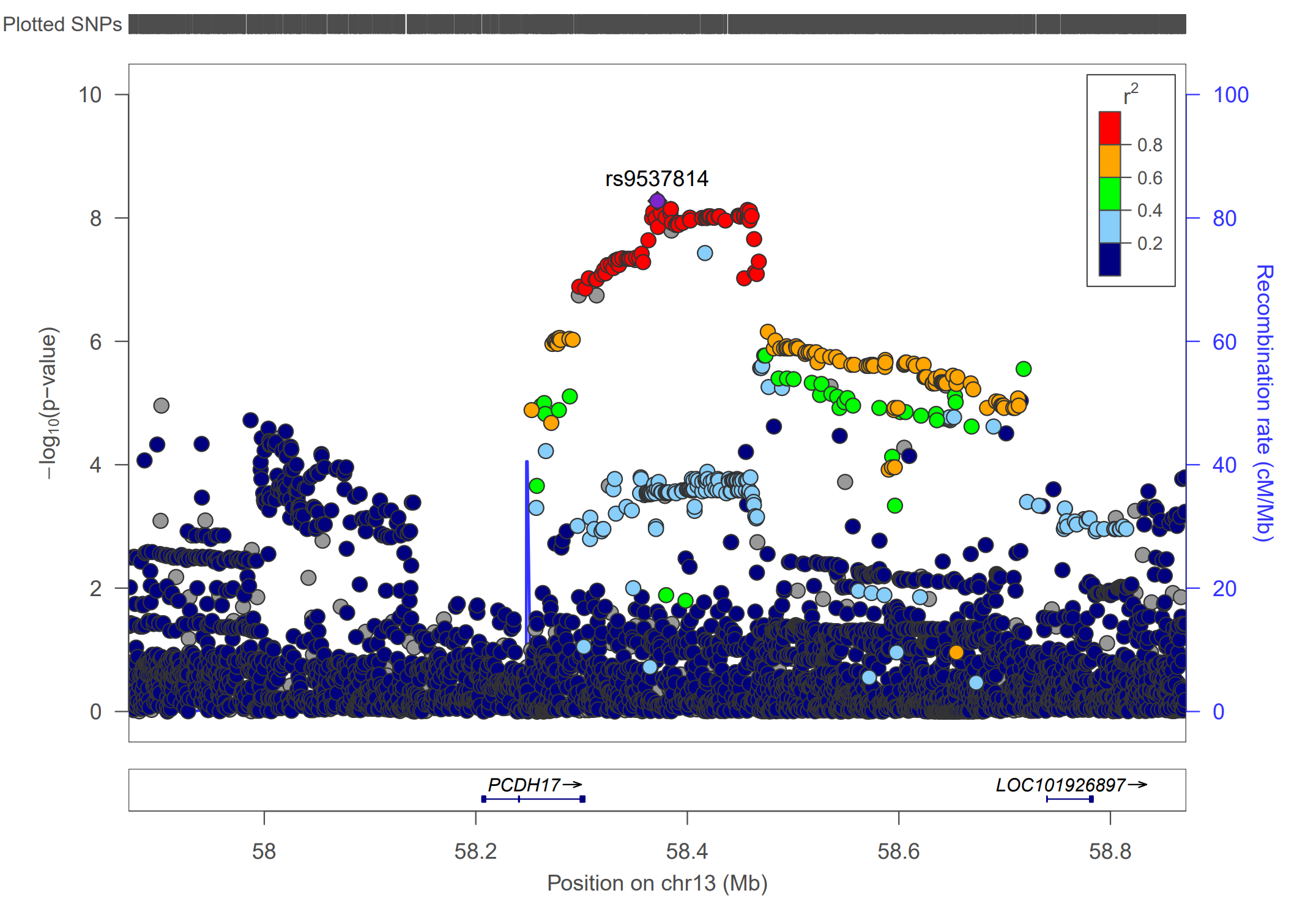


Locus 13, rs9783478; Innovation Locus 14, rs9537814; Innovation


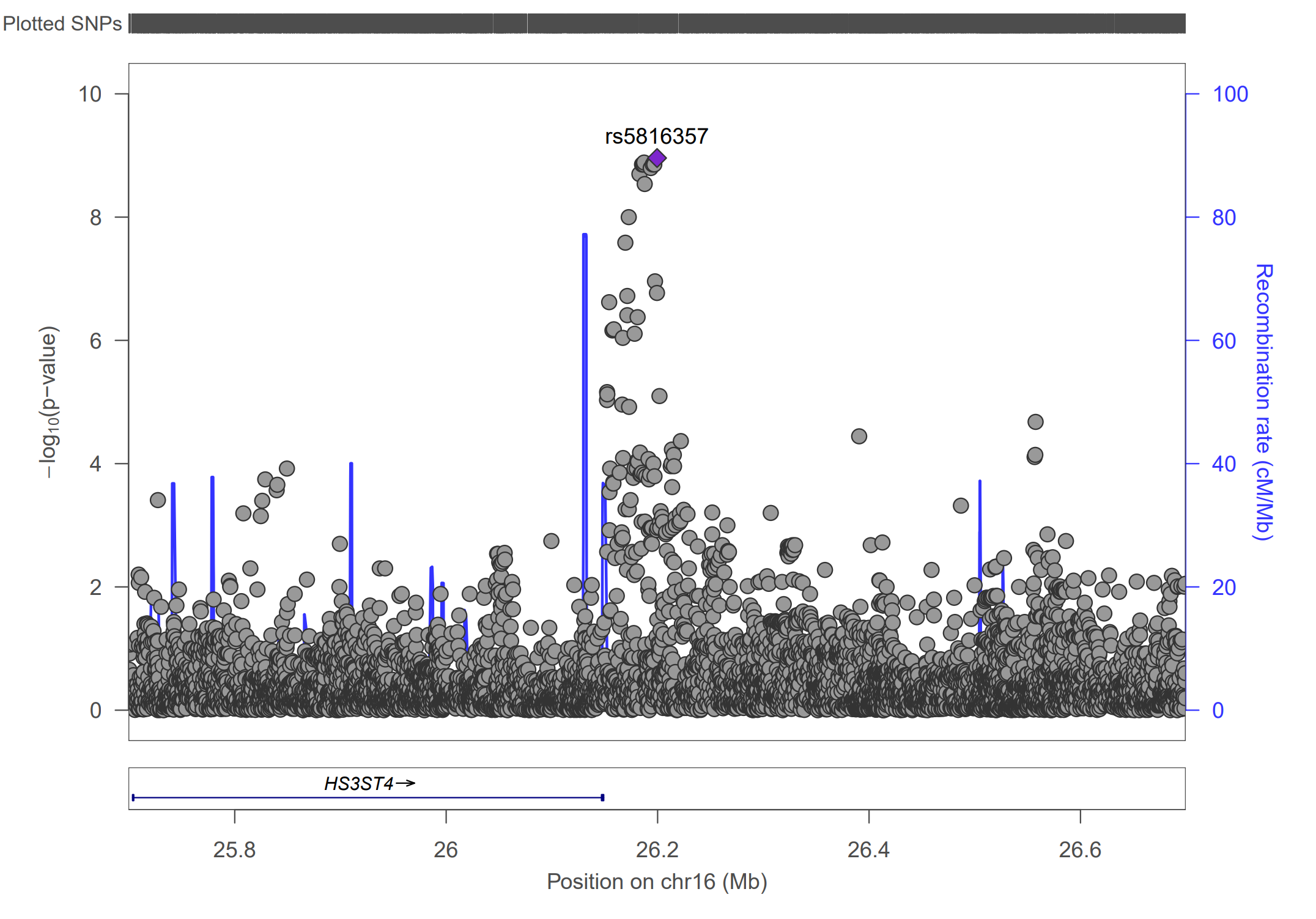


Locus 15, rs5816357; Physical demands


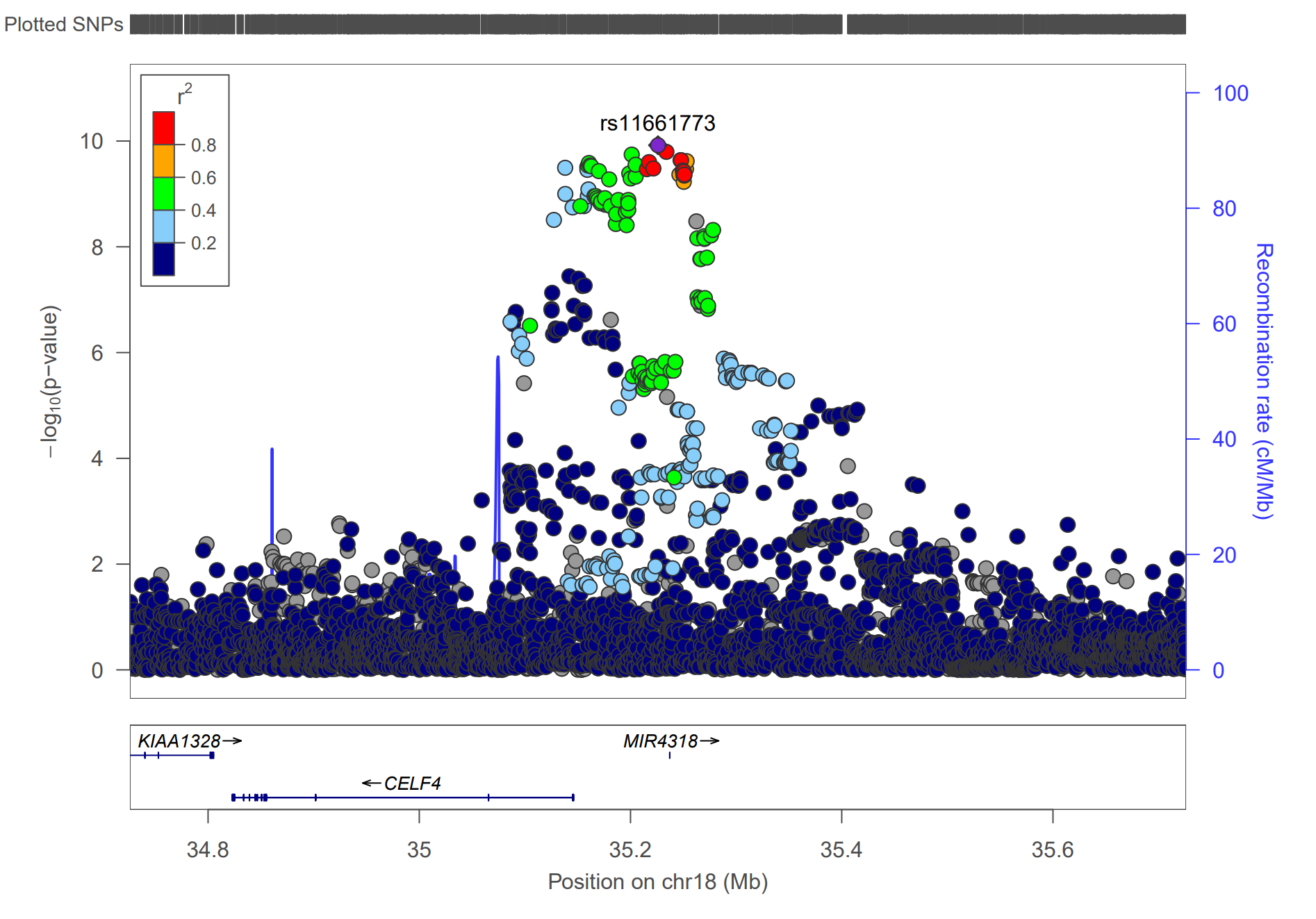

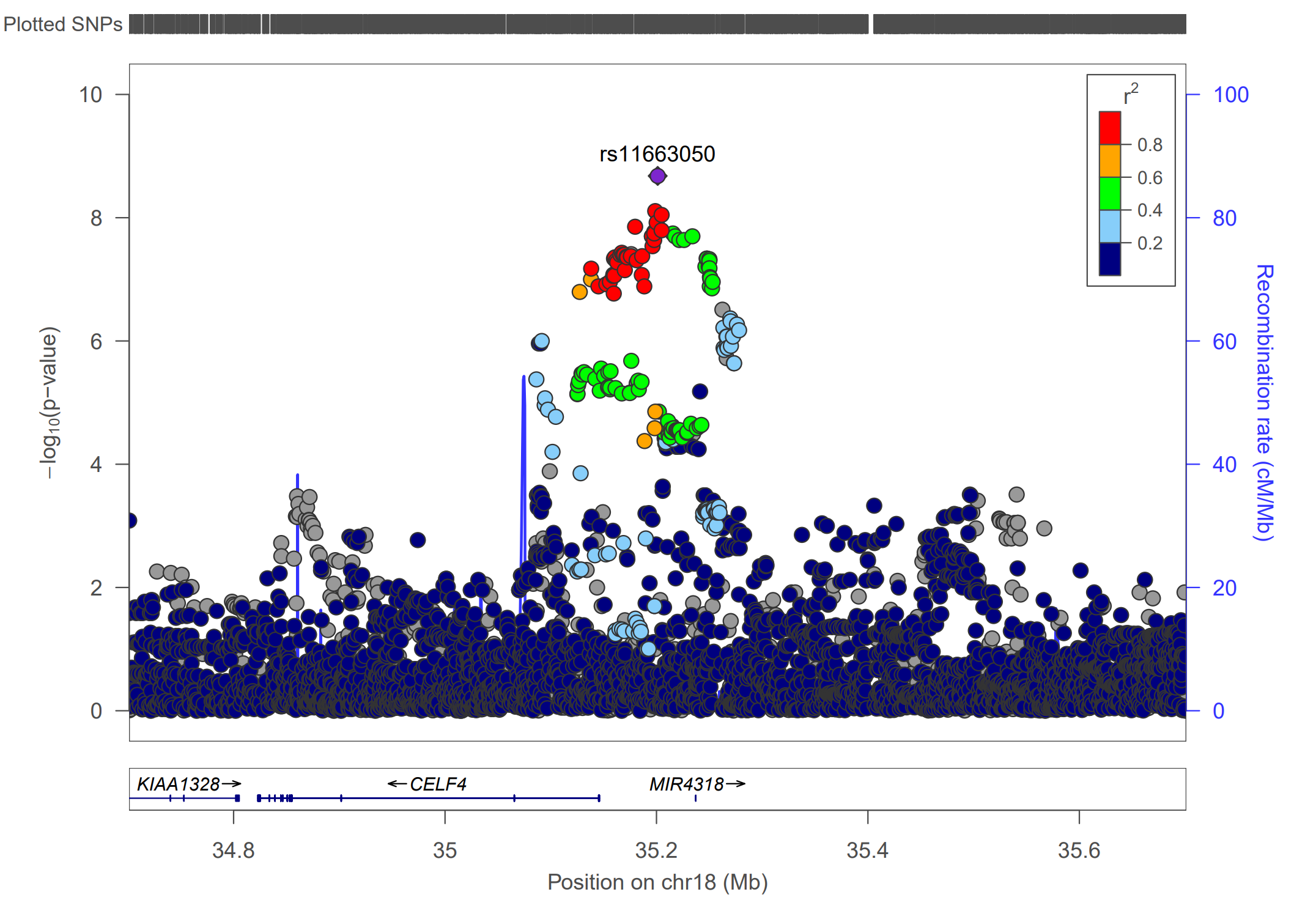


Locus 16, rs11661773; Information demands (left) and Complexity (rs11663050; right).

**Figure S4. Regional association plots for the 16 identified loci, LD (r^2^) shown in the plots based on European populations**


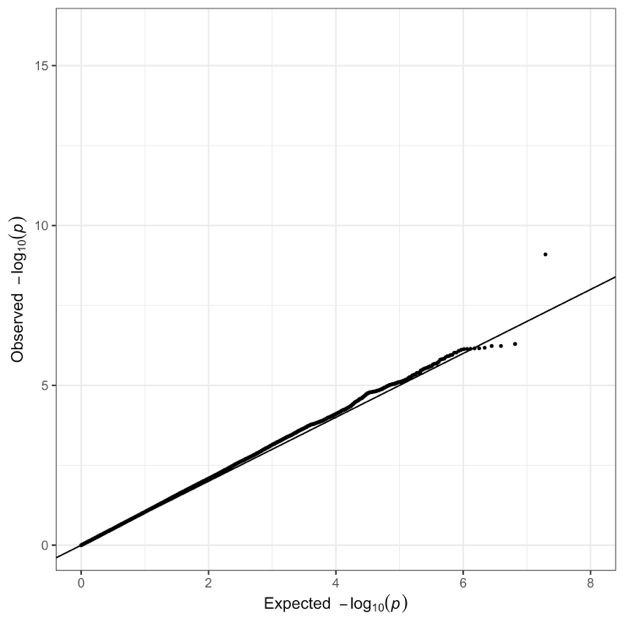

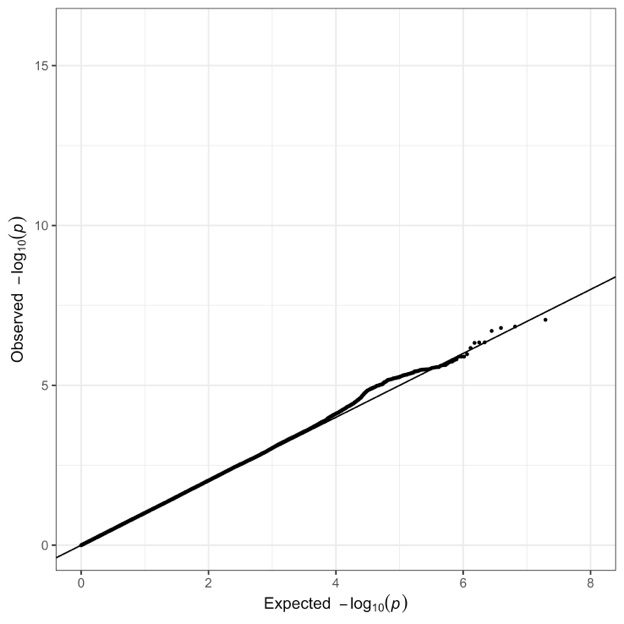


Complexity Autonomy


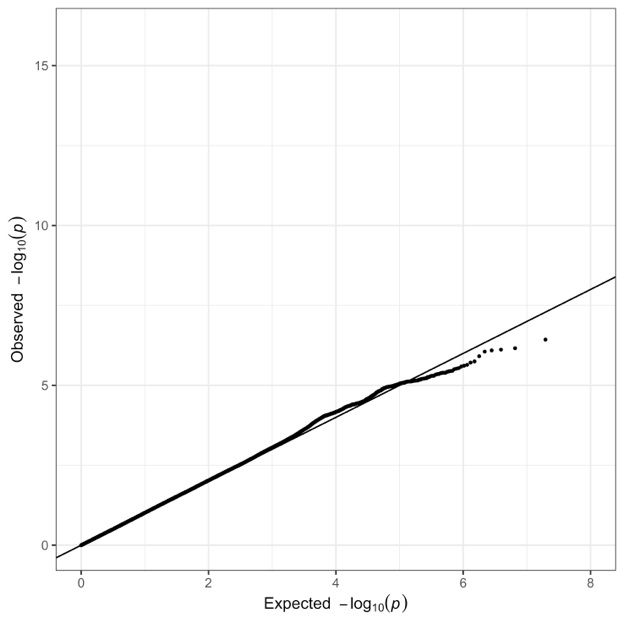

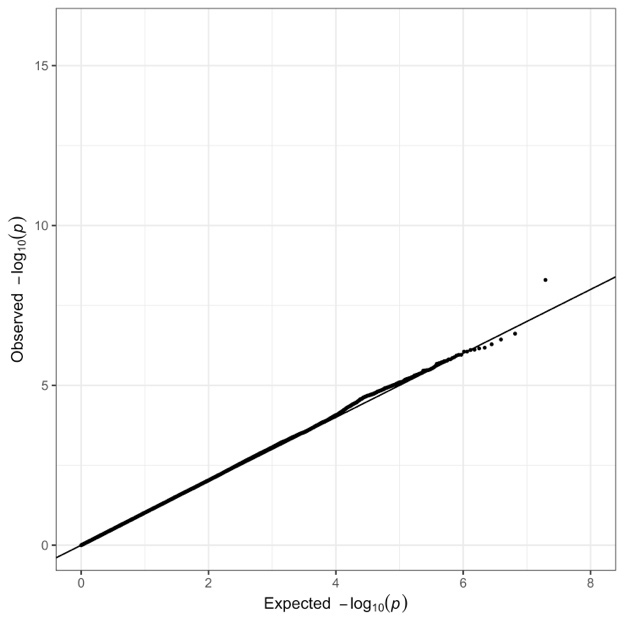


Innovation Information demands


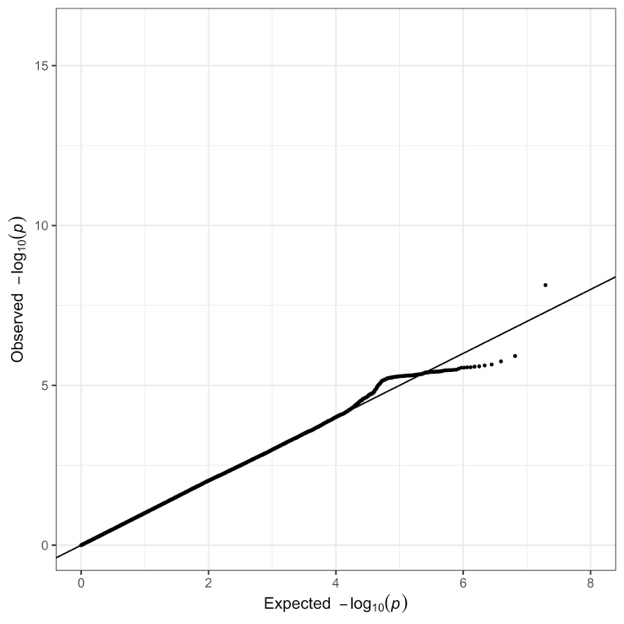

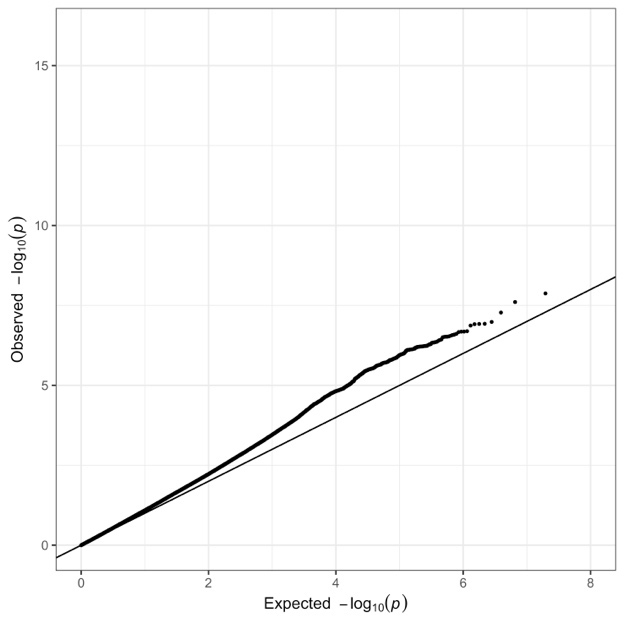


Emotional demands Physical demands

Figure S5. Quantile-quantile plots of Z-scores for sex-differentiated genetic effects.


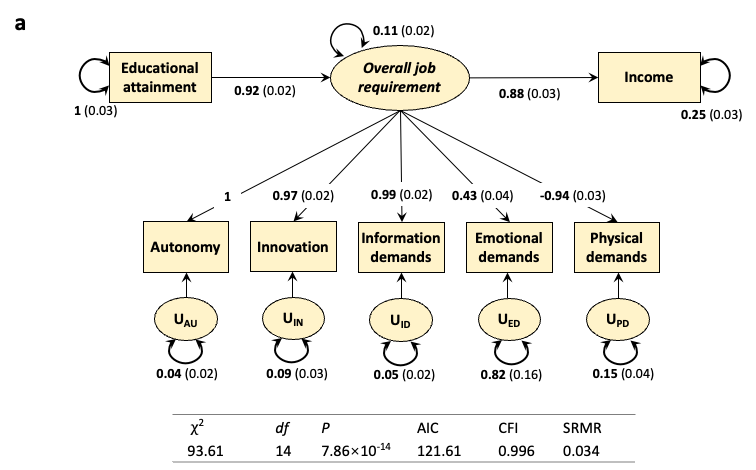


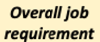

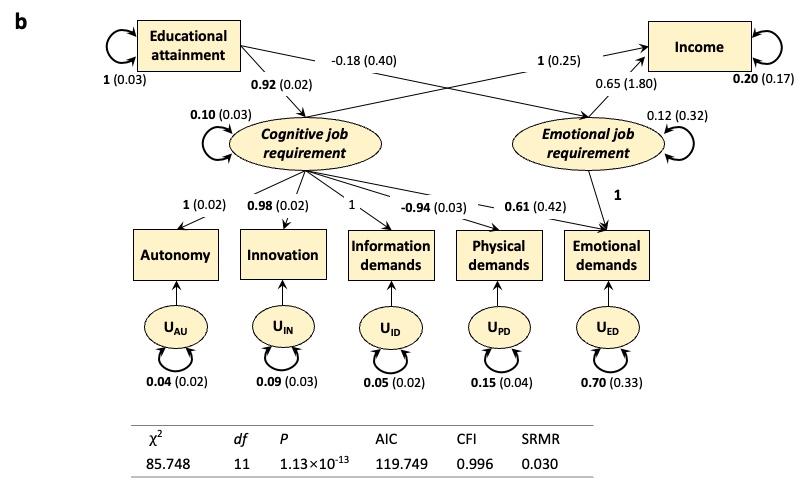


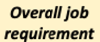

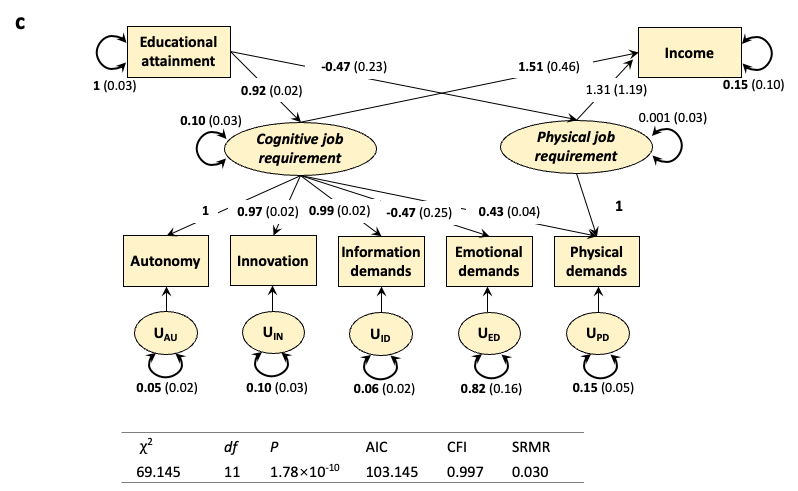


**Figure S6. Alternative Genomic SEM models.** Model a removed the direct path from educational attainment to income for one latent factor model. Model b added a latent emotional requirement into the model. Model c added a latent physical requirement into the model. ID: information demands, PD: physical demands, AU: autonomy, IN: Innovation, ED: emotional demands.


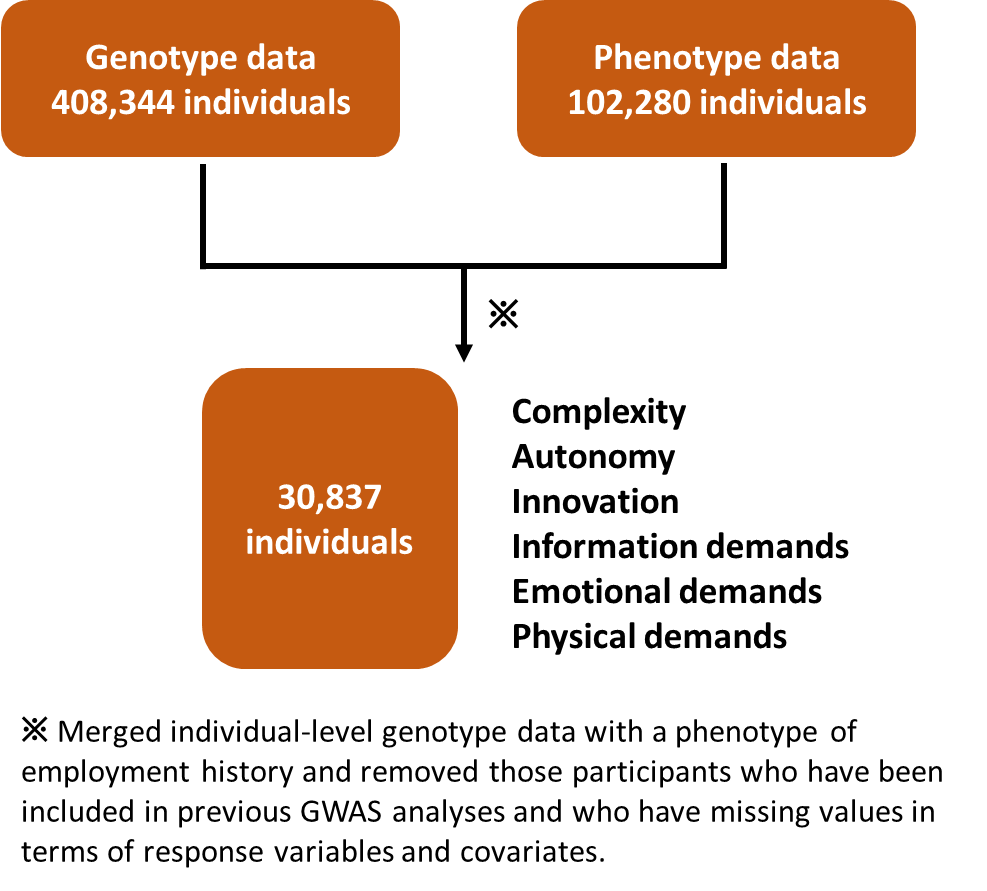


**Figure S7. Data preparation for the UK Biobank follow-up cohort.** the replication samples comprised of 30,387 individuals with genome data and valid job information of the last job, but not included in the discovery phase because of lacking baseline job information; thus the UK Biobank replication samples were not overlapped with the discovery sample. The same QC criteria for participants and genetic markers used in the discovery phase were applied to the replication samples.


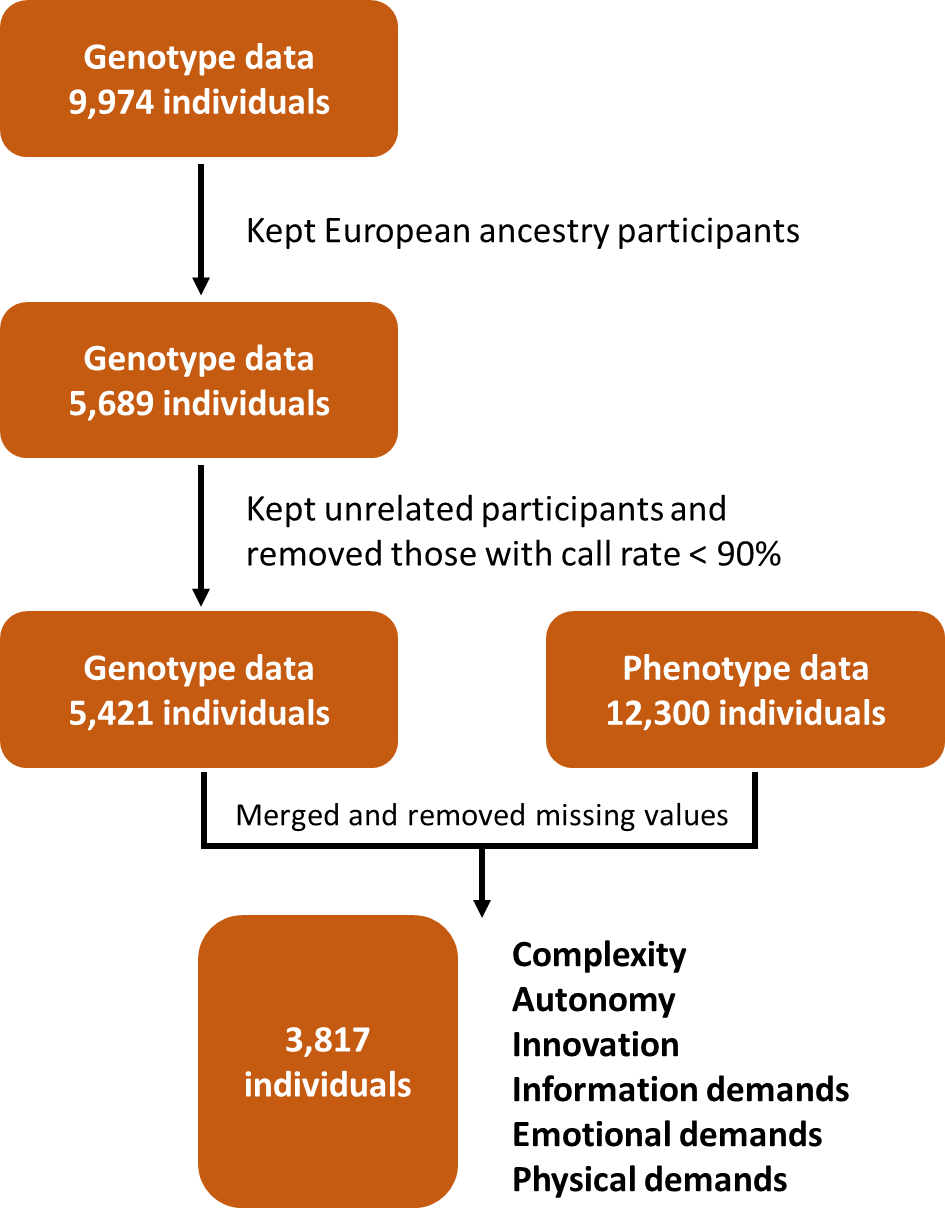


**Figure S8. Data preparation for the Add Health Wave V cohort.** The replication stage includes unrelated individuals of European ancestry from the Add Health Wave V data, with imputed markers, referenced to the Haplotype Reference Consortium panel (HRCr1.1). Before performing imputation, Add Health applied a stringent QC procedure on genotyped data. 3,817 individuals with 6 phenotypes data were available for single-variant association tests.

**Supplementary Tables**

**Table S1: Job attainment characteristics from the O*NET database occupation code**

| **Phenotype**  **(reference)** | | **Coding** | | **# of items** | | **Item** |
| --- | --- | --- | --- | --- | --- | --- |
| Complexity  (*5*) | |  | | 120 | |  |
|  | | 7-point scale:  1 = lowest level,  7 = highest level | | 33 | | Knowledge (33 specific areas of knowledge required in a job)  What level of the following knowledge is needed to perform your current job?   1. Administration and Management: Knowledge of business and management principles involved in strategic planning, resource allocation, human resources modeling, leadership technique, production methods, and coordination of people and resources. 2. Clerical: Knowledge of administrative and clerical procedures and systems such as word processing, managing files and records, stenography and transcription, designing forms, and other office procedures and terminology. 3. Economics and Accounting: Knowledge of economic and accounting principles and practices, the financial markets, banking, and the analysis and reporting of financial data. 4. Sales and Marketing: Knowledge of principles and methods for showing, promoting, and selling products or services. This includes marketing strategy and tactics, product demonstration, sales techniques, and sales control systems. 5. Customer and Personal Service: Knowledge of principles and processes for providing customer and personal services. This includes customer needs assessment, meeting quality standards for services, and evaluation of customer satisfaction. 6. Personnel and Human Resources: Knowledge of principles and procedures for personnel recruitment, selection, training, compensation and benefits, labor relations and negotiation, and personnel information systems. 7. Production and Processing: Knowledge of raw materials, production processes, quality control, costs, and other techniques for maximizing the effective manufacture and distribution of goods. 8. Food Production: Knowledge of techniques and equipment for planting, growing, and harvesting food products (both plant and animal) for consumption, including storage/handling techniques. 9. Computers and Electronics: Knowledge of circuit boards, processors, chips, electronic equipment, and computer hardware and software, including applications and programming. 10. Engineering and Technology: Knowledge of the practical application of engineering science and technology. This includes applying principles, techniques, procedures, and equipment to the design and production of various goods and services. 11. Design: Knowledge of design techniques, tools, and principles involved in the production of precision technical plans, blueprints, drawings, and models. 12. Building and Construction: Knowledge of materials, methods, and the tools involved in the construction or repair of houses, buildings, or other structures such as highways and roads. 13. Mechanical: Knowledge of machines and tools, including their designs, uses, repair, and maintenance. 14. Mathematics: Knowledge of arithmetic, algebra, geometry, calculus, statistics, and their applications. 15. Physics: Knowledge and prediction of physical principles, laws, their interrelationships, and applications to understanding fluid, material, and atmospheric dynamics, and mechanical, electrical, atomic, and sub-atomic structures and processes. 16. Chemistry: Knowledge of the chemical composition, structure, and properties of substances and of the chemical processes and transformations that they undergo. This includes uses of chemicals and their interactions, danger signs, production techniques, and disposal methods. 17. Biology: Knowledge of plant and animal organisms, their tissues, cells, functions, interdependencies, and interactions with each other and the environment. 18. Psychology: Knowledge of human behavior and performance; individual differences in ability, personality, and interests; learning and motivation; psychological research methods; and the assessment and treatment of behavioral and affective disorders. 19. Sociology and Anthropology: Knowledge of group behavior and dynamics, societal trends and influences, human migrations, ethnicity, cultures, and their history and origins. 20. Geography: Knowledge of principles and methods for describing the features of land, sea, and air masses, including their physical characteristics, locations, interrelationships, and distribution of plant, animal, and human life. 21. Medicine and Dentistry: Knowledge of the information and techniques needed to diagnose and treat human injuries, diseases, and deformities. This includes symptoms, treatment alternatives, drug properties and interactions, and preventive healthcare measures. 22. Therapy and Counseling: Knowledge of principles, methods, and procedures for diagnosis, treatment, and rehabilitation of physical and mental dysfunctions, and career counseling and guidance. 23. Education and Training: Knowledge of principles and methods for curriculum and training design, teaching and instruction for individuals and groups, and the measurement of training effects. 24. English Language: Knowledge of the structure and content of the English language including the meaning and spelling of words, rules of composition, and grammar. 25. Foreign Language: Knowledge of the structure and content of a foreign (non-English) language including the meaning and spelling of words, rules of composition and grammar, and pronunciation. 26. Fine Arts: Knowledge of the theory and techniques required to compose, produce, and perform works of music, dance, visual arts, drama, and sculpture. 27. History and Archeology: Knowledge of historical events and their causes, indicators, and effects on civilizations and cultures. 28. Philosophy and Theology: Knowledge of different philosophical systems and religions. This includes their basic principles, values, ethics, ways of thinking, customs, practices, and their impact on human culture. 29. Public Safety and Security: Knowledge of relevant equipment, policies, procedures, and strategies to promote effective local, state, or national security operations for the protection of people, data, property, and institutions. 30. Law and Government: Knowledge of laws, legal codes, court procedures, precedents, government regulations, executive orders, agency rules, and the democratic political process. 31. Telecommunications: Knowledge of transmission, broadcasting, switching, control, and operation of telecommunications systems. 32. Communications and Media: Knowledge of media production, communication, and dissemination techniques and methods. This includes alternative ways to inform and entertain via written, oral, and visual media. 33. Transportation: Knowledge of principles and methods for moving people or goods by air, rail, sea, or road, including the relative costs and benefits. |
|  | | |  | 35 | | Skills (35 specific skills required in a job)  What level of the following skills is needed to perform your current job?   1. Reading Comprehension: Understanding written sentences and paragraphs in work-related documents. 2. Active Listening: Giving full attention to what other people are saying, taking time to understand the points being made, asking questions as appropriate, and not interrupting at inappropriate times. 3. Writing: Communicating effectively in writing as appropriate for the needs of the audience. 4. Speaking: Talking to others to convey information effectively. 5. Mathematics: Using mathematics to solve problems. 6. Science: Using scientific rules and methods to solve problems. 7. Critical Thinking: Using logic and reasoning to identify the strengths and weaknesses of alternative solutions, conclusions or approaches to problems. 8. Active Learning: Understanding the implications of new information for both current and future problem-solving and decision-making. 9. Learning Strategies: Selecting and using training/instructional methods and procedures appropriate for the situation when learning or teaching new things. 10. Monitoring: Monitoring/Assessing performance of yourself, other individuals, or organizations to make improvements or take corrective action. 11. Social Perceptiveness: Being aware of others' reactions and understanding why they react as they do. 12. Coordination: Adjusting actions in relation to others' actions. 13. Persuasion: Persuading others to change their minds or behavior. 14. Negotiation: Bringing others together and trying to reconcile differences. 15. Instructing: Teaching others how to do something. 16. Service Orientation: Actively looking for ways to help people. 17. Complex Problem Solving: Identifying complex problems and reviewing related information to develop and evaluate options and implement solutions. 18. Operations Analysis: Analyzing needs and product requirements to create a design. 19. Technology Design: Generating or adapting equipment and technology to serve user needs. 20. Equipment Selection: Determining the kind of tools and equipment needed to do a job. 21. Installation: Installing equipment, machines, wiring, or programs to meet specifications. 22. Programming: Writing computer programs for various purposes. 23. Operation Monitoring: Watching gauges, dials, or other indicators to make sure a machine is working properly. 24. Operation and Control: Controlling operations of equipment or systems. 25. Equipment Maintenance: Performing routine maintenance on equipment and determining when and what kind of maintenance is needed. 26. Troubleshooting: Determining causes of operating errors and deciding what to do about it. 27. Repairing: Repairing machines or systems using the needed tools. 28. Quality Control Analysis: Conducting tests and inspections of products, services, or processes to evaluate quality or performance. 29. Judgment and Decision Making: Considering the relative costs and benefits of potential actions to choose the most appropriate one. 30. Systems Analysis: Determining how a system should work and how changes in conditions, operations, and the environment will affect outcomes. 31. Systems Evaluation: Identifying measures or indicators of system performance and the actions needed to improve or correct performance, relative to the goals of the system. 32. Time Management: Managing one's own time and the time of others. 33. Management of Financial Resources: Determining how the money will be spent to get the work done, and accounting for these expenditures. 34. Management of Material Resources: Obtaining and seeing to the appropriate use of equipment, facilities, and materials needed to do certain work. 35. Management of Personnel Resources: Motivating, developing, and directing people as they work, identifying the best people for the job. |
|  | | |  | 52 | | Abilities (52 specific abilities required in a job, which can be classified into four categories: cognitive, physical, psychomotor, and sensory).  What level of the following abilities is needed to perform your current job?   1. Oral Comprehension: The ability to listen to and understand information and ideas presented through spoken words and sentences. 2. Written Comprehension: The ability to read and understand information and ideas presented in writing. 3. Oral Expression: The ability to communicate information and ideas in speaking so others will understand. 4. Written Expression: The ability to communicate information and ideas in writing so others will understand. 5. Fluency of Ideas: The ability to come up with a number of ideas about a topic (the number of ideas is important, not their quality, correctness, or creativity). 6. Originality: The ability to come up with unusual or clever ideas about a given topic or situation, or to develop creative ways to solve a problem. 7. Problem Sensitivity: The ability to tell when something is wrong or is likely to go wrong. It does not involve solving the problem, only recognizing there is a problem. 8. Deductive Reasoning: The ability to apply general rules to specific problems to produce answers that make sense. 9. Inductive Reasoning: The ability to combine pieces of information to form general rules or conclusions (includes finding a relationship among seemingly unrelated events). 10. Information Ordering: The ability to arrange things or actions in a certain order or pattern according to a specific rule or set of rules (e.g., patterns of numbers, letters, words, pictures, mathematical operations). 11. Category Flexibility: The ability to generate or use different sets of rules for combining or grouping things in different ways. 12. Mathematical Reasoning: The ability to choose the right mathematical methods or formulas to solve a problem. 13. Number Facility: The ability to add, subtract, multiply, or divide quickly and correctly. 14. Memorization: The ability to remember information such as words, numbers, pictures, and procedures. 15. Speed of Closure: The ability to quickly make sense of, combine, and organize information into meaningful patterns. 16. The flexibility of Closure: The ability to identify or detect a known pattern (a figure, object, word, or sound) that is hidden in other distracting material. 17. Perceptual Speed: The ability to quickly and accurately compare similarities and differences among sets of letters, numbers, objects, pictures, or patterns. The things to be compared may be presented at the same time or one after the other. This ability also includes comparing a presented object with a remembered object. 18. Spatial Orientation: The ability to know your location in relation to the environment or to know where other objects are in relation to you. 19. Visualization: The ability to imagine how something will look after it is moved around or when its parts are moved or rearranged. 20. Selective Attention: The ability to concentrate on a task over a period of time without being distracted. 21. Time-Sharing: The ability to shift back and forth between two or more activities or sources of information (such as speech, sounds, touch, or other sources). 22. Arm-Hand Steadiness: The ability to keep your hand and arm steady while moving your arm or while holding your arm and hand in one position. 23. Manual Dexterity: The ability to quickly move your hand, your hand together with your arm, or your two hands to grasp, manipulate, or assemble objects. 24. Finger Dexterity: The ability to make precisely coordinated movements of the fingers of one or both hands to grasp, manipulate, or assemble very small objects. 25. Control Precision: The ability to quickly and repeatedly adjust the controls of a machine or a vehicle to exact positions. 26. Multilimb Coordination: The ability to coordinate two or more limbs (for example, two arms, two legs, or one leg and one arm) while sitting, standing, or lying down. It does not involve performing the activities while the whole body is in motion. 27. Response Orientation: The ability to choose quickly between two or more movements in response to two or more different signals (lights, sounds, pictures). It includes the speed with which the correct response is started with the hand, foot, or other body parts. 28. Rate Control: The ability to time your movements or the movement of a piece of equipment in anticipation of changes in the speed and/or direction of a moving object or scene. 29. Reaction Time: The ability to quickly respond (with the hand, finger, or foot) to a signal (sound, light, picture) when it appears. 30. Wrist-Finger Speed: The ability to make fast, simple, repeated movements of the fingers, hands, and wrists. 31. Speed of Limb Movement: The ability to quickly move the arms and legs. 32. Static Strength: The ability to exert maximum muscle force to lift, push, pull, or carry objects. 33. Explosive Strength: The ability to use short bursts of muscle force to propel oneself (as in jumping or sprinting), or to throw an object. 34. Dynamic Strength: The ability to exert muscle force repeatedly or continuously over time. This involves muscular endurance and resistance to muscle fatigue. 35. Trunk Strength: The ability to use your abdominal and lower back muscles to support part of the body repeatedly or continuously over time without 'giving out' or fatiguing. 36. Stamina: The ability to exert yourself physically over long periods of time without getting winded or out of breath. 37. Extent Flexibility: The ability to bend, stretch, twist, or reach with your body, arms, and/or legs. 38. Dynamic Flexibility: The ability to quickly and repeatedly bend, stretch, twist, or reach out with your body, arms, and/or legs. 39. Gross Body Coordination: The ability to coordinate the movement of your arms, legs, and torso together when the whole body is in motion. 40. Gross Body Equilibrium: The ability to keep or regain your body balance or stay upright when in an unstable position. 41. Near Vision: The ability to see details at close range (within a few feet of the observer). 42. Far Vision: The ability to see details at a distance. 43. Visual Color Discrimination: The ability to match or detect differences between colors, including shades of color and brightness. 44. Night Vision: The ability to see under low light conditions. 45. Peripheral Vision: The ability to see objects or movement of objects to one's side when the eyes are looking ahead. 46. Depth Perception: The ability to judge which of several objects is closer or farther away from you, or to judge the distance between you and an object. 47. Glare Sensitivity: The ability to see objects in the presence of glare or bright lighting. 48. Hearing Sensitivity: The ability to detect or tell the differences between sounds that vary in pitch and loudness. 49. Auditory Attention: The ability to focus on a single source of sound in the presence of other distracting sounds. 50. Sound Localization: The ability to tell the direction from which a sound originated. 51. Speech Recognition: The ability to identify and understand the speech of another person. 52. Speech Clarity: The ability to speak clearly so others can understand you. |
| Autonomy (*6, 7*) | | 5-point scale:  1 = no freedom 3 = limited freedom  5 = a lot of freedom. | | | 2 | 1. How much freedom do you have to determine tasks, priorities, or goals of your current job? |
|  |  |  |  |  |  | 2. How much freedom do you have to make decisions without supervision? |
| Innovation  (*8*) | | 5-point scale:  1 = not important,  5 = extremely important. | | | 1 | Innovation refers to the extent to which the job requires creativity and alternative thinking to develop new ideas for and answers to work-related problems  How important is innovation to the performance of your current job? |
| Information demands  (*9*) | | 7-point scale: .  1 = lowest level,  7 = highest level | | | 17 | What level of the following activities is needed to perform your current job?  1. Getting information: Observing, receiving, and otherwise obtaining information from all relevant sources. |
|  |  |  |  |  |  | 2. Monitor processes, materials, or surroundings: Monitoring and reviewing information from materials, events, or the environment, to detect or assess problems. |
|  |  |  |  |  |  | 3. Identifying objects, actions, and, events: Identifying information by categorizing, estimating, recognizing differences or similarities, and detecting changes in circumstances or events. |
|  | |  | |  | | 4. Estimating the quantifiable characteristics of products, events, or information: Estimating sizes, distances, and quantities; or determining time, costs, resources, or materials needed to perform a work activity. |
|  | |  | |  | | 5. Judging the qualities of things, services, or people: Assessing the value, importance, or quality of things or people. |
|  | |  | |  | | 6. Processing information: Compiling, coding, categorizing, calculating, tabulating, auditing, or verifying information or data. |
|  |  | | |  | | 7. Evaluating information to determine compliance with standards: Using relevant information and individual judgment to determine whether events or processes comply with laws, regulations, or standards. |
|  |  | | |  | | 8. Analyzing data or information: Identifying the underlying principles, reasons, or facts of information by breaking down information or data into separate parts. |
|  |  | | |  | | 9. Making decisions and solving problems: Analyzing information and evaluating results to choose the best solution and solve problems. |
|  |  | | |  | | 10. Thinking creatively: Developing, designing, or creating new applications, ideas, relationships, systems, or products, including artistic contributions. |
|  |  | | |  | | 11. Updating and using relevant knowledge: Keeping up-to-date technically and applying new knowledge to your job. |
|  |  | | |  | | 12. Interacting with computers: Using computers and computer systems (including hardware and software) to program, write software, set up functions, enter data, or process information. |
|  |  | | |  | | 13. Drafting, laying out, and specifying technical devices, parts, and equipment: Providing documentation, detailed instructions, drawings, or specifications to tell others about how devices, parts, equipment, or structures are to be fabricated, constructed, assembled, modified, maintained, or used. |
|  |  | | |  | | 14. Documenting/recording information: Entering, transcribing, recording, storing, or maintaining information in written or electronic/magnetic form. |
|  |  | | |  | | 15. Interpreting the meaning of information for others: Translating or explaining what information means and how it can be used. |
|  |  | | |  | | 16. Communicating with supervisors, peers, or subordinates: Providing information to supervisors, co-workers, and subordinates by telephone, in written form, e-mail, or in person. |
|  |  | | |  | | 17. Provide consultation and advice to others: Providing guidance and expert advice to management or other groups on technical, systems, or process-related topics. |
| Emotional demands  (*9*) | 7-point scale:  1 = lowest level  7 = highest level | | | 5 | | What level of the following activities is needed to perform your current job?  1. Assisting and caring for others: Providing personal assistance, medical attention, emotional support, or other personal care to others such as coworkers, customers, or patients. |
|  |  |  |  |  | | 2. Performing for or working directly with the public: Performing for people or dealing directly with the public. This includes serving customers in restaurants and stores and receiving clients or guests. |
|  |  |  |  |  | | 3. Deal with external customers: How important is it to work with external customers or the public in this job? |
|  |  | | |  | | 4. Frequency of conflict situations: How often are there conflict situations the employee has to face in this job? |
|  |  | | |  | | 5. Deal with unpleasant or angry people: How frequently does the worker have to deal with unpleasant, angry, or discourteous individuals as part of the job requirements? |
| Physical demands  (*9*) | 7-point scale:  1 = lowest level  7 = highest level | | | 12 | | What level of the following activities is needed to perform your current job?  1. Performing general physical activities: Performing physical activities that require considerable use of your arms and legs and moving your whole body, such as climbing, lifting, balancing, walking, stooping, and handling of materials. |
|  |  |  |  |  | | 2. Operating vehicles, mechanized devices, or equipment: Running, maneuvering, navigating, or driving vehicles or mechanized equipment, such as forklifts, passenger vehicles, aircraft, or watercraft. |
|  |  |  |  |  | | 3. Repairing and maintaining mechanical equipment: Servicing, repairing, adjusting, and testing machines, devices, moving parts, and equipment that operate primarily on the basis of mechanical (not electronic) principles. |
|  |  | | |  | | 4. Sounds, noise levels are distracting or uncomfortable: How often does this job require working exposed to sounds and noise levels that are distracting or uncomfortable? |
|  |  | | |  | | 5. Very hot or cold temperatures: How often does this job require working in very hot (above 90 F degrees) or very cold (below 32 F degrees) temperatures? |
|  |  | | |  | | 6. Extremely bright or inadequate lighting: How often does this job require working in extremely bright or inadequate lighting conditions? |
|  |  | | |  | | 7. Exposed to contaminants: How often does this job require working exposed to contaminants (such as pollutants, gases, dust, or odors)? |
|  |  | | |  | | 8. Cramped workspace, awkward positions: How often does this job require working in cramped workspaces that requires getting into awkward positions? |
|  |  | | |  | | 9. Exposed to whole-body vibration: How often does this job require exposure to whole-body vibration (e.g., operate a jackhammer)? |
|  |  | | |  | | 10. Exposed to high places: How often does this job require exposure to high places? |
|  |  | | |  | | 11. Exposed to hazardous conditions: How often does this job require exposure to hazardous conditions? |
|  |  | | |  | | 12. Exposed to hazardous equipment: How often does this job require exposure to hazardous equipment? |

*Note.* We summed these specific ratings to form an overall composite index of each phenotype.

**Table S2. λ_GC_, LDSC-intercept and SNP heritability estimations for job attainment traits in the UK Biobank data**

| Phenotype | λ_GC_ | LDSC Intercept (s.e.) | SNP *h^2^* (s.e.) |
| --- | --- | --- | --- |
| Complexity | 1.096 | 1.026 (0.008) | 0.039 (0.003) |
| Autonomy | 1.096 | 1.016 (0.007) | 0.054 (0.003) |
| Innovation | 1.096 | 1.024 (0.008) | 0.058 (0.003) |
| Information demands | 1.147 | 1.033 (0.008) | 0.077 (0.004) |
| Emotional demands | 1.047 | 1.009 (0.007) | 0.016 (0.003) |
| Physical demands | 1.147 | 1.043 (0.008) | 0.061 (0.004) |

LDSC -linkage disequilibrium score regression

λ_GC_ - Genomic control inflation factor

SNP *h*^2^ - Common SNP heritability calculated using LDSC regression

**Table S3. Top loci identified for six job attainment characteristics from the UK Biobank GWAS**

| **Locus** | **Variant** | **CHR** | **Position** | **Function** | **Nearest gene** | **A1/A2** | **EAF** | **β** | **SE** | ***P*** | **Phenotype** |
| --- | --- | --- | --- | --- | --- | --- | --- | --- | --- | --- | --- |
| 1 | rs13019832 | 2 | 60710571 | intronic | BCL11A | G/A | 0.581 | 0.012 | 0.002 | 8.4E-10 | Physical demands |
| 2 | rs1437971 | 2 | 100986964 | intergenic | LONRF2; CHST10 | A/C | 0.351 | 0.012 | 0.002 | 2.2E-09 | Physical demands |
| 3 | rs55769536 | 2 | 212634084 | intronic | ERBB4 | A/T | 0.697 | -0.014 | 0.002 | 3.0E-11 | Physical demands |
| 4 | rs71627385 | 3 | 49432526 | intronic | RHOA | C/CA | 0.696 | -0.009 | 0.001 | 1.6E-13 | Autonomy |
|  | rs6446187 | 3 | 49907111 | intronic | CAMKV | C/A | 0.486 | 0.019 | 0.002 | 1.9E-16 | Information demands |
|  | rs1062633 | 3 | 49924940 | exonic | MST1R | T/C | 0.482 | 0.006 | 0.001 | 5.4E-10 | Complexity |
|  | rs9848497 | 3 | 49951316 | intronic | MON1A | T/C | 0.482 | 0.009 | 0.001 | 1.1E-13 | Innovation |
| 5 | rs10223052 | 5 | 60800336 | intronic | ZSWIM6 | A/G | 0.355 | 0.016 | 0.002 | 3.5E-11 | Information demands |
| 6 | rs34321 | 5 | 88020319 | intronic | MEF2C | C/G | 0.409 | -0.012 | 0.002 | 4.4E-09 | Physical demands |
| 7 | rs28837979 | 5 | 176891460 | intronic | DBN1 | T/A | 0.442 | -0.012 | 0.002 | 4.7E-09 | Physical demands |
| 8 | rs1487441 | 6 | 98553894 | intergenic | MIR2113; POU3F2 | G/A | 0.515 | -0.018 | 0.002 | 2.1E-15 | Information demands |
|  | rs9375188 | 6 | 98555272 | intergenic | MIR2113; POU3F2 | C/T | 0.515 | -0.008 | 0.001 | 2.5E-13 | Autonomy |
|  | rs12206087 | 6 | 98582900 | intergenic | MIR2113; POU3F2 | G/A | 0.516 | 0.013 | 0.002 | 8.7E-11 | Physical demands |
| 9 | rs10244965 | 7 | 36999175 | intronic | ELMO1 | G/A | 0.888 | 0.011 | 0.002 | 1.2E-10 | Autonomy |
| 10 | rs542900493 | 8 | 143462829 | intronic | TSNARE1 | G/GC | 0.769 | 0.015 | 0.002 | 6.1E-10 | Physical demands |
| 11 | rs11793831 | 9 | 23362311 | intergenic | LINC01239; LOC101929563 | G/T | 0.584 | -0.018 | 0.002 | 3.3E-14 | Information demands |
| 12 | rs68119843 | 9 | 124604538 | intronic | TTLL11 | A/T | 0.444 | -0.015 | 0.002 | 6.2E-11 | Information demands |
|  | rs7032484 | 9 | 124617919 | intronic | TTLL11 | G/A | 0.445 | -0.006 | 0.001 | 7.1E-09 | Complexity |
| 13 | rs9783478 | 12 | 82344954 | intergenic | PPFIA2; LINC02426 | A/G | 0.674 | -0.008 | 0.001 | 8.3E-10 | Innovation |
| 14 | rs9537814 | 13 | 58371672 | intergenic | PCDH17; LINC02338 | T/G | 0.719 | 0.008 | 0.001 | 5.3E-09 | Innovation |
| 15 | rs5816357 | 16 | 26199501 | intergenic | HS3ST4; C16orf82 | A/AAAAC | 0.534 | -0.012 | 0.002 | 1.1E-09 | Physical demands |
| 16 | rs11663050 | 18 | 35201153 | intergenic | CELF4; MIR4318 | T/G | 0.344 | -0.006 | 0.001 | 2.1E-09 | Complexity |
|  | rs11661773 | 18 | 35226024 | intergenic | CELF4; MIR4318 | C/A | 0.291 | -0.016 | 0.003 | 1.2E-10 | Information demands |

Lead variants shown were genome-wide significant loci in subjects of the UK Biobank dataset of European ancestry. *p*-value threshold at *P* < 8.33 x 10^-9^ to account for multiple testing for 6 phenotypes. CHR, chromosome; Nearest gene in 200kb flanking the lead variant based on NCBI build 37; A1, effect allele; A2, reference allele; EAF, effect allele frequency; β, regression coefficient based on the effect allele A1; SE, standard error of β.

**Table S4. Genetic effects of top variants in subgroups by age.**

| Phenotype | Variant | CHR | Position | A1/A2 | age <= 55 | | | age >55 | | | P_diff |
| --- | --- | --- | --- | --- | --- | --- | --- | --- | --- | --- | --- |
|  |  |  |  |  | BETA | SE | P | BETA | SE | P |  |
| Autonomy | rs71627385 | 3 | 49432526 | C/CA | -0.01 | 0.002 | 1.60E-09 | -0.007 | 0.002 | 3.50E-05 | 0.145 |
| Autonomy | rs9375188 | 6 | 98555272 | C/T | -0.009 | 0.001 | 2.20E-10 | -0.006 | 0.002 | 9.50E-05 | 0.078 |
| Autonomy | rs10244965 | 7 | 36999175 | G/A | 0.011 | 0.002 | 3.40E-06 | 0.011 | 0.003 | 9.30E-06 | 0.454 |
| Complexity | rs1062633 | 3 | 49924940 | T/C | 0.006 | 0.001 | 1.70E-05 | 0.006 | 0.001 | 1.10E-05 | 0.394 |
| Complexity | rs7032484 | 9 | 125000000 | G/A | -0.007 | 0.001 | 1.40E-07 | -0.004 | 0.001 | 4.50E-03 | 0.063 |
| Complexity | rs11663050 | 18 | 35201153 | T/G | -0.006 | 0.001 | 5.90E-05 | -0.007 | 0.002 | 1.00E-05 | 0.323 |
| Information demands | rs6446187 | 3 | 49907111 | C/A | 0.021 | 0.003 | 6.60E-11 | 0.017 | 0.003 | 5.80E-07 | 0.179 |
| Information demands | rs10223052 | 5 | 60800336 | A/G | 0.015 | 0.003 | 7.80E-06 | 0.017 | 0.003 | 1.10E-06 | 0.343 |
| Information demands | rs1487441 | 6 | 98553894 | G/A | -0.019 | 0.003 | 6.50E-09 | -0.018 | 0.003 | 6.70E-08 | 0.447 |
| Information demands | rs11793831 | 9 | 23362311 | G/T | -0.016 | 0.003 | 4.60E-07 | -0.02 | 0.003 | 8.00E-09 | 0.256 |
| Information demands | rs68119843 | 9 | 125000000 | A/T | -0.021 | 0.003 | 1.40E-10 | -0.009 | 0.003 | 5.60E-03 | 7.26E-03 |
| Information demands | rs11661773 | 18 | 35226024 | C/A | -0.013 | 0.004 | 2.90E-04 | -0.02 | 0.004 | 3.50E-08 | 0.072 |
| Innovation | rs9848497 | 3 | 49951316 | T/C | 0.007 | 0.002 | 6.70E-05 | 0.012 | 0.002 | 5.70E-11 | 0.013 |
| Innovation | rs9783478 | 12 | 82344954 | A/G | -0.009 | 0.002 | 1.60E-07 | -0.007 | 0.002 | 6.70E-04 | 0.172 |
| Innovation | rs9537814 | 13 | 58371672 | T/G | 0.006 | 0.002 | 2.40E-03 | 0.011 | 0.002 | 1.20E-07 | 0.027 |
| Physical demands | rs13019832 | 2 | 60710571 | G/A | 0.013 | 0.003 | 1.80E-06 | 0.011 | 0.003 | 1.60E-04 | 0.320 |
| Physical demands | rs1437971 | 2 | 101000000 | A/C | 0.013 | 0.003 | 7.50E-06 | 0.012 | 0.003 | 1.60E-04 | 0.403 |
| Physical demands | rs55769536 | 2 | 213000000 | A/T | -0.014 | 0.003 | 6.80E-07 | -0.014 | 0.003 | 1.60E-05 | 0.420 |
| Physical demands | rs34321 | 5 | 88020319 | C/G | -0.013 | 0.003 | 7.00E-07 | -0.01 | 0.003 | 8.30E-04 | 0.180 |
| Physical demands | rs28837979 | 5 | 177000000 | T/A | -0.012 | 0.003 | 6.10E-06 | -0.011 | 0.003 | 1.00E-04 | 0.419 |
| Physical demands | rs12206087 | 6 | 98582900 | G/A | 0.015 | 0.003 | 4.30E-08 | 0.011 | 0.003 | 2.30E-04 | 0.158 |
| Physical demands | rs542900493 | 8 | 143000000 | G/GC | 0.017 | 0.003 | 2.60E-07 | 0.012 | 0.004 | 4.70E-04 | 0.183 |
| Physical demands | rs5816357 | 16 | 26199501 | A/AAAAC | -0.013 | 0.003 | 1.00E-06 | -0.011 | 0.003 | 1.70E-04 | 0.293 |

A1, effect allele; A2, reference allele; BETA, association effect based on A1; SE, standard error of BETA

**Table S5. Replication and meta-analysis results in UK Biobank and Add Health datasets**

|  |  | |  | Discovery | | |  |  | Replication | |  |  |  | Meta-analysis | |  |  |
| --- | --- | --- | --- | --- | --- | --- | --- | --- | --- | --- | --- | --- | --- | --- | --- | --- | --- |
| # | Variant | A1/A2 | | UKB (N=219,483) | | | UKB (N=30,837) | | Addhealth (N=3,817) | |  |  | | | | | |
|  |  |  | | EAF | BETA | SE | BETA | SE | BETA | SE | replication-P | Effect | SE | D | meta-P | Het I^2^ | Het-P |
| 1 | rs13019832 | G/A | | 0.58 | 0.012 | 0.002 | 0.009 | 0.005 | 0.014 | 0.016 |  | 0.012 | 0.002 | +++ | 1.39E-10 | 0 | 0.782 |
| 2 | rs1437971 | A/C | | 0.35 | 0.012 | 0.002 | 0.000 | 0.005 | 0.009 | 0.016 |  | 0.011 | 0.002 | +-+ | 3.18E-08 | 63.6 | 0.064 |
| 3 | rs55769536 | A/T | | 0.70 | -0.014 | 0.002 | 0.001 | 0.005 | -0.018 | 0.017 |  | -0.012 | 0.002 | -+- | 9.42E-10 | 75 | 0.018 |
| 4 | rs71627385 | C/CA | | 0.70 | -0.009 | 0.001 | -0.005 | 0.003 | -0.006 | 0.009 |  | -0.008 | 0.001 | --- | 4.56E-14 | 0 | 0.557 |
|  | rs6446187 | C/A | | 0.49 | 0.019 | 0.002 | 0.009 | 0.006 | -0.021 | 0.017 |  | 0.017 | 0.002 | ++- | 1.55E-15 | 74.7 | 0.019 |
|  | rs1062633 | T/C | | 0.48 | 0.006 | 0.001 | 0.001 | 0.003 | -0.011 | 0.008 |  | 0.005 | 0.001 | ++- | 8.46E-09 | 74.4 | 0.020 |
|  | rs9848497 | T/C | | 0.48 | 0.009 | 0.001 | 0.004 | 0.003 | -0.011 | 0.010 |  | 0.008 | 0.001 | ++- | 6.52E-13 | 67.8 | 0.045 |
| 5 | rs10223052 | A/G | | 0.36 | 0.016 | 0.002 | 0.013 | 0.006 | -0.005 | 0.017 | UKB followip* | 0.015 | 0.002 | ++- | 7.85E-12 | 0 | 0.446 |
| 6 | rs34321 | C/G | | 0.41 | -0.012 | 0.002 | -0.011 | 0.005 | 0.010 | 0.016 | UKB followip* | -0.011 | 0.002 | --+ | 4.88E-10 | 0 | 0.381 |
| 7 | rs28837979 | T/A | | 0.44 | -0.012 | 0.002 | -0.004 | 0.005 | 0.017 | 0.016 |  | -0.010 | 0.002 | --+ | 3.06E-08 | 63.1 | 0.067 |
| 8 | rs1487441 | G/A | | 0.52 | -0.018 | 0.002 | -0.013 | 0.006 | -0.017 | 0.016 | UKB followip* | -0.018 | 0.002 | --- | 1.21E-16 | 0 | 0.725 |
|  | rs9375188 | C/T | | 0.52 | -0.008 | 0.001 | -0.006 | 0.003 | -0.012 | 0.008 | UKB followip* | -0.008 | 0.001 | --- | 7.94E-15 | 0 | 0.763 |
|  | rs12206087 | G/A | | 0.52 | 0.013 | 0.002 | 0.021 | 0.005 | 0.025 | 0.015 | UKB followip** | 0.014 | 0.002 | +++ | 3.77E-15 | 37.9 | 0.200 |
| 9 | rs10244965 | G/A | | 0.89 | 0.011 | 0.002 | -0.002 | 0.004 | -0.009 | 0.013 |  | 0.009 | 0.002 | +-- | 1.52E-08 | 80.6 | 0.006 |
| 10 | rs542900493 | G/GC | | 0.77 | 0.015 | 0.002 | -0.001 | 0.006 | 0.012 | 0.019 |  | 0.013 | 0.002 | +-+ | 1.44E-08 | 69.5 | 0.038 |
| 11 | rs11793831 | G/T | | 0.58 | -0.018 | 0.002 | -0.007 | 0.006 | -0.001 | 0.017 |  | -0.016 | 0.002 | --- | 1.30E-13 | 49.8 | 0.137 |
| 12 | rs68119843 | A/T | | 0.44 | -0.015 | 0.002 | -0.003 | 0.006 | -0.016 | 0.016 |  | -0.014 | 0.002 | --- | 2.51E-10 | 48.3 | 0.144 |
|  | rs7032484 | G/A | | 0.45 | -0.006 | 0.001 | -0.002 | 0.003 | -0.015 | 0.007 | Addhealth* | -0.006 | 0.001 | --- | 2.88E-09 | 36.6 | 0.207 |
| 13 | rs9783478 | A/G | | 0.67 | -0.008 | 0.001 | -0.002 | 0.004 | -0.003 | 0.010 |  | -0.007 | 0.001 | --- | 3.62E-09 | 36.6 | 0.207 |
| 14 | rs9537814 | T/G | | 0.72 | 0.008 | 0.001 | 0.004 | 0.004 | 0.017 | 0.011 |  | 0.008 | 0.001 | +++ | 2.72E-09 | 0 | 0.382 |
| 15 | rs5816357 | A/AAAAC | | 0.53 | -0.012 | 0.002 | -0.003 | 0.005 | 0.002 | 0.016 |  | -0.011 | 0.002 | --+ | 5.41E-09 | 45 | 0.162 |
| 16 | rs11663050 | T/G | | 0.34 | -0.006 | 0.001 | 0.000 | 0.003 | -0.003 | 0.008 |  | -0.006 | 0.001 | --- | 1.36E-08 | 47 | 0.151 |

We performed a fixed-effect meta-analysis with standard to combine the genetic effects across UK Biobank discovery, UK Biobank follow-up, and the Add Health cohorts.

#, locus number; A1, effect allele; EAF, effect allele frequency; BETA, association effect based on A1; SE, standard error of BETA; D, direction.

*Phenotype, Physical and Information represent Physical demands and information demands, respectively. For replication p: *: replication-p <0.05; **replication-p < 0.001

Table S6: Between-sex genetic correlation for each job attainment phenotype

| Trait | *r_g_* | 95% CI | *P* |
| --- | --- | --- | --- |
| Complexity | 0.522 | (0.344, 0.700) | 1.40 × 10^-7^ |
| Autonomy | 0.901 | (0.781, 1.020) | 0.102 |
| Innovation | 0.916 | (0.782, 1.050) | 0.218 |
| Information demands | 0.895 | (0.801, 0.988) | 0.026 |
| Emotional demands | 0.760 | (0.378, 1.142) | 0.218 |
| Physical demands | 0.884 | (0.683, 1.086) | 0.261 |

Sample size: N=108,689 males; N = 110,794 females

Table S7. Sex-specific SNPs with significant genetic differences for job attainment phenotypes

| phenotype | SNP | chr | BP | gene | allele | FREQ | BETA | SE | P value | BETA | SE | P value | P_dif |
| --- | --- | --- | --- | --- | --- | --- | --- | --- | --- | --- | --- | --- | --- |
|  |  |  |  |  |  |  |  | Female |  |  | Male |  |  |
| *Female-specific loci* |  |  |  |  |  |  |  |  |  |  |  |  |  |
| Complexity | rs1906252 | 6 | 98550289 | *MIR2113; PNKY* | C/A | 0.51 | -0.008 | 0.001 | 4.60E-08 | -0.002 | 0.001 | 1.70E-01 | 1.08E-03 |
| Autonomy | rs12681489 | 8 | 139198704 | *FAM135B* | G/A | 0.86 | 0.013 | 0.002 | 1.70E-09 | -0.004 | 0.002 | 1.20E-01 | 7.26E-08 |
| Innovation | rs2567651 | 3 | 180973122 | *SOX2-OT* | C/A | 0.13 | -0.016 | 0.003 | 1.80E-09 | -0.003 | 0.003 | 2.10E-01 | 3.64E-04 |
| Information demands | rs143014164 | 11 | 49379054 | *FOLH1;LOC440040* | G/GTG | 0.59 | -0.022 | 0.004 | 4.80E-08 | 0.003 | 0.004 | 4.50E-01 | 4.92E-06 |
| Information demands | rs9574210 | 13 | 78866242 | *OBI1-AS1* | A/C | 0.48 | 0.020 | 0.003 | 3.40E-09 | -0.002 | 0.003 | 5.60E-01 | 1.97E-06 |
| Information demands | rs11661773 | 18 | 35226024 | *CELF4;MIR4318* | C/A | 0.29 | -0.025 | 0.004 | 1.00E-11 | -0.008 | 0.004 | 3.00E-02 | 4.73E-04 |
| *Male-specific loci* |  |  |  |  |  |  |  |  |  |  |  |  |  |
| Information demands | 2:10936626_TA_T | 2 | 10936626 | *PDIA6* | TA/T | 0.88 | -0.003 | 0.005 | 5.40E-01 | -0.029 | 0.005 | 2.90E-08 | 2.80E-04 |
| Information demands | 16:51179009_TA_T | 16 | 51179009 | *SALL1* | TA/T | 0.20 | 0.000 | 0.004 | 9.20E-01 | -0.024 | 0.004 | 9.90E-09 | 2.01E-05 |
| Physical demands | rs13019832 | 2 | 60710571 | *BCL11A* | G/A | 0.58 | 0.004 | 0.002 | 1.50E-02 | 0.020 | 0.004 | 1.30E-08 | 2.00E-05 |
| Physical demands | rs1437971 | 2 | 100986964 | *LONRF2; CHST10* | A/C | 0.35 | 0.003 | 0.002 | 6.80E-02 | 0.022 | 0.004 | 7.40E-09 | 4.29E-06 |
| Physical demands | rs55769536 | 2 | 212634084 | *ERBB4* | A/T | 0.70 | -0.005 | 0.002 | 8.00E-03 | -0.023 | 0.004 | 1.30E-09 | 6.61E-06 |
| Physical demands | rs773674547 | 3 | 49428822 | *RHOA* | CA/C | 0.69 | 0.002 | 0.002 | 3.10E-01 | 0.022 | 0.004 | 8.90E-09 | 9.07E-07 |
| Physical demands | rs5848905 | 3 | 50176259 | *SEMA3F-AS1* | A/AG | 0.55 | 0.002 | 0.002 | 3.50E-01 | 0.021 | 0.004 | 1.70E-08 | 1.25E-06 |
| Physical demands | 4:39668264_TA_T | 4 | 39668264 | *SMIM14;UBE2K* | TA/T | 0.91 | 0.001 | 0.003 | 8.20E-01 | 0.036 | 0.006 | 1.10E-08 | 1.95E-07 |
| Physical demands | rs34321 | 5 | 88020319 | *MEF2C* | C/G | 0.41 | -0.003 | 0.002 | 4.10E-02 | -0.020 | 0.004 | 3.50E-08 | 1.77E-05 |
| Physical demands | rs12206087 | 6 | 98582900 | *MIR2113;POU3F2* | G/A | 0.52 | 0.003 | 0.002 | 6.00E-02 | 0.023 | 0.004 | 2.00E-10 | 3.35E-07 |
| Physical demands | rs6961176 | 7 | 8118334 | *GLCCI1* | T/C | 0.81 | -0.001 | 0.002 | 5.80E-01 | -0.025 | 0.005 | 3.90E-08 | 1.05E-06 |
| Physical demands | rs542900493 | 8 | 143462829 | *TSNARE1* | G/GC | 0.77 | 0.005 | 0.002 | 1.70E-02 | 0.025 | 0.004 | 1.20E-08 | 1.65E-05 |
| Physical demands | rs246085 | 12 | 109558197 | *ACACB* | T/C | 0.93 | 0.000 | 0.003 | 9.80E-01 | -0.041 | 0.007 | 9.40E-09 | 1.04E-07 |
| Physical demands | rs5816357 | 16 | 26199501 | *HS3ST4;C16orf82* | A/AAA | 0.53 | -0.003 | 0.002 | 5.70E-02 | -0.021 | 0.004 | 2.80E-09 | 2.32E-06 |
| *Genome-wide top loci* | |  |  |  |  |  |  |  |  |  |  |  |  |
| Complexity | rs145172830 | 3 | 115318611 | *GAP43* | G/GA | 0.96 | -0.020 | 0.004 | 4.20E-07 | 0.013 | 0.004 | 3.30E-04 | 8.04E-10 |
| Information demands | rs145172830 | - | - | *-* | - | - | -0.044 | 0.009 | 4.40E-07 | 0.028 | 0.009 | 1.30E-03 | 5.05E-09 |
| Emotional demands | rs7326105 | 13 | 69183657 | *RPS3AP52* | A/G | 0.46 | -0.010 | 0.003 | 4.00E-05 | 0.011 | 0.003 | 4.50E-05 | 7.33E-09 |

A1, effect allele; A2, reference allele; FREQ, allele frequency; BETA, association effect based on A1; SE, standard error of BETA;

**Table S8. Polygenic risk score (PGS) associated with job attainment traits in testing samples**

| **Traits** | ***p*-value Threshold** | **PGS_R^2^** | **PGS_Beta** | **SE of beta** | ***P*** | **# of SNP included** |
| --- | --- | --- | --- | --- | --- | --- |
|  |  |  | UKB follow-up dataset ( N = 30,837) | |  |  |
| Complexity | 0.0710 | 0.0030 | 0.0187 | 0.0019 | 6.83E-24 | 282846 |
| Autonomy | 0.5100 | 0.0065 | 0.0148 | 0.0010 | 3.13E-46 | 1359448 |
| Innovation | 0.4400 | 0.0061 | 0.0155 | 0.0011 | 2.95E-43 | 1229531 |
| Information demands | 0.2500 | 0.0083 | 0.0186 | 0.0011 | 2.56E-62 | 817380 |
| Emotional demands | 0.0600 | 0.0015 | 0.0153 | 0.0021 | 3.05E-13 | 239204 |
| Physical demands | 0.5300 | 0.0050 | 0.0126 | 0.0010 | 2.48E-39 | 1396609 |
|  | Add Health data (N = 3,817) | | | | | |
| Complexity | 0.0940 | 0.0031 | 0.0309 | 0.0087 | 3.80E-04 | 125299 |
| Autonomy | 0.0680 | 0.0054 | 0.0435 | 0.0095 | 5.41E-06 | 100246 |
| Innovation | 0.0006 | 0.0043 | 0.1851 | 0.0456 | 4.94E-05 | 2237 |
| Information demands | 0.1700 | 0.0070 | 0.0356 | 0.0068 | 1.91E-07 | 208535 |
| Emotional demands | 0.3200 | 0.0027 | 0.0244 | 0.0074 | 9.79E-04 | 313827 |
| Physical demands | 0.0029 | 0.0091 | 0.1594 | 0.0248 | 1.45E-10 | 8841 |

GWAS result from Job attainment in UKB discovery sample was used for PGS construction, using PRsice 2.

*P* - model fitting *p*-values for PGS;

PGS_R^2^ was calculated as the difference between the R^2^ coefficients from the two regression models (covariates + PGS vs. covariates only).

The optimal model of the p-value threshold was selected using model fitting *P*.

For UKB follow-up data, the covariates include age, sex, array factors, and top 20 PCs. For Add health data, the covariates include age, sex, and top 10 PCs.

**Table S9. Pathway analysis for top implicated genes using g: Profiler**

| Source | Term name | Term id | Adjusted P-value | -log_10_ (Adjusted P value) | Term size | Query size | Intersection size | Effective domain size | intersections |
| --- | --- | --- | --- | --- | --- | --- | --- | --- | --- |
| GO:BP | **synapse organization** | GO:0050808 | 1.76E-04 | 3.75 | 432 | 18 | 7 | 18123 | ERBB4,RHOA,CAMKV,MEF2C,DBN1,PPFIA2,PCDH17 |
| GO:BP | positive regulation of growth | GO:0045927 | 2.88E-04 | 3.54 | 272 | 18 | 6 | 18123 | BCL11A,ERBB4,RHOA,MEF2C,DBN1,POU3F2 |
| GO:BP | positive regulation of developmental growth | GO:0048639 | 1.24E-03 | 2.91 | 182 | 18 | 5 | 18123 | BCL11A,ERBB4,MEF2C,DBN1,POU3F2 |
| GO:BP | **cell morphogenesis involved in neuron differentiation** | GO:0048667 | 1.76E-03 | 2.76 | 607 | 18 | 7 | 18123 | BCL11A,RHOA,ZSWIM6,MEF2C,DBN1,POU3F2,PPFIA2 |
| GO:BP | **regulation of synapse organization** | GO:0050807 | 2.64E-03 | 2.58 | 212 | 18 | 5 | 18123 | RHOA,CAMKV,MEF2C,DBN1,PPFIA2 |
| GO:BP | **regulation of synapse structure or activity** | GO:0050803 | 3.38E-03 | 2.47 | 223 | 18 | 5 | 18123 | RHOA,CAMKV,MEF2C,DBN1,PPFIA2 |
| GO:BP | **dendrite development** | GO:0016358 | 5.58E-03 | 2.25 | 247 | 18 | 5 | 18123 | BCL11A,RHOA,MEF2C,DBN1,PPFIA2 |
| GO:BP | cell junction organization | GO:0034330 | 5.85E-03 | 2.23 | 727 | 18 | 7 | 18123 | ERBB4,RHOA,CAMKV,MEF2C,DBN1,PPFIA2,PCDH17 |
| GO:BP | cell morphogenesis involved in differentiation | GO:0000904 | 7.45E-03 | 2.13 | 754 | 18 | 7 | 18123 | BCL11A,RHOA,ZSWIM6,MEF2C,DBN1,POU3F2,PPFIA2 |
| GO:BP | regulation of developmental growth | GO:0048638 | 2.56E-02 | 1.59 | 338 | 18 | 5 | 18123 | BCL11A,ERBB4,MEF2C,DBN1,POU3F2 |
| GO:BP | **regulation of axonogenesis** | GO:0050770 | 2.64E-02 | 1.58 | 159 | 18 | 4 | 18123 | BCL11A,RHOA,DBN1,POU3F2 |
| GO:CC | **glutamatergic synapse** | GO:0098978 | 1.06E-04 | 3.98 | 354 | 18 | 6 | 18964 | ERBB4,RHOA,CAMKV,DBN1,PPFIA2,PCDH17 |
| GO:CC | **postsynapse** | GO:0098794 | 1.90E-04 | 3.72 | 641 | 18 | 7 | 18964 | BCL11A,ERBB4,RHOA,MEF2C,DBN1,PPFIA2,PCDH17 |
| GO:CC | **synapse** | GO:0045202 | 2.48E-03 | 2.60 | 1348 | 18 | 8 | 18964 | BCL11A,ERBB4,RHOA,CAMKV,MEF2C,DBN1,PPFIA2,PCDH17 |
| REAC | PTK6 Regulates RHO GTPases, RAS GTPase and MAP kinases | REAC:R-HSA-8849471 | 1.69E-02 | 1.77 | 13 | 10 | 2 | 10622 | RHOA,ELMO1 |
| CORUM | ELMO1-DOCK1 complex | CORUM:5337 | 4.97E-02 | 1.30 | 2 | 2 | 1 | 3627 | ELMO1 |
| CORUM | MRIP-RHOA complex | CORUM:816 | 4.97E-02 | 1.30 | 2 | 2 | 1 | 3627 | RHOA |
| CORUM | ELMO1-DOCK2 complex | CORUM:5341 | 4.97E-02 | 1.30 | 2 | 2 | 1 | 3627 | ELMO1 |

**Table S10. GTEX gene enrichment analysis using FUMA**

| **Complexity** | **Tissue** | **Number of Genes** | **BETA** | **BETA STD** | **SE** | **P** | **Adj_P** |
| --- | --- | --- | --- | --- | --- | --- | --- |
|  | Kidney_Cortex | 17377 | 0.022 | 0.039 | 0.009 | 0.0076 |  |
|  | Kidney_Medulla | 17377 | 0.022 | 0.042 | 0.009 | 0.0081 |  |
|  | Ovary | 17377 | 0.019 | 0.038 | 0.010 | 0.0260 |  |
|  | Brain_Cortex | 17377 | 0.013 | 0.024 | 0.007 | 0.0321 |  |
|  | Fallopian_Tube | 17377 | 0.022 | 0.042 | 0.012 | 0.0333 |  |
|  | Brain_Frontal_Cortex_BA9 | 17377 | 0.011 | 0.021 | 0.007 | 0.0511 |  |
|  | Uterus | 17377 | 0.017 | 0.035 | 0.011 | 0.0587 |  |
|  | Brain_Cerebellum | 17377 | 0.009 | 0.018 | 0.006 | 0.0781 |  |
|  | Pituitary | 17377 | 0.012 | 0.023 | 0.009 | 0.0863 |  |
|  | Brain_Cerebellar_Hemisphere | 17377 | 0.007 | 0.014 | 0.006 | 0.1198 |  |
|  | Colon_Sigmoid | 17377 | 0.014 | 0.028 | 0.012 | 0.1235 |  |
|  | Brain_Anterior_cingulate_cortex_BA24 | 17377 | 0.008 | 0.014 | 0.007 | 0.1346 |  |
|  | Brain_Nucleus_accumbens_basal_ganglia | 17377 | 0.008 | 0.014 | 0.008 | 0.1389 |  |
|  | Brain_Hippocampus | 17377 | 0.008 | 0.013 | 0.008 | 0.1628 |  |
| **Autonomy** | **FULL_NAME** | **Number of Genes** | **BETA** | **BETA STD** | **SE** | **P** | **Adj_P** |
|  | Brain_Frontal_Cortex_BA9 | 17250 | 0.023 | 0.042 | 0.007 | 0.0008 | 0.0419 |
|  | Brain_Cortex | 17250 | 0.021 | 0.039 | 0.007 | 0.0021 |  |
|  | Brain_Cerebellar_Hemisphere | 17250 | 0.018 | 0.037 | 0.006 | 0.0022 |  |
|  | Brain_Cerebellum | 17250 | 0.019 | 0.037 | 0.007 | 0.0022 |  |
|  | Brain_Anterior_cingulate_cortex_BA24 | 17250 | 0.020 | 0.036 | 0.008 | 0.0039 |  |
|  | Brain_Hippocampus | 17250 | 0.017 | 0.028 | 0.008 | 0.0228 |  |
|  | Testis | 17250 | 0.011 | 0.019 | 0.006 | 0.0290 |  |
|  | Brain_Amygdala | 17250 | 0.015 | 0.025 | 0.008 | 0.0351 |  |
|  | Ovary | 17250 | 0.015 | 0.031 | 0.010 | 0.0637 |  |
|  | Brain_Nucleus_accumbens_basal_ganglia | 17250 | 0.011 | 0.019 | 0.008 | 0.0882 |  |
|  | Brain_Putamen_basal_ganglia | 17250 | 0.011 | 0.019 | 0.008 | 0.0907 |  |
|  | Brain_Spinal_cord_cervical_c-1 | 17250 | 0.011 | 0.020 | 0.009 | 0.1063 |  |
|  | Brain_Hypothalamus | 17250 | 0.010 | 0.018 | 0.008 | 0.1106 |  |
|  | Brain_Caudate_basal_ganglia | 17250 | 0.010 | 0.017 | 0.008 | 0.1113 |  |
|  | Pituitary | 17250 | 0.010 | 0.018 | 0.010 | 0.1544 |  |
| **Innovation** | **FULL_NAME** | **Number of Genes** | **BETA** | **BETA STD** | **SE** | **P** | **Adj_P** |
|  | Brain_Cortex | 17377 | 0.029 | 0.054 | 0.008 | 0.0001 | 0.0030 |
|  | Brain_Frontal_Cortex_BA9 | 17377 | 0.027 | 0.050 | 0.007 | 0.0001 | 0.0068 |
|  | Brain_Anterior_cingulate_cortex_BA24 | 17377 | 0.027 | 0.047 | 0.008 | 0.0003 | 0.0149 |
|  | Testis | 17377 | 0.019 | 0.032 | 0.006 | 0.0008 | 0.0453 |
|  | Brain_Cerebellum | 17377 | 0.019 | 0.038 | 0.007 | 0.0020 |  |
|  | Brain_Amygdala | 17377 | 0.024 | 0.040 | 0.008 | 0.0025 |  |
|  | Brain_Hippocampus | 17377 | 0.023 | 0.039 | 0.008 | 0.0032 |  |
|  | Brain_Cerebellar_Hemisphere | 17377 | 0.017 | 0.035 | 0.006 | 0.0035 |  |
|  | Brain_Nucleus_accumbens_basal_ganglia | 17377 | 0.020 | 0.035 | 0.008 | 0.0064 |  |
|  | Brain_Putamen_basal_ganglia | 17377 | 0.019 | 0.032 | 0.008 | 0.0137 |  |
|  | Ovary | 17377 | 0.023 | 0.046 | 0.010 | 0.0139 |  |
|  | Brain_Caudate_basal_ganglia | 17377 | 0.018 | 0.031 | 0.008 | 0.0161 |  |
|  | Brain_Hypothalamus | 17377 | 0.017 | 0.029 | 0.009 | 0.0242 |  |
|  | Pituitary | 17377 | 0.018 | 0.033 | 0.010 | 0.0348 |  |
|  | Brain_Spinal_cord_cervical_c-1 | 17377 | 0.009 | 0.017 | 0.009 | 0.1515 |  |
|  | Brain_Substantia_nigra | 17377 | 0.009 | 0.015 | 0.009 | 0.1696 |  |
| **Infromation demands** | **FULL_NAME** | **Number of Genes** | **BETA** | **BETA STD** | **SE** | **P** | **Adj_P** |
|  | Brain_Cerebellum | 17377 | 0.025 | 0.050 | 0.007 | 0.0001 | 0.0067 |
|  | Brain_Cerebellar_Hemisphere | 17377 | 0.023 | 0.047 | 0.007 | 0.0002 | 0.0122 |
|  | Brain_Cortex | 17377 | 0.026 | 0.047 | 0.008 | 0.0004 | 0.0228 |
|  | Brain_Frontal_Cortex_BA9 | 17377 | 0.024 | 0.045 | 0.008 | 0.0006 | 0.0340 |
|  | Brain_Anterior_cingulate_cortex_BA24 | 17377 | 0.022 | 0.039 | 0.008 | 0.0027 |  |
|  | Brain_Hippocampus | 17377 | 0.022 | 0.037 | 0.009 | 0.0065 |  |
|  | Brain_Amygdala | 17377 | 0.019 | 0.033 | 0.009 | 0.0124 |  |
|  | Pituitary | 17377 | 0.022 | 0.041 | 0.010 | 0.0139 |  |
|  | Brain_Nucleus_accumbens_basal_ganglia | 17377 | 0.018 | 0.031 | 0.008 | 0.0157 |  |
|  | Brain_Hypothalamus | 17377 | 0.019 | 0.032 | 0.009 | 0.0166 |  |
|  | Brain_Putamen_basal_ganglia | 17377 | 0.015 | 0.026 | 0.009 | 0.0381 |  |
|  | Testis | 17377 | 0.011 | 0.019 | 0.006 | 0.0390 |  |
|  | Brain_Caudate_basal_ganglia | 17377 | 0.015 | 0.026 | 0.009 | 0.0421 |  |
|  | Brain_Spinal_cord_cervical_c-1 | 17377 | 0.011 | 0.020 | 0.009 | 0.1114 |  |
|  | Brain_Substantia_nigra | 17377 | 0.008 | 0.014 | 0.009 | 0.1934 |  |
| **Emotional demands** | **FULL_NAME** | **Number of Genes** | **BETA** | **BETA STD** | **SE** | **P** | **Adj_P** |
|  | Uterus | 17250 | 0.015 | 0.030 | 0.011 | 0.0830 |  |
|  | Esophagus_Gastroesophageal_Junction | 17250 | 0.015 | 0.030 | 0.012 | 0.1066 |  |
|  | Cervix_Endocervix | 17250 | 0.013 | 0.026 | 0.011 | 0.1241 |  |
|  | Nerve_Tibial | 17250 | 0.011 | 0.022 | 0.010 | 0.1298 |  |
|  | Kidney_Medulla | 17250 | 0.010 | 0.019 | 0.009 | 0.1301 |  |
|  | Bladder | 17250 | 0.013 | 0.025 | 0.013 | 0.1558 |  |
|  | Cervix_Ectocervix | 17250 | 0.012 | 0.023 | 0.012 | 0.1621 |  |
|  | Kidney_Cortex | 17250 | 0.009 | 0.015 | 0.009 | 0.1626 |  |
|  | Esophagus_Muscularis | 17250 | 0.012 | 0.023 | 0.012 | 0.1693 |  |
|  | Thyroid | 17250 | 0.009 | 0.018 | 0.010 | 0.1716 |  |
|  | Heart_Atrial_Appendage | 17250 | 0.008 | 0.015 | 0.009 | 0.1860 |  |
|  | Ovary | 17250 | 0.008 | 0.017 | 0.009 | 0.1920 |  |
| **Physical demands** | **FULL_NAME** | **Number of Genes** | **BETA** | **BETA STD** | **SE** | **P** | **Adj_P** |
|  | Brain_Cerebellar_Hemisphere | 17377 | 0.027 | 0.055 | 0.007 | 0.0000 | 0.0008 |
|  | Brain_Cerebellum | 17377 | 0.027 | 0.054 | 0.007 | 0.0000 | 0.0016 |
|  | Brain_Hypothalamus | 17377 | 0.032 | 0.056 | 0.009 | 0.0001 | 0.0056 |
|  | Brain_Nucleus_accumbens_basal_ganglia | 17377 | 0.024 | 0.043 | 0.008 | 0.0014 |  |
|  | Brain_Frontal_Cortex_BA9 | 17377 | 0.022 | 0.040 | 0.007 | 0.0018 |  |
|  | Pituitary | 17377 | 0.028 | 0.053 | 0.010 | 0.0020 |  |
|  | Brain_Caudate_basal_ganglia | 17377 | 0.024 | 0.041 | 0.008 | 0.0023 |  |
|  | Brain_Hippocampus | 17377 | 0.024 | 0.041 | 0.009 | 0.0028 |  |
|  | Brain_Cortex | 17377 | 0.021 | 0.038 | 0.008 | 0.0031 |  |
|  | Brain_Spinal_cord_cervical_c-1 | 17377 | 0.024 | 0.044 | 0.009 | 0.0043 |  |
|  | Brain_Anterior_cingulate_cortex_BA24 | 17377 | 0.020 | 0.036 | 0.008 | 0.0046 |  |
|  | Testis | 17377 | 0.016 | 0.027 | 0.006 | 0.0049 |  |
|  | Brain_Substantia_nigra | 17377 | 0.024 | 0.041 | 0.009 | 0.0049 |  |
|  | Brain_Amygdala | 17377 | 0.022 | 0.037 | 0.009 | 0.0050 |  |
|  | Brain_Putamen_basal_ganglia | 17377 | 0.019 | 0.032 | 0.009 | 0.0132 |  |

The top tissues of gene enrichment p-value < 0.2 were listed for each job attainment traits. Adj_P value is the p-value adjusted for mulitple testing; significance is at adj_P <0.05.

**Table S11. Phenotypic and genetic correlation between job attainment and SES indices**

| **Job attainment** | **Educational attainment** | **Income** | **Educational attainment** |  | **Income** |  |
| --- | --- | --- | --- | --- | --- | --- |
|  | **Phenotypic correlation (*r*)** | | **Genetic correlation (*r_g_*)** | | |  |
| Complexity | 0.29 | 0.27 | 0.83 |  | 0.83 |  |
| Autonomy | 0.32 | 0.30 | 0.90 |  | 0.88 |  |
| Innovation | 0.39 | 0.23 | 0.92 |  | 0.81 |  |
| Information demands | 0.38 | 0.38 | 0.92 |  | 0.89 |  |
| Emotional demands | 0.11 | 0.04 | 0.37 |  | 0.44 |  |
| Physical demands | -0.20 | -0.17 | -0.91 |  | -0.79 |  |

For phenotypic correlation (Pearson correlation coefficients), educational attainment (Field 6138) and income (Field 738) variables were obtained from UK Biobank data. For genetic correlation, GWAS summary statistics for educational attainment (*10*) and income (*11*) were from published GWAS papers. LDSC regression method was applied.

**Table S12. Genetic correlation between job attainment and well-being, and correlation partialling out intelligence**

1. **Genetic correlation**

| **Phenotypes** | **Complexity** | | | | **Autonomy** | | | | **Innovation** | | | | **Information demands** | | | | **Emotional demands** | | | | **Physical demands** | | | |
| --- | --- | --- | --- | --- | --- | --- | --- | --- | --- | --- | --- | --- | --- | --- | --- | --- | --- | --- | --- | --- | --- | --- | --- | --- |
|  | ***r_g_*** | **SE** | **P** | **FDR** | ***r_g_*** | **SE** | **P** | **FDR** | ***r_g_*** | **SE** | **P** | **FDR** | ***r_g_*** | **SE** | **P** | **FDR** | ***r_g_*** | **SE** | **P** | **FDR** | ***r_g_*** | **SE** | **P** | **FDR** |
| Subjective well-being | 0.179 | 0.050 | 3.00E-04 | <0.05 | 0.133 | 0.043 | 1.9E-03 | <0.05 | 0.079 | 0.042 | 5.8E-02 |  | 0.152 | 0.040 | 1.0E-04 | <0.05 | 0.057 | 0.079 | 4.7E-01 |  | -0.089 | 0.045 | 4.8E-02 |  |
| Overall health rating | 0.513 | 0.034 | 1.68E-52 | <0.05 | 0.531 | 0.026 | 2.1E-90 | <0.05 | 0.514 | 0.027 | 7.6E-80 | <0.05 | 0.549 | 0.025 | 4.4E-111 | <0.05 | 0.165 | 0.050 | 9.0E-04 | <0.05 | -0.505 | 0.024 | 1.7E-97 | <0.05 |
| Job satisfaction | 0.064 | 0.075 | 3.94E-01 |  | 0.100 | 0.070 | 1.5E-01 |  | 0.048 | 0.066 | 4.7E-01 |  | 0.041 | 0.058 | 4.8E-01 |  | -0.026 | 0.102 | 8.0E-01 |  | -0.047 | 0.062 | 4.5E-01 |  |
| Depressive symptom | -0.388 | 0.053 | 1.56E-13 | <0.05 | -0.334 | 0.045 | 8.2E-14 | <0.05 | -0.314 | 0.043 | 1.9E-13 | <0.05 | -0.375 | 0.042 | 3.9E-19 | <0.05 | -0.119 | 0.076 | 1.2E-01 |  | 0.281 | 0.050 | 1.6E-08 | <0.05 |
| Neuroticism | -0.262 | 0.033 | 3.40E-15 | <0.05 | -0.243 | 0.030 | 8.0E-16 | <0.05 | -0.193 | 0.027 | 8.0E-13 | <0.05 | -0.249 | 0.026 | 1.2E-21 | <0.05 | -0.031 | 0.044 | 4.9E-01 |  | 0.166 | 0.027 | 7.8E-10 | <0.05 |
| Longevity | 0.158 | 0.079 | 4.55E-02 |  | 0.191 | 0.076 | 1.2E-02 | <0.05 | 0.130 | 0.071 | 6.5E-02 |  | 0.172 | 0.061 | 4.8E-03 | <0.05 | -0.038 | 0.111 | 7.3E-01 |  | -0.099 | 0.069 | 1.5E-01 |  |
| Number of cancer illness | -0.059 | 0.078 | 4.51E-01 |  | -0.086 | 0.074 | 2.5E-01 |  | -0.060 | 0.070 | 3.9E-01 |  | -0.067 | 0.068 | 3.3E-01 |  | 0.005 | 0.118 | 9.7E-01 |  | 0.048 | 0.066 | 4.7E-01 |  |
| Number of noncaner illness | -0.320 | 0.035 | 3.80E-20 | <0.05 | -0.300 | 0.031 | 8.1E-22 | <0.05 | -0.280 | 0.031 | 7.8E-20 | <0.05 | -0.313 | 0.028 | 1.0E-29 | <0.05 | -0.105 | 0.050 | 3.4E-02 |  | 0.239 | 0.030 | 1.3E-15 | <0.05 |
| BMI | -0.141 | 0.030 | 1.69E-06 | <0.05 | -0.198 | 0.026 | 2.3E-14 | <0.05 | -0.237 | 0.025 | 9.7E-22 | <0.05 | -0.183 | 0.023 | 2.1E-15 | <0.05 | 0.015 | 0.038 | 6.9E-01 |  | 0.260 | 0.023 | 6.1E-29 | <0.05 |
| Smoking initiation | -0.188 | 0.080 | 1.95E-02 | <0.05 | -0.211 | 0.073 | 3.9E-03 | <0.05 | -0.203 | 0.069 | 3.1E-03 | <0.05 | -0.179 | 0.066 | 6.3E-03 | <0.05 | 0.060 | 0.117 | 6.1E-01 |  | 0.213 | 0.074 | 3.8E-03 | <0.05 |

1. **Genetic correlation partialling out intelligence**

| **Phenotypes** | **Complexity** | | | | **Autonomy** | | | | **Innovation** | | | | **Information demands** | | | | **Emotional demands** | | | | **Physical demands** | | | |
| --- | --- | --- | --- | --- | --- | --- | --- | --- | --- | --- | --- | --- | --- | --- | --- | --- | --- | --- | --- | --- | --- | --- | --- | --- |
|  | ***r_g_*** | **SE** | **P** | **FDR** | ***r_g_*** | **SE** | **P** | **FDR** | ***r_g_*** | **SE** | **P** | **FDR** | ***r_g_*** | **SE** | **P** | **FDR** | ***r_g_*** | **SE** | **P** | **FDR** | ***r_g_*** | **SE** | **P** | **FDR** |
| Subjective well-being | 0.170 | 0.049 | 4.76E-04 | <0.05 | 0.122 | 0.041 | 2.6E-03 | <0.05 | 0.069 | 0.041 | 9.6E-02 |  | 0.140 | 0.037 | 1.6E-04 | <0.05 | 0.054 | 0.077 | 4.8E-01 |  | -0.075 | 0.040 | 6.1E-02 |  |
| Overall health rating | 0.311 | 0.035 | 1.12E-18 | <0.05 | 0.293 | 0.028 | 5.9E-26 | <0.05 | 0.264 | 0.029 | 3.0E-20 | <0.05 | 0.274 | 0.026 | 4.8E-26 | <0.05 | 0.125 | 0.045 | 5.5E-03 | <0.05 | -0.197 | 0.024 | 1.4E-16 | <0.05 |
| Job satisfaction | 0.050 | 0.071 | 4.82E-01 |  | 0.082 | 0.064 | 2.0E-01 |  | 0.036 | 0.061 | 5.5E-01 |  | 0.025 | 0.052 | 6.2E-01 |  | -0.033 | 0.099 | 7.4E-01 |  | -0.031 | 0.054 | 5.6E-01 |  |
| Depressive symptom | -0.253 | 0.053 | 2.06E-06 | <0.05 | -0.173 | 0.044 | 8.3E-05 | <0.05 | -0.142 | 0.040 | 4.3E-04 | <0.05 | -0.185 | 0.040 | 4.7E-06 | <0.05 | -0.095 | 0.073 | 1.9E-01 |  | 0.067 | 0.043 | 1.2E-01 |  |
| Neuroticism | -0.168 | 0.034 | 6.76E-07 | <0.05 | -0.131 | 0.029 | 5.1E-06 | <0.05 | -0.076 | 0.025 | 2.4E-03 | <0.05 | -0.120 | 0.025 | 1.1E-06 | <0.05 | -0.012 | 0.043 | 7.9E-01 |  | 0.023 | 0.024 | 3.4E-01 |  |
| Longevity | 0.091 | 0.072 | 2.05E-01 |  | 0.095 | 0.065 | 1.4E-01 |  | 0.039 | 0.060 | 5.2E-01 |  | 0.061 | 0.052 | 2.4E-01 |  | -0.038 | 0.103 | 7.1E-01 |  | 0.036 | 0.058 | 5.4E-01 |  |
| Number of cancer illness | -0.001 | 0.072 | 9.94E-01 |  | -0.025 | 0.069 | 7.2E-01 |  | 0.005 | 0.064 | 9.4E-01 |  | 0.011 | 0.062 | 8.6E-01 |  | 0.004 | 0.117 | 9.7E-01 |  | -0.028 | 0.061 | 6.5E-01 |  |
| Number of noncaner illness | -0.234 | 0.035 | 1.68E-11 | <0.05 | -0.197 | 0.028 | 7.7E-13 | <0.05 | -0.174 | 0.027 | 2.4E-10 | <0.05 | -0.198 | 0.025 | 6.6E-15 | <0.05 | -0.087 | 0.048 | 6.9E-02 |  | 0.110 | 0.026 | 2.6E-05 | <0.05 |
| BMI | -0.067 | 0.030 | 2.57E-02 | <0.05 | -0.108 | 0.025 | 1.9E-05 | <0.05 | -0.146 | 0.025 | 2.6E-09 | <0.05 | -0.081 | 0.022 | 1.8E-04 | <0.05 | 0.029 | 0.042 | 4.9E-01 |  | 0.149 | 0.021 | 1.8E-12 | <0.05 |
| Smoking initiation | -0.159 | 0.090 | 7.72E-02 |  | -0.164 | 0.085 | 5.4E-02 |  | -0.144 | 0.081 | 7.5E-02 |  | -0.108 | 0.071 | 1.3E-01 |  | 0.088 | 0.143 | 5.4E-01 |  | 0.130 | 0.086 | 1.3E-01 |  |

LDSC regression method was applied to calculate genetic correlation. Phenotype coding and GWAS sample size: Subjective wellbeing, continuous variable, 298,420 individuals; Overall health rating, categorical variable (1, 2, 3 and 4), 359,681 individuals; Job satisfaction, categorical variable (1 to 6), 82,190 individuals; Depressive symptom, continuous and binomial varaibles, 180,866 individuals; Neuroticism, continuous variable, 449,484 individuals; Longevity, binomial variable, 11,262 cases and 25,483 controls; Number of cancer illness, categorical variable (0, 1, 2, 3 and 4), 361,136 individuals; Number of noncaner illness, categorical variable (0, 1, and 2), 361,141 individuals; BMI, continuous variable, 683,365 individuals; Smoking initiation, binomial variable, 622,409 individuals; Intelligence, continuous variable, 269,867 individuals; Educational attainment, continuous variable, 766,345 individuals; Income, categorical variable, 286,301 individuals.

**Table S13. Genetic correlation between educational attainment, income, and well-being.**

| Phenotypes | Educational attainment |  | Income |  |
| --- | --- | --- | --- | --- |
|  | *r_g_* (SE) | *P* | *r_g_* (SE) | *P* |
| Subjective well-being | 0.109(0.029) | 2.00E-04 | 0.323 (0.039) | 5.07E-17 |
| Overall health rating | 0.554 (0.014) | 0 | 0.607 (0.021) | 2.87E-192 |
| Job satisfaction | 0.187 (0.048) | 8.89E-05 | 0.187 (0.048) | 8.89E-05 |
| Depressive symptom | -0.332 (0.031) | 2.22E-27 | -0.491 (0.04) | 1.42E-34 |
| Neuroticism | -0.231 (0.017) | 9.45E-44 | -0.281 (0.023) | 5.93E-35 |
| Longevity | 0.243 (0.046) | 1.02E-07 | 0.176 (0.059) | 2.80E-03 |
| Number of cancer illness | -0.089 (0.038) | 0.018 | -0.075 (0.061) | 0.22 |
| Number of noncaner illness | -0.306 (0.018) | 3.66E-67 | -0.345 (0.026) | 1.48E-40 |
| BMI | -0.268 (0.013) | 2.33E-08 | -0.212 (0.020) | 5.00E-03 |
| Smoking initiation | -0.267 (0.048) | 6.03E-90 | -0.171 (0.061) | 1.78E-26 |

GWAS summary statistics for well-being are the same as in Table S8. GWAS summary statistics for educational attainment (*12*) and income (*11*) were from published GWAS papers. LDSC regression method was applied.

**Table S14. Summary of O*NET linked phenotype scores in the UK Biobank follow-up dataset and Add Health dataset**

|  | **UKB follow-up dataset** | | **Add Health dataset** | |
| --- | --- | --- | --- | --- |
|  | N = 30,837 |  | N=3,817 |  |
| **Phenotype** | **Mean (s.d)** | **Range** | **Mean (s.d)** | **Range** |
| Complexity | 2.17 (0.35) | [1.13, 3.15] | 2.14 (0.33) | [1.13, 3.15] |
| Autonomy | 4.18 (0.34) | [2.69, 4.95] | 4.09 (0.37) | [2.83, 4.96] |
| Innovation | 3.64 (0.42) | [2.11, 4.62] | 3.54 (0.43) | [2.14, 4.63] |
| Information demands | 3.68 (0.75) | [1.53, 5.25] | 3.52 (0.72) | [1.53, 5.18] |
| Emotional demands | 3.30 (0.63) | [1.54, 4.76] | 3.32 (0.62) | [1.79, 4.99] |
| Physical demands | 1.72 (0.61) | [0.94, 4.31] | 1.91 (0.74) | [0.85, 4.49] |
| Age | 59.77 (6.82) | [40.00, 70.00] | 37.84 (1.85) | [33.67, 44.17] |
| Male, % | 43.40% |  | 43.90% |  |

**References**

1. C. Bycroft *et al.*, The UK Biobank resource with deep phenotyping and genomic data. *Nature* **562**, 203-209 (2018).

2. K. M. Harris, The add health study: Design and accomplishments. *Chapel Hill: Carolina Population Center, University of North Carolina at Chapel Hill*, 1-22 (2013).

3. H. M. Highland, C. L. Avery, Q. Duan, Y. Li, K. M. Harris, Quality control analysis of Add Health GWAS data. *Carolina Population Center, University of North Carolina at Chapel Hill, Chapel Hill, NC*, (2018).

4. J. Yang, S. H. Lee, M. E. Goddard, P. M. Visscher, GCTA: a tool for genome-wide complex trait analysis. *The American Journal of Human Genetics* **88**, 76-82 (2011).

5. T. A. Judge, B. A. Livingston, Is the gap more than gender? A longitudinal analysis of gender, gender role orientation, and earnings. *Journal of applied psychology* **93**, 994 (2008).

6. E. C. Dierdorff, J. K. Ellington, It's the nature of the work: examining behavior-based sources of work-family conflict across occupations. *Journal of Applied Psychology* **93**, 883-892 (2008).

7. E. C. Dierdorff, F. P. Morgeson, Consensus in work role requirements: the influence of discrete occupational context on role expectations. *Journal of Applied Psychology* **92**, 1228-1241 (2007).

8. T. A. Judge, C. P. Zapata, The person–situation debate revisited: Effect of situation strength and trait activation on the validity of the Big Five personality traits in predicting job performance. *Academy of Management Journal* **58**, 1149-1179 (2015).

9. T. M. Glomb, J. D. Kammeyer-Mueller, M. Rotundo, Emotional labor demands and compensating wage differentials. *Journal of Applied Pychology* **89**, 700-714 (2004).

10. J. J. Lee *et al.*, Gene discovery and polygenic prediction from a 1.1-million-person GWAS of educational attainment. *Nature Genetics* **50**, 1112-1121 (2018).

11. W. D. Hill *et al.*, Genome-wide analysis identifies molecular systems and 149 genetic loci associated with income. *Nature Communications* **10**, 573691 (2019).

12. J. E. Savage *et al.*, Genome-wide association meta-analysis in 269,867 individuals identifies new genetic and functional links to intelligence. *Nature Genetics* **50**, 912-919 (2018).
